# Supplementary material for: Complement factor H targeting antibody GT103 in refractory non-small cell lung cancer: a phase 1b dose escalation trial
Source: Nat Commun. 2025 Jan 2;16:93. doi: 10.1038/s41467-024-55092-2 (PMC11695982; doi:10.1038/s41467-024-55092-2)
Supplement: Supplementary file 1 — Supplementary Information [file 41467_2024_55092_MOESM1_ESM.pdf]

Supplementary Information

Supplementary Table 1. Molecular characteristics by treatment cohort

		Dose level (mg/kg)					
	**Total (N=31)**	**0.3 mg/kg Q3Wk (N=6)**	**1 mg/kg Q3Wk (N=6)**	**3 mg/kg Q3Wk (N=3)**	**10 mg/kg Q3Wk (N=6)**	**10 mg/kg Q2Wk (N=5)**	**15 mg/kg Q3Wk (N=5)**
**PD-L1 (%)**							
**N**	25	4	6	3	4	5	3
**Median**	2	20	2	10	4	1	0
**IQR**	0, 15	5, 55	0, 20	0, 60	1, 48	1, 5	0, 1
**Range**	0, 90	0, 80	0, 60	0, 60	0, 90	0, 15	0, 1
**PD-L1, n**							
**≤ 1%**	12	1	3	1	1	3	3
**> 1%**	13	3	3	2	3	2	0
**Not tested**	6	2	0	0	2	0	2
**ALK, n**							
**Negative**	20	4	6	1	2	4	3
**Not tested**	11	2	0	2	4	1	2
**ROS1, n**							
**Negative**	18	3	6	1	2	4	2
**Not tested**	13	3	0	2	4	1	3
**P53, n**							
**Positive**	6	1	2	1	1	1	0
**Not tested**	25	5	4	2	5	4	5
**KRAS, n**							
**Positive**	6	0	1	0	1	2	2
**Negative**	16	4	5	1	3	2	1

**Not tested**	9	2	0	2	2	1	2
**EGFR, n**							
**Positive**	5	1	1	0	1	0	2
**Negative**	18	4	5	1	2	4	2
**Not tested**	8	1	0	2	3	1	1
**KEAP1, n**							
**Positive**	1	0	1	0	0	0	0
**Negative**	3	1	0	1	0	1	0
**Not tested**	27	5	5	2	6	4	5
**STK11, n**							
**Positive**	3	0	2	0	1	0	0
**Negative**	3	1	0	1	0	1	0
**Not tested**	25	5	4	2	5	4	5
**TMB, n**							
**High**	5	1	2	0	1	1	0
**Low**	7	2	2	1	0	2	0
**Not tested**	19	3	2	2	5	2	5

Supplementary Table 2. Adverse Events (AE) regardless of attribution

		Adverse Event Grade		
	Total (N=21)	1 (N=1)	2 (N=4)	3 (N=16)
**AE Term**, no. of patients with maximum grade				

Abdominal pain	1	0	0	1
Acute kidney injury	1	0	0	1
Atrial flutter	1	0	0	1
Blood bilirubin increased	1	0	0	1
Colitis	2	0	2	0
Dyspnea	1	0	1	0
Esophageal stenosis	1	0	0	1
Fever	1	1	0	0
Hematuria	1	0	0	1
Hypoxia	1	0	0	1
Ileus	1	0	0	1
Lung infection	1	0	0	1
Non-cardiac chest pain	1	0	1	0
Pain	1	0	0	1
Pericardial effusion	1	0	0	1
Pleural effusion	1	0	0	1
Pneumonitis	1	0	0	1
Spinal fracture	1	0	0	1
Squamous Cell Carcinoma of Oropharynx	1	0	0	1
Tumor pain	1	0	0	1

Supplementary Table 3. Treatment duration by molecular profile

		**GT103 treatment duration (weeks)**			
**Mutation**	**No. Tested**	**Mean (SD)**	**Median (IQR)**	**Range**	**P***

**PD-L1**					0.08
PD-L1≤1	12	9.7 (11.3)	6.0 (6.0, 7.0)	3.0, 45.0	
PD-L1>1	13	14.3 (12.9)	9.0 (6.6, 19.1)	3.0, 50.9	
**KRAS**					0.91
Negative	16	11.2 (12.2)	6.4 (5.9, 10.0)	2.7, 50.9	
Positive	6	15.0 (15.9)	9.1 (4.4, 19.1)	3.0, 45.0	
**EGFR**					0.94
Negative	18	13.6 (14.1)	6.8 (5.9, 19.1)	2.7, 50.9	
Positive	5	7.8 (2.9)	6.1 (6.0, 8.0)	6.0, 12.7	
**KEAP1**					0.64
Negative	3	11.6 (12.7)	5.6 (3.0, 26.1)	3.0, 26.1	
Positive	1	3.0 (.)	3.0 (3.0, 3.0)	3.0, 3.0	
**STK11**					0.82
Negative	3	11.6 (12.7)	5.6 (3.0, 26.1)	3.0, 26.1	
Positive	3	4.5 (1.5)	4.4 (3.0, 6.0)	3.0, 6.0	
**TMB**					0.87

High	5	6.2 (0.6)	6.0 (6.0, 6.6)	5.4, 7.0
Low	7	8.9 (8.2)	6.0 (3.0, 12.3)	3.0, 26.1

*Wilcoxon rank sum 2 sided test.

Supplementary Table 4. Summary of progression-free survival (PFS) and overall survival (OS), stratified by molecular mutation status

**PD-L1**	**PDL1≤1**	**PDL1>1**	**Not Tested**
PFS			
Events / N	11/12	13/13	6/6
Median Time* (95% CI)	6.0 (5.1 - 7.0)	6.6 (5.4 - 17.1)	6.0 (2.4 - NE)
24-week PFS rate (95% CI)	8.3% (0.5 - 31.1)	15.4% (2.5 - 38.8)	0.0% (NE - NE)
OS			
Events / N	10/12	12/13	6/6
Median Time* (95% CI)	29.5 (8.4 - NE)	22.6 (13.9 - 76.3)	16.7 (6.3 - NE)
24-week OS rate (95% CI)	66.7% (33.7 – 86.0)	46.2% (19.2 - 69.6)	33.3% (4.6 - 67.6)
**KRAS**	**Positive**	**Negative**	**Not Tested**
PFS			
Events / N	5/6	16/16	9/9
Median Time* (95% CI)	9.1 (3.0 - NE)	6.1 (5.1 - 12.0)	6.0 (5.4 - NE)
24-week PFS rate (95% CI)	16.7% (0.8 - 51.7)	12.5% (2.1 - 32.8)	0.0% (NE - NE)
OS			
Events / N	4/6	15/16	9/9
Median Time* (95% CI)	33.9 (5.1 - NE)	21.1 (13.4 - 32.6)	25.7 (18.9 - 53.6)
24-week OS rate (95% CI)	66.7% (19.5 - 90.4)	43.8% (19.8 - 65.6)	55.6% (20.4 - 80.5)

**EGFR**	**Positive**	**Negative**	**Not Tested**
PFS	5/5	17/18	8/8
Events / N	6.0 (5.0 - NE)	6.8 (5.7 - 13.9)	6.0 (4.4 - 6.0)
Median Time* (95% CI)	0.0% (NE - NE)	16.7% (4.1 - 36.5)	0.0% (NE - NE)
24-week PFS rate (95% CI)			
OS			
Events / N	5/5	15/18	8/8
Median Time* (95% CI)	25.7 (18.9 - NE)	23.4 (13.4 – 39.1)	26.4 (9.9 - 53.6)
24-week OS rate (95% CI)	60.0% (12.6 - 88.2)	50.0% (25.9 - 70.1)	50.0% (15.2 - 77.5)
**KEAP1**	**Positive**	**Negative**	**Not Tested**
PFS			
Events / N	1/1	3/3	26/27
Median Time* (95% CI)	3.0 (NE - NE)	13.9 (4.6 - NE)	6.0 (6.0 - 6.1)
24-week PFS rate (95% CI)	0.0% (NE - NE)	33.3% (0.9 - 77.4)	7.4% (1.3 - 21.0)
OS			
Events / N	1/1	3/3	24/27
Median Time* (95% CI)	5.1 (NE - NE)	13.9 (7.6 - NE)	26.3 (19.6 - 35.7)
24-week OS rate (95% CI)	0.0% (NE - NE)	33.3% (0.9 - 77.4)	55.6% (35.2 - 71.8)
**STK11**	**Positive**	**Negative**	**Not Tested**
PFS			
Events / N	3/3	3/3	24/25
Median Time* (95% CI)	4.4 (3.0 - NE)	13.9 (4.6 - NE)	6.0 (6.0 - 6.6)
24-week PFS rate (95% CI)	0.0% (NE - NE)	33.3% (0.9 - 77.4)	8.0% (1.4 - 22.5)
OS			
Events / N	3/3	3/3	22/25
Median Time* (95% CI)	9.9 (5.1 - NE)	13.9 (7.6 - NE)	28.7 (20.4 – 39.1)
24-week OS rate (95% CI)	0.0% (NE - NE)	33.3% (0.9 - 77.4)	60.0% (38.4 - 76.1)
**TMB**	**High**	**Low**	**Not Tested**
PFS			
Events / N	5/5	7/7	18/19

Median Time* (95% CI)	6.0 (5.9 - NE)	6.1 (3.0 - 13.9)	6.0 (5.4 - 6.1)
24-week PFS rate (95% CI)	0.0% (NE - NE)	14.3% (0.7 - 46.5)	10.5% (1.8 - 28.4)
OS			
Events / N	5/5	6/7	17/19
Median Time* (95% CI)	26.3 (19.6 - NE)	14.6 (5.1 - 35.7)	25.7 (18.9 – 39.1)
24-week OS rate (95% CI)	60.0% (12.6 - 88.2)	42.9% (9.8 - 73.4)	52.6% (28.7 - 71.9)

*Weeks from initiation of GT-103

Supplementary Table 5: Summary of Plasma GT103 Exposure Parameters at Each Dose Level in Cycle 1

Dose Level	Descriptive Statistics	C_{max} (mcg/mL)	$AUC_{0\text{-infinity}}$ (mcg*h/mL)	$AUC_{0\text{-}336h}$ (mcg*h/mL)
0.3 mg/kg (n=6)	Min	4.82	703	595
	Median	5.63	980	717
	Max	8.10	1492	992
1 mg/kg (n=6)	Min	12.5	1111	1073
	Median	19.4	2446	2088
	Max	23.8	4646	3205
3 mg/kg (n=3)	Min	43.5	4718	4239
	Median	64.0	6011	5826
	Max	68.5	11558	8063
10 mg/kg	Min	154	8271	8190

(n=11)				
	Median	242	33041	27995
	Max	402	46696	33808
15 mg/kg (n=5)	Min	236	13884	13756
	Median	371	26360	27019
	Max	466	51256	52508

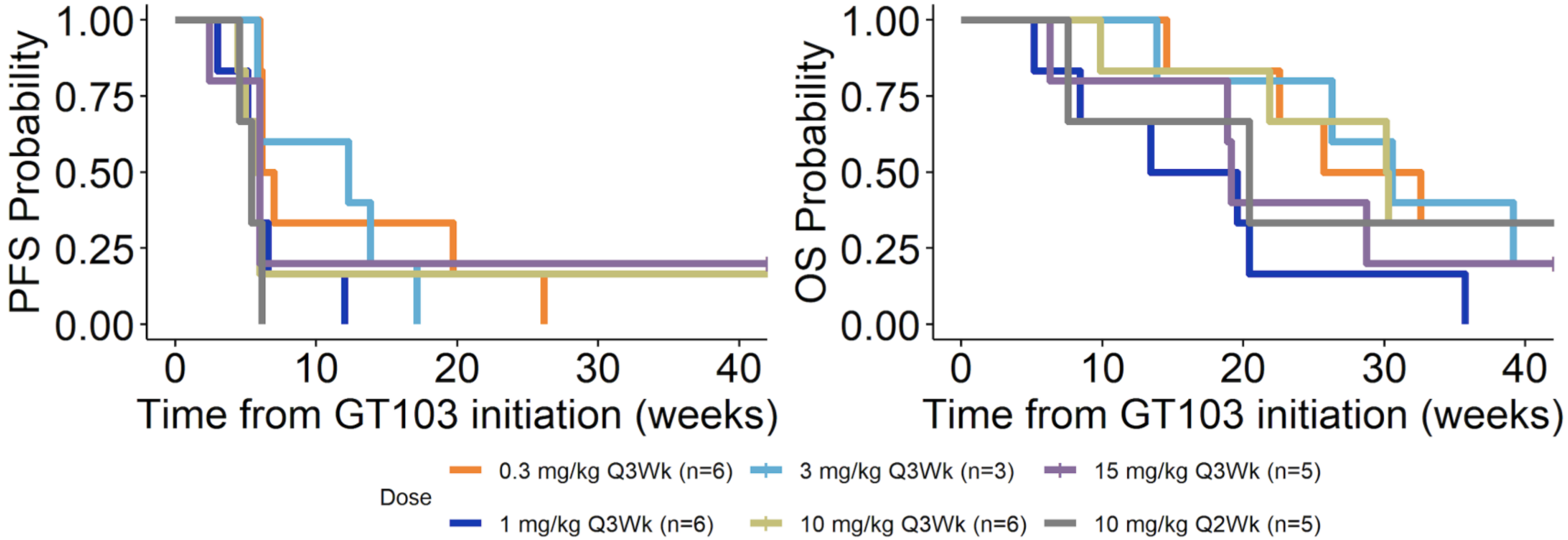

Supplementary Figure 1. Progression free survival (left) and overall survival (right) of patients treated with GT103 by dose cohort.

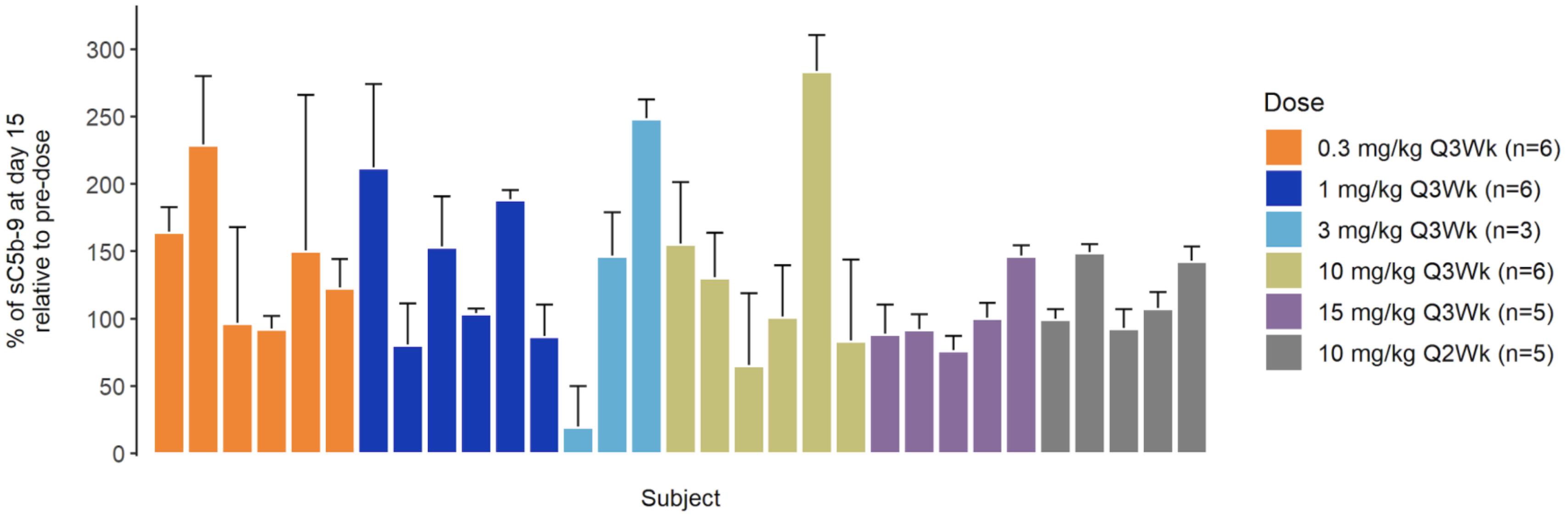

Supplementary Figure 2. Baseline and on-treatment modulation of soluble C5b-9 expressed on day 15 as % of predose levels. Each subject sample performed in triplicate.

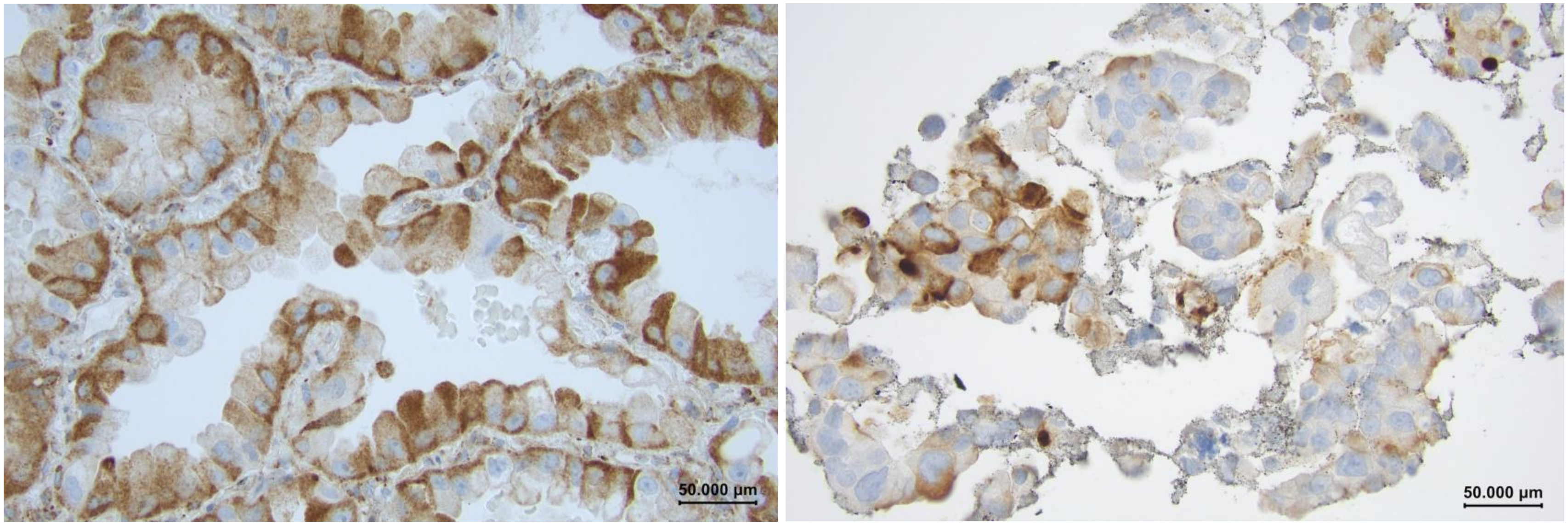

IHC	HR*	95% CI	p
CFH	1.002	0.995, 1.009	0.599
CD55	1.022	1.003, 1.043	0.0262

Supplementary Figure 3. Representative images staining for CFH (left) and CD55 (right) and correlation of IHC value and PFS. CFH (n = 19) and CD55 (n = 20) were analyzed separately using a Cox proportional hazard regression model.

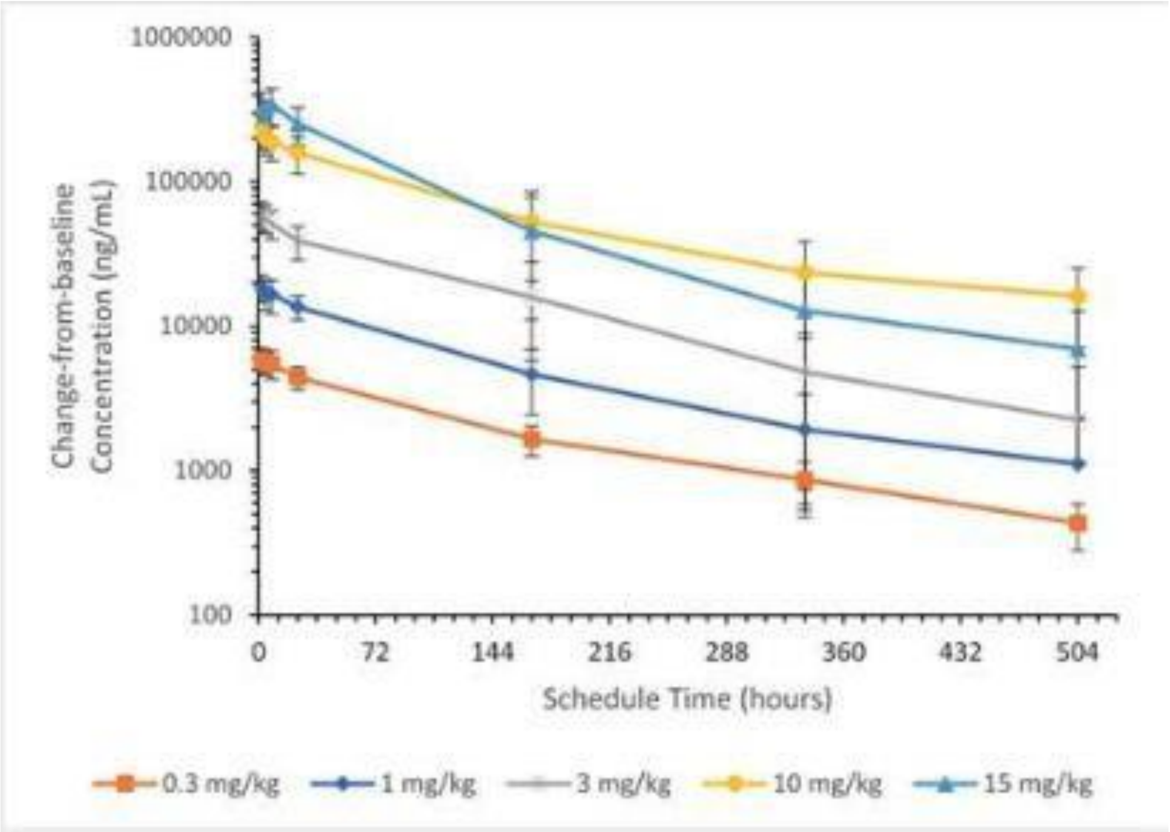

Supplementary Figure 4: Mean (Standard Deviation) Plasma GT03 Concentrations at Each Dose Level in Cycle 1: 0.3 mg/kg (n=6), 1 mg/kg (n=6), 3 mg/kg (n=3), 10 mg/kg (n=11), 15 mg/kg (n=5).

Phase Ib First in Human Dose Escalation of GT103 in Refractory, Advanced Stage Non-Small Cell Lung Cancer (TOP 1902)

DUKE CANCER INSTITUTE

A National Cancer Institute-designated Comprehensive Cancer Center

**Sponsor:**	PI – Duke Cancer Institute
**Funding Source:**	Grid Therapeutics, LLC
**Protocol Source:**	PI - Duke Cancer Institute
**Duke IRB#:**	Pro00104564
**IND#:**	124883

Principal Investigator
Jeffrey Clarke, MD
Duke Cancer Institute
Thoracic Oncology
Durham, NC 27710
919.681.9504
Fax: 919.684.5163
jeffrey.clarke@duke.edu

Sub-Investigator(s)
Neal Ready, MD, PhD
Scott Antonia, MD, PhD
Jeffrey Crawford, MD
Tom Stinchombe, MD
Jennifer Tenhover, ANP
Yeshu Conn, ANP
Susan Blackwell, PA-C
Deborah Ballard, ANP
Jennifer Garst, MD
Rebecca Phillips, NP
James Scott Smith, MD
Marvaretta Stevenson, MD
Sendhilnathan Ramalingam, MD

Regulatory Coordinator
Lucas David
Lucas.david@duke.edu

Statisticians
James E. Herndon II, PhD
James.herndon@duke.edu

Lin Gu, MS
Lin.gu@duke.edu

Project Manager
Carolyn Winters, RN, OCN, CCRC
MiSTiC (MultiSite Trials Service Center)
Duke Cancer Institute
DCI-MultiSiteStudies@duke.edu

Data Management
MiSTiC (MultiSite Trials Service Center)Duke Cancer Institute
DCI-MultiSiteStudies@duke.edu

The information contained in this document is regarded as confidential, and may not be disclosed to another party unless such disclosure is required to initiate the study, to conduct study-related activities, or to comply with national, state, or local laws and regulations. Written authorization from the coordinating site and sponsor is required for disclosure otherwise.

Protocol Versions

Version Number	Version Date
Version 2.0/ Initial	14 March 2020
Version 3.0	22 April 2021
Version 4.0	12 August 2021
Version 5.0	14 March 2022
Version 6.0	14 August 2022

Statement of Compliance and Signature Page

STUDY TITLE: Phase Ib First in Human Dose Escalation of GT103 in Refractory, Advanced Stage Non-Small Cell Lung Cancer (TOP 1902)

This study will be conducted in compliance with the protocol approved by the Institutional Review Boards of Duke University Health System according to Good Clinical Practice standards. No deviation from the protocol will be implemented without the prior review and approval of the IRB except where it may be necessary to eliminate an immediate hazard to a research subject. In such case, the deviation will be reported to the IRB as soon as possible. The signature below constitutes the approval (by the PI) of this protocol and the attachments, and provides the necessary assurances that this trial will be conducted according to all stipulations of the protocol, including all statements regarding confidentiality, and according to local and state legal and regulatory requirements and applicable U.S. federal regulations and ICH guidelines.

Duke Sponsor Investigator: Jeffrey Clarke, MD
Duke University Thoracic Oncology Program

____________________________________ ____________

Jeffrey Clarke, MD **Date**
Duke Sponsor-Investigator

SPONSOR CONTACT INFORMATION

Lead Principal Investigator (PI):
Dr. Jeffrey Clarke
Duke University Medical Center
DUMC 3198
25178 Morris Building
Durham, NC 27710
Phone: 919-681-9509
Fax: 919- 681-9599
Pager: 919-684-8111 and request that the doctor be paged to an outside call
Email: jeffrey.clarke@duke.edu

Background

As of July 2022, a total of 21 patients were enrolled on the study and received treatment across dose levels 1 through 4. Two patients experienced DLT: one patient at the 0.3 mg/kg dose level experiencing grade 3 acute kidney injury and one patient at 1 mg/kg dose level experiencing grade 2 colitis. **No DLT was observed at the 3 and 10 mg/kg dose levels**. Six patients (28%) experienced grade 2 or higher treatment-related adverse events. In addition to DLTs, treatment related grade 2-3 adverse events included grade 2 anorexia (n=1), grade 2 colitis (n=1), grade 2 and grade 3 lymphopenia (n=2), grade 2 creatinine increase (n=1), and grade 2 psoriasis flare (n=1).

Stable disease was demonstrated in 5/21 (24%) patients and no objective responses were seen. The median progression-free survival observed was 42 days (95% CI: 40-NE) and the median number of cycles of treatment received was 2 (range 1-8). Pharmacodynamic biomarker analysis is ongoing and with evaluation of candidate PD biomarker to guide GT103 development. An interim pharmacokinetic analysis has been performed on all treated patients on study. Based on time concentration profile and dose-normalized clearance, data suggests a non-linear drug disposition for GT103. At the highest dose level 4 (Figure 1 and 2), half-life clearance of GT103 was 161.9 hours (~6.7 days). Mean Cmax and AUC were generally similar across the dose levels.

Given the limited toxicity profile at the RP2D (10 mg/kg q3 weeks) coupled with associated pharmacokinetic data, we believe we are justified to further evaluate the MTD of GT103 by increasing dose intensity through both increasing dose frequency (from 3 to 2 weeks) and increasing dose amount.

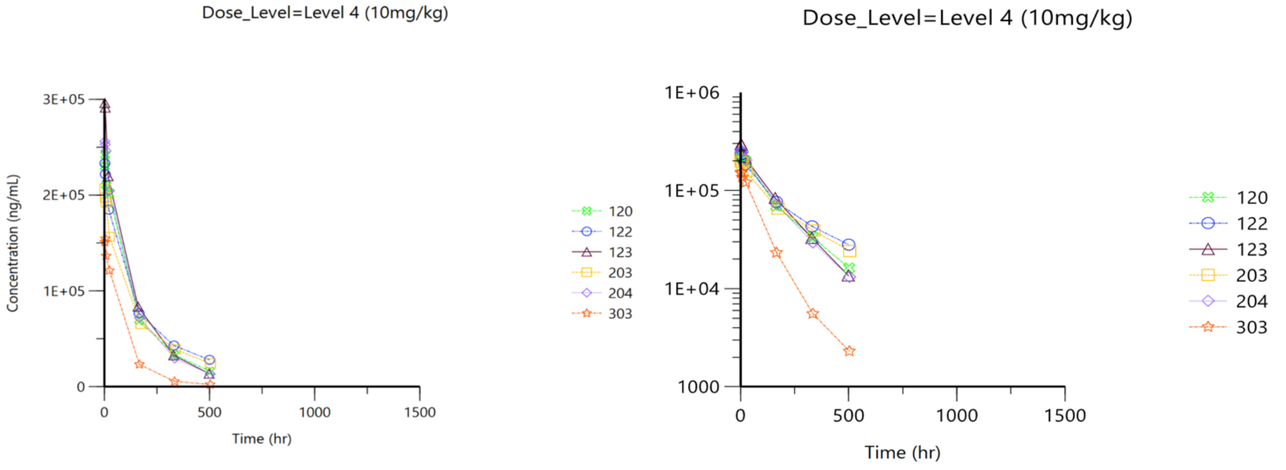

Figure 1. Time-concentration Profiles for GT104 from Dose Level 4 Using Log Scale (left) and Linear Scale (right).

Dose_Level		Cmax_D (ng/mL/mg)	AUClast_D (hr*ng/mL/mg)	Cl_obs (mL/hr)	t12	CL_dose	Vz_obs (mL)
Level 1 (0.3mg/kg)	Mean	256.186	38700.802	24.436	198.364	1.041	6804.630
Level 2 (1mg/kg)	Mean	267.097	32118.365	30.628	139.991	0.474	5645.900
Level 3 (3mg/kg)	Mean	210.124	24187.772	43.427	128.864	0.155	6899.744
Level 4 (10mg/kg)	Mean	268.715	36854.145	26.396	161.905	0.032	5565.369

Figure 2. Summary of Non-compartmental PK Analysis of Dose Levels 1 through 4.

Design

In the proposed amendment, the dose expansion (trial stage Ib) will be removed from the trial design. Two additional dose levels will be added in parallel to investigate the MTD of GT103. Dose level 4a will increase the dosing frequency of 10 mg every 2 weeks. Dose level 5 will increase the dose amount (with unchanged dose frequency) to 15 mg/ kg every 3 weeks. Patients will be assigned by the coordinating primary site (Duke University) to the dose levels in an alternating manner between cohorts and based on slot availability (first patient will be assigned to level 4a, then next patient assigned to 5, etc.). Dose levels 4a and 5 will continue with the "3+3" design enrolling 3 patients initially. If 0 or 1 DLT is observed with the first 3 patients, then an additional 3 patients will be enrolled. If 2 or more DLT is observed in the cohort at any time, the dose level will be deemed to have unacceptable toxicity. The enrollment of dose level 4a and 5 cohorts will be conducted in parallel and independent of each other.

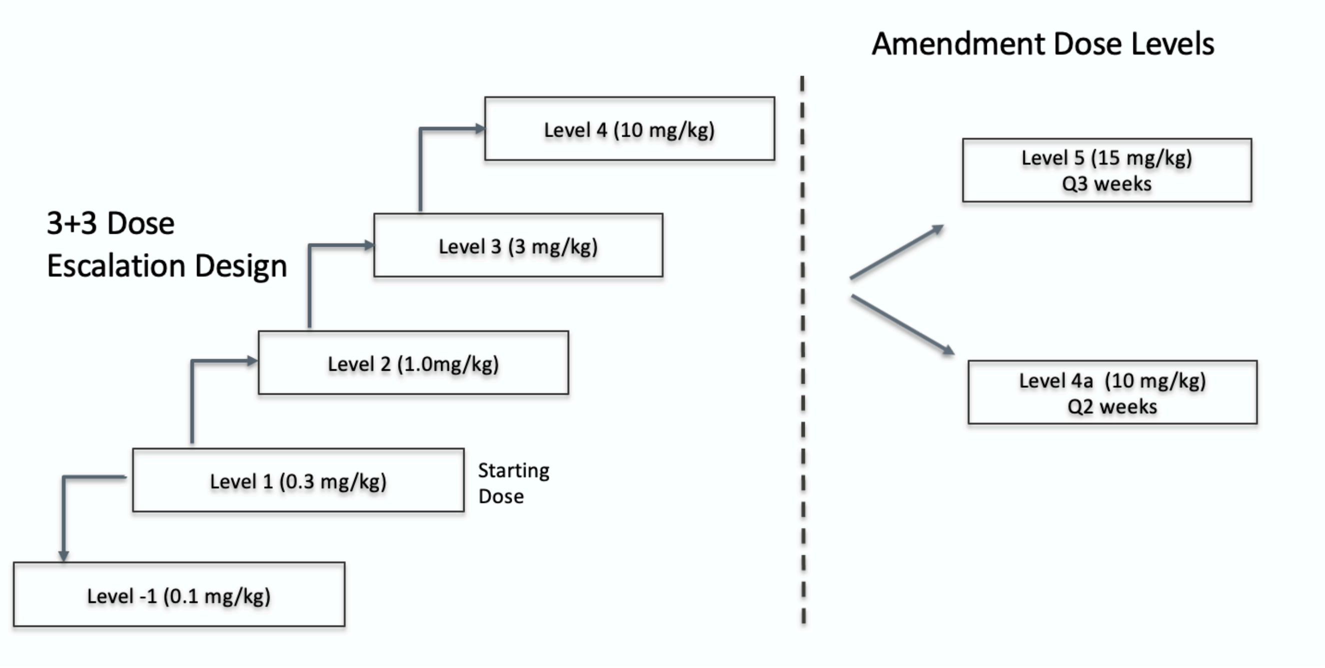

Figure 3. Amendment Dose Level Schema.

Statistical Considerations

Please see Section 9 for statistical considerations and analysis.

The DLT observation for dose level 5 will remain at 21 days 1st cycle and the DLT observation period for dose level 4a will include 2 cycles of treatment (28 days).

Dose level 4 (10 mg/kg every 3 weeks) is the current RP2D for GT103. As MTD was not established in stage 1 of the study, MTD will be investigated during dose level 4a and 5. If unacceptable toxicity is observed in dose level 5, then dose level 4a will be considered the MTD. If unacceptable toxicity is observed in dose level 4a, then dose level 5 will be considered the MTD. If no MTD is observed in dose levels 4a and 5, then the preferred dosing schedule for GT103 development will be based on collective toxicity profile, clinical efficacy, and PK/PD data between dose levels 4, 4a, and 5.

List of Amendment Alterations

The dose expansion will be removed from the trial design. Dose level 4a and 5 will be added.

The total minimum of patient enrolled between dose levels 4a and 5 is 6 patients and the maximum number is 12. Total trial enrollment (including the 21 treated patients) will be 27 to 33 patients.

PROTOCOL SYNPOSIS

Title: Phase Ib First in Human Dose Escalation of GT103 in Refractory, Advanced Stage Non-Small Cell Lung Cancer (TOP 1902)

Objectives

Primary Objectives:

1. To determine the maximum tolerated dose (MTD), if any, and the recommended phase II dose (RP2D) of GT103 for the treatment of adult patients with immuno-oncology (IO)-refractory, advanced stage (III/IV) or recurrent NSCLC whose tumors are refractory to current treatments.
2. To characterize the pharmacokinetic (PK) profile of GT103.

Secondary Objectives:

1. To describe the objective response rate to GT103.
2. To describe the progression-free survival (PFS) and overall survival (OS) of patients treated with GT103.

Exploratory Objective:

To explore serum-based and tumor-based biomarkers, as well as imaging features, that might be associated with biologic mechanisms and predict outcomes with GT103 monotherapy.

Patient Population

Subjects must have histologically and/or cytologically confirmed and radiographically measurable (by RECIST) advanced stage or recurrent NSCLC that is refractory to standard therapies including chemotherapy and immunotherapy, or for which no standard therapies exist.

Study Design

The study will include dose escalation component to determine safety and the maximum tolerated dose and RP2D for GT103. Dose escalation will begin with dose level 1 and continue as described in Table 3. Dose level 4a will include the 10 mg/kg dose given at 2 week cycle instead of 3 week cycle. Dose level 5 will include the 15 mg/kg dose given at 3 week cycle.

Number of Subjects

Total subjects enrolled will be maximum of 33 (including extra subjects enrolled to ensure that the trial obtains sufficient evaluable subjects).
The total minimum of patients enrolled between dose levels 4a and 5 is 6 patients and the maximum number is 12. Total trial enrollment (including the 21 treated patients) will be 27 to 33 patients.

Estimated Length of Study Participation

Estimated duration of study enrollment is 3 years. Subjects may receive study treatment GT103 until they experience intolerable drug-related toxicity or disease progression, or the subject wishes to withdraw from study, or the investigator recommends that the subject withdraws from the study.

Each cycle is 21 days (except dose level 4a, which is 14 days). Subjects will have weekly study visits during cycle 1. After cycle 1, subjects in dose level 5 will have study visits every 3 weeks with GT103, subjects in dose level 4a will have study visits every 2 weeks.

Subjects who discontinue study treatment for any reason other than disease progression (e.g., toxicity) should be followed every 12 weeks (+/-2 weeks) until documented disease progression or start of a new anti-cancer therapy. All subjects will be followed for survival for up to 3 years until death, loss to follow-up, or study completion.

Study Drug Regimen

In dose escalation, GT103 will be administered at a starting dose of 0.3 mg/kg intravenously every 21 days. Enrollment will proceed in a standard “3+3” design, per Table 3.

Study Assessments

Toxicity will be assessed at every visit using the NCI Common Terminology Criteria for Adverse Events (NCI-CTCAE) version 5.0. Safety assessments will be performed weekly during the first cycle of the dose escalation cohort, then every three weeks for subsequent cycles of dose escalation and every two weeks for level 4. Safety assessments will include but are not limited to vital signs, ECOG performance status, medical history, physical examination, review of concomitant medications, CBC with differential, and chemistries with liver function tests. Symptom management and supportive care will be provided as clinically indicated to ensure optimal patient care. After discontinuation of study treatment, subjects will have safety assessments 30 days (+/-14 days) after the last dose of study drug.

Tumor Assessments

Restaging scans and measurement of blood tumor markers (where applicable) will be repeated every 6 weeks. Tumor response will be assessed using RECIST version 1.1 and iRECIST. Baseline radiographic imaging will be performed with the relevant imaging to include CT scan of chest/abdomen/pelvis with or without contrast and/or MRI scan of abdomen/pelvis and all known or suspected sites of disease. Brain MRI is required for all subjects at baseline. Brain CT with contrast is allowable if there are contraindications to MRI. Restaging scans will be performed every 6 weeks after the start of study treatment for the first 48 weeks and then every 12 weeks thereafter. CT of chest and known sites of disease (e.g., abdomen with or without pelvis) is required for subjects with metastasis in those areas identified at baseline or if clinically indicated. The same method for tumor assessment should be employed at every assessment. Subjects with a history of brain metastasis are to have surveillance MRI approximately every 12 weeks or sooner if clinically indicated.

Correlative Studies

We will conduct two types of correlative studies, one for treatment efficacy (i.e., pharmacodynamic) biomarkers, and one for biomarkers that correlate with response to treatment. Additional specimens including FFPE tissue and blood will be collected and stored in a trial repository for future biomarker studies.

- **Pharmacodynamic biomarkers.** Blood will be collected for the measurement of:
    - Complement factor C3a
    - Soluble C5b-9 (sC5b-9)

Rationale: Both C3a and sC5b-9 are evidence of complement dependent cytotoxicity (CDC). They will be assayed in plasma from blood drawn at baseline and at 2, 4, 8, and 24 hours after the first dose of GT103 (all dose levels), Cycle 1 Days 8 and 15, and pre-dose Cycles 2-5, Level 4a collected cycle 1 day 8, and pre-dose cycles 2-5.

- **Predictive biomarkers.** Archived formalin-fixed paraffin-embedded (FFPE) tumor samples will be obtained from all subjects if available and assayed by immunohistochemistry for:
    - CD55
    - CD59
    - CFH

Rationale: Both CD55 and CD59 are complement regulatory proteins found on tumor cell membranes and protect tumor cells from CDC. CFH is also found on tumor cell membranes and protects tumor cells from CDC, as well as being the target of GT103.

TABLE OF CONTENTS

SPONSOR CONTACT INFORMATION 3
SUMMARY OF CHANGES- PROTOCOL V.6 4
PROTOCOL SYNPOSIS 7
TABLE OF CONTENTS 10
TABLE OF TABLES 12
TABLE OF FIGURES 12
1 INTRODUCTION 13
1.1 Background 13
1.2 Study Agent 14
1.3 Study Rationale 19
2 OBJECTIVES AND ENDPOINTS 20
3 STUDY DESIGN 21
3.1 Study Description 21
3.2 Dose Escalation with GT103 Monotherapy 22
4 SUBJECT SELECTION 24
4.1 Inclusion Criteria 24
4.2 Exclusion Criteria 25
4.3 Inclusion of Women and Minorities 26
4.4 Protocol Eligibility Waivers 26
4.5 Registration Procedure at Duke 26
4.6 Registration Procedure for Outside Sites 26
5 STUDY ASSESSMENTS 27
5.1 Screening Period 32
5.2 Treatment Period 33
5.3 Follow-up Period 34
5.4 Laboratory Assessments 35
5.5 Adverse Event Assessment 35
5.6 Tumor Assessments 35
5.6.1 RECIST version 1.1 35
5.6.2 iRECIST 36
5.6.3 Imaging Central Review: 37
5.7 Subject Withdrawal/Discontinuation 37
6 STUDY DRUGS 38
6.1 Initial and Re-Supply Orders 38
6.2 Treatment Compliance and Study Drug Accountability 38
6.3 GT103 38
6.3.1 Administration of GT103 38
6.4 Concomitant Medications/Vaccinations 39
6.4.1 Acceptable Concomitant Medications 39
6.4.2 Prohibited and/or Restricted Concomitant Medications 40
6.4.3 Palliative Radiotherapy 41
7 DOSE MODIFICATION AND TOXICITY MANAGEMENT 41
7.1 General Guidelines for Dose Modifications 41
7.2 Treatment Parameters for Day 1 of Each Cycle (Stage 1a/b) 41
7.3 Dose Modifications for GT103 42

7.3.1 General Guidelines for GT103 Dose Modifications....42
7.3.2 Hematologic Toxicity Criteria (Related to GT103)....43
7.3.3 Non-Hematologic Toxicity Criteria (Related to GT103)....43
7.4 General Supportive Care Guidance....44
7.4.1 Hematopoietic Growth Factors....44
7.4.2 Pulmonary Adverse Events....44
7.4.3 GI Adverse Events....44
7.4.4 Renal Adverse Events....45
7.4.5 Hepatic Adverse Events....45
7.4.6 Endocrinopathy Adverse Events....46
7.4.7 Skin Adverse Events....46
7.4.8 Neurologic Adverse Events....47
7.4.9 Infusion Reactions....47
7.5 Treatment Beyond Progression....48
7.6 Criteria to Resume GT103....49
8 CORRELATIVES....50
8.1 Blood Collection....50
8.2 Pharmacokinetics (PK)....50
8.3 Complement Factors (C3a and sC5b-9)....51
8.4 Blood Biomarkers for Future Research....51
8.5 Anti-Drug Antibodies....51
8.6 Tumor Tissue Biomarkers....51
8.7 Future Use of Patient Samples....52
9 STATISTICAL METHODS AND DATA ANALYSIS....52
9.1 Analysis Sets....52
9.2 Patient Demographics and Other Baseline Characteristics....52
9.3 Treatments....52
9.4 Primary Objectives....52
9.4.1 Primary Objective #1: Maximum Tolerated Dose (MTD) of GT103 Monotherapy....52
9.4.2 Primary Objective #2: Pharmacokinetic (PK) Study....53
9.5 Secondary Objectives....54
9.5.1 Secondary Objective #1: Response Rate....54
9.5.2 Secondary Objective #2: Progression-Free Survival and Overall Survival54
9.6 Exploratory Objectives....54
9.6.1 Exploratory Objective #1: Serum-Based Biomarkers....55
9.6.2 Exploratory Objective #2: Tumor-Based Biomarkers....55
9.6.3 Exploratory Objective #3: Imaging Features....56
9.7 Interim Analysis....56
9.8 Sample Size Calculation....56
10 SAFETY....56
10.1 Adverse Events....56
10.2 Serious Adverse Events....57
10.3 Events of Clinical Interest....60
10.4 Other Safety Considerations....61
10.4.1 Pregnancy and Lactation....61
10.4.2 Overdose....61

10.4.3 Potential Drug Induced Liver Injury (DILI) 61
11 ADMINISTRATIVE RESPONSIBILITIES....62
11.1 Institutional Review Board/Independent Ethics Committee....62
11.2 Protocol and Protocol Revisions....62
11.3 Protocol Deviations and Violations....62
11.4 Informed Consent....63
11.5 Source and Study Documentation....63
11.6 Case Report Forms....64
11.7 Monitoring and Audits/Inspections....65
11.8 Data Safety Monitoring Board....66
11.9 Study Closeout....67
11.9.1 Records Retention....67
12 REFERENCES....68
APPENDIX A. RECIST 1.1....70
APPENDIX B. ECOG PERFORMANCE STATUS....77
APPENDIX C. LABORATORY TESTS....78
APPENDIX D. STUDY MANUAL....79
APPENDIX E. STANDARD COCKCROFT AND GAULT FORMULA FOR CALCULATING CREATININE CLEARANCE....84

TABLE OF TABLES

Table 1: Effect of mAb7968 (GT103) on CDC of Human Tumor Cell Lines....15
Table 2: Objectives and Endpoints in Phase Ib Clinical Trial....20
Table 3: Dose Levels....22
Table 4: Dose Escalation Decision Rules....23
Table 5: Study Calendar for GT103 Therapy (Cohorts 1-4, 5)....28
Table 6: RECIST and iRECIST for Assessment of Tumor Response to TreatmentTable 36
Table 7: Hematologic CTCAE 5.0 Criteria for Dose Delay and Dose Modification.43
Table 8: Non-Hematologic Toxicity Criteria....43

TABLE OF FIGURES

Figure 1. Time-concentration Profiles for GT104 from Dose Level 4 Using Log Scale (left) and Linear Scale (right)....4
Figure 2. Summary of Non-compartmental PK Analysis of Dose Levels 1 through 4.5
Figure 3. Amendment Dose Level Schema....6
Figure 4: CFH in Exosomes and Plasma of NSCLC Patients. 16
Figure 5: Lysis of Human Exosomes After Neutralizing Complement Protective Proteins. 17

1 INTRODUCTION

1.1 Background

We studied the humoral response in a distinct group of patients with "exceptional outcomes"—those with early stage cancer who never develop metastasis—so that we might take cancer-fighting "cues" from the native immune systems of these patients. In doing so, we discovered antibodies against complement factor H (CFH) in the sera of patients with early-stage, non-metastatic non-small cell lung cancer (NSCLC). Patients with stage I NSCLC had a significantly higher incidence of anti-CFH antibody than those with late-stage NSCLC (P = 0.0051) [1]. The association of anti-CFH antibodies with this phenotype raised the possibility that they may play a role in limiting the course of the disease.

CFH is a regulatory protein that protects host cells from attack and destruction by the complement system by inhibiting the alternative pathway of complement-mediated cytotoxicity (CDC) [2, 3]. CFH acts by several mechanisms to eliminate C3b, a key protein of the complement deposition cascade, from the cell surface. Deposition of C3b initiates the formation of cell-lytic membrane attack complexes leading to cell lysis. Thus, CFH inhibition of the deposition of C3b on the cell surface protects against cell lysis. Tumor cells take advantage of the protection conferred by CFH in order to evade destruction by the complement system [4-7]. Neutralizing mouse antibodies against CFH and other complement inhibitory proteins promote activation of the complement system and increase the deposition of cell lytic complement proteins on tumor cell lines in vitro. In addition, siRNA-mediated inhibition of CFH expression in tumor cells slows xenograft growth in mice [4]. Thus, in patients, antibodies to CFH may make tumor cells susceptible to CDC. Furthermore, during complement activation, the anaphylatoxins C3a and C5a are released. C3a and C5a bind receptors on the surface of a variety of innate and adaptive immune cells and are considered to be bridges between the innate and adaptive immune systems [8]; therefore, inhibition of CFH may lead not only to tumor cell lysis but to activation of a durable adaptive immune response.

To explore the therapeutic potential of this antibody and its mechanism of action, we first affinity purified CFH antibodies from the sera of multiple patients with early stage NSCLC and mapped the antibodies' binding site to a specific 8 amino acid epitope, <u>PIDNGDIT</u>, in the short consensus repeat (SCR) 19 domain [9]. This epitope in CFH is present at the interface of CFH and C3b as seen in the published co-crystal structure [10]. Mutations in this region of CFH have been shown to eliminate binding between the two proteins. Binding of CFH antibodies to their target in this CFH-C3b interface would be expected to interfere with the interaction of the proteins. In support of this hypothesis, we have shown that affinity purified CFH autoantibodies increase the deposition of C3b on A549 lung tumor cells and cause increased tumor cell death by CDC [9]. Additionally, purified CFH autoantibodies recognize a conformationally distinct form of CFH (experimentally seen under reducing conditions) and <u>not</u> native soluble protein, suggesting a mechanism for antibody specificity for CFH on tumor cells: We hypothesize that a unique epitope is revealed in the microenvironment of a tumor. Inspection of the clinical records of NSCLC patients lends support to this hypothesis:

1. NSCLC patients with anti-CFH antibodies have no autoimmune disease.
2. NSCLC patients with anti-CFH antibodies have no evidence of renal disease, the primary effect of mutations in the CFH domain containing the anti-CFH antibody epitope [11].
3. NSCLC patients with anti-CFH antibodies show no signs of other off-target effects.

We believe that specific targeting of this epitope is key to therapeutic potential while avoiding off-target or side effects. In order to target this epitope, we developed a monoclonal antibody (mAb) to CFH with the same specificity as the endogenous CFH autoantibodies.

1.2 Study Agent

Cloning and In-Vitro Characterization of Human Anti-CFH mAbs

As a first step in the development of an anti-CFH antibody into a cancer therapeutic, we used an innovative strategy to generate human derived mAbs by affinity-selecting single CFH-specific antibody-producing B cells from the peripheral blood of NSCLC patients using a 15-mer peptide containing the epitope as bait [12]. We sequenced the immunoglobulin genes from single peripheral B cells and expressed and confirmed the specificity of 15 human anti-CFH mAbs. Sequence analysis of the variable heavy and light chain regions of these 15 mAbs revealed the presence of some clonal populations, and we ultimately identified 7 unique anti-CFH mAbs.

All 7 of the antibodies bound CFH, and all were specific for the conformationally distinct form. All the antibodies have been assessed for affinity using Biacore technology; six of the seven mAbs bind a peptide containing the PIDNGDIT epitope with Kd values of 3-8 nM with the seventh binding at much lower affinity [12]. This low affinity antibody was discarded. Alanine scanning analysis confirmed mAb binding to the PIDNGDIT epitope and revealed the most important antibody-CFH contacts within it [12].

The heavy and light chain variable domains of mAbs were initially cloned into expression cassettes as IgG1 antibodies [12]. However, sera from 24 patients with CFH autoantibodies were tested and all autoantibodies were found to be IgG3 subtype [9]. Therefore, each of the variable domain pairs were later cloned into an IgG3 framework. IgG1-mAb 7968 had similar affinity and specificity to the other high affinity mAbs but had the highest expression so was chosen for further analysis; the IgG3 version of this antibody is GT103.

Antibody Specificity *in-Vitro*

Monoclonal antibody 7968 (as IgG1, IgG3, or both) was tested in vitro for target specificity and for the ability to induce CDC, as well as for antibody dependent cellular cytotoxicity (ADCC), another possible mechanism for antibody-induced cell killing.

- mAb 7968 did not appreciably cross-react with antigens in an antinuclear antibody (ANA) panel.
- In tissue microarray studies—our own study using Pantomics array MNO661, and a GLP study conducted in conjunction with the NCI Experimental Therapeutics (NExT) program—mAb7968 does not bind to normal human tissues.

Evidence for Conformational Change in Epitope Upon Antibody Binding

To investigate the interaction of the CFH antibody with its epitope, the crystal structure of a Fab fragment of mAb 7968 in complex with a $CFH_{1110-1122}$ peptide containing the epitope sequence was solved by molecular replacement and refined to a resolution of 2.0Å[12]. Most of the CFH polypeptide backbone (residues 1112-1122) was visible in the scattering electron density, showing specific

antibody-antigen interactions in detail. Importantly, the peptide exhibited a conformation distinctly different from that observed in other structures of CFH or the SCR19 domain (where the GT103 epitope is located) [10, 13-15]. In particular, residues 1117-1120 (sequence element NGDI) near the C-terminus of the peptide adopted an α-helical conformation in the antibody-bound complex, whereas the same region exhibited a β-strand conformation in natively folded structures of CFH [10, 13-15] (see Fig. 2 of reference [12]). This was consistent with our prior observation that the CFH autoantibodies and cloned mAbs bound a conformationally distinct form of CFH.

CDC

All tumor cell lines tested consistently show increased tumor cell lysis in response to mAb7968 (GT103). The results of CDC experiments with the tested tumor cell lines are summarized in Table 1.

Table 1: Effect of mAb7968 (GT103) on CDC of Human Tumor Cell Lines

Cell Line	CFH mAb subtype	Effect on CDC relative to subtype-specific negative control*
NSCLC; H226 squamous cell carcinoma	IgG1	Dose-dependent increase from 60 µg/mL (p=0.033) to 120 µg/mL (p=0.026)
	IgG3	Increase at 150 µg/mL (p=0.04)
NSCLC; H460 large cell carcinoma	IgG1	Increase at 80 µg/mL (p=0.044)
NSCLC; A549 adenocarcinoma	IgG3	Increase at 150 µg/mL (p=0.04)
Small cell lung cancer; DMS79	IgG1	Increase at 100 µg/mL (p=0.008)
Gastric cancer; KATOIII	IgG1	Dose-dependent increase from 60 µg/mL (p=0.008) to 120 µg/mL (p=0.006)
Breast cancer; SKBR3	IgG1	Increase at 100 µg/mL (p=0.015)

* CDC experiments are carried out by incubating cells and antibodies together with normal human serum as a complement source and measuring lysis by lactate dehydrogenase release.

Additionally:

- To confirm the triggering of complement activation by the CFH mAb, we assayed products of the CDC reaction: the anaphylatoxins C3a and C5a, and the terminal membrane attack complex C5b-9. Addition of IgG1-CFH mAb to A549 or H226 lung carcinoma cells in the presence of normal human serum resulted in increased C3a release, C5a release, and C5b-9 deposition vs. levels seen using the control mAb [12].
- To investigate if any portion of the observed CDC was due to the classical complement pathway, the CDC reaction was carried out in C1q depleted serum. The binding of C1q to the

Fc of antigen-complexed antibodies initiates the classical complement pathway. C1q depleted serum caused significantly less CDC of A549 cells compared to normal human serum (p=0.01) when 300 µg/mL GT103 was added to the reaction. This suggests that CDC resulting from inactivation of CFH by the mAb is due to both classical and alternative complement pathway activation.

- To investigate the effect of the CFH mAb on normal cells, GT103 was incubated with human PBMCs. No CDC was observed.

ADCC

ADCC results when an effector cell (such as a natural killer cell) displaying an Fc receptor binds to the Fc fragment of a target bound antibody, triggering killing of the target cell. To investigate if mAb7968 (GT103) induces ADCC of tumor cells, PBMCs from laboratory personnel were mixed with A549 lung adenocarcinoma cells in the presence of the following antibodies: cetuximab (the positive control), mAb7968 (GT103), or subtype-matched control mAbs. We found:

- Cetuximab at 25 ng/ml resulted in 60% lysis of A549 cells; 250 or 1000 ng/ml caused 70% lysis.
- No detectible lysis was seen using either mAb7968 (GT103), as IgG1 or IgG3, at 50 or 500 µg/ml, or with the negative control mAbs.

Exosome Lysis

Exosomes are ~30-150 nm extracellular vesicles of endocytic origin that carry proteins and RNA from cells from which they are derived, delivering their cargo to recipient cells [16]. In lung cancer, as in other types of cancer, exosomes facilitate metastasis by promoting formation of a premetastatic niche and inducing immunosuppression [17, 18]. Exosomal PD-L1 is a powerful contributor to the immunosuppression of T cells and higher expression of PD-L1 on exosomes is associated with poorer outcomes and less responsiveness to checkpoint inhibitors [19, 20].

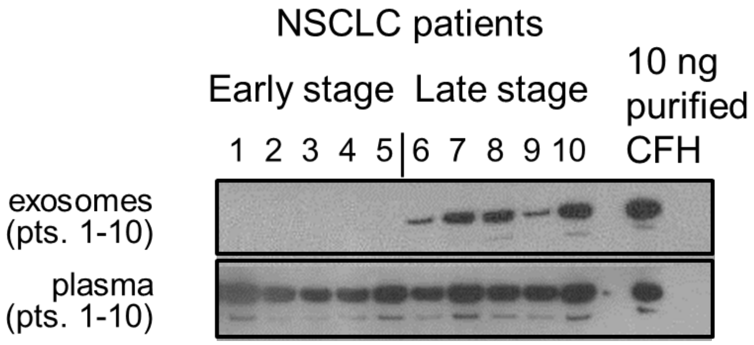

Figure 4: CFH in Exosomes and Plasma of NSCLC Patients. Exosomes were isolated from 1 ml of plasma using ultracentrifugation. Equal volumes of exosomes and plasma from each patient were subjected to western blot analysis. The blots were probed with murine GT103 followed by an anti-mouse IgG-HRP secondary reagent (to prevent background binding of a human secondary reagent to human IgG in the samples). Top blot: Exosomes from 5 early stage (1-5) and 5 late stage (6-10) patients. Bottom blot: Plasma from the same samples. Samples are a subset of 10 patients of 18 analyzed; only one early stage patient had an exosomal CFH band.

We have found that CFH is present on exosomes from murine and human lung cancer cell lines as well as murine colon and melanoma tumor cell lines and that GT103 binds to tumor cell line exosomes. In addition, exosomes isolated from the plasma of 10/11 patients with metastatic NSCLC contain CFH, whereas CFH-containing exosomes were undetectable in the plasma of 6/7 patients with early stage lung cancer (Figure 4). We propose that CFH protects exosomes from lysis, thus facilitating metastasis.

We hypothesized that neutralization of CFH with or without neutralization of another complement regulatory protein, CD59, can cause lysis of exosomes. We labelled serum exosomes from a late stage NSCLC patient with calcein and added antibodies against CFH (mAb7968), CD59 (YTH53.1, Santa Cruz), CFH+CD59, or control IgG, followed by normal human serum (NHS) as a source of complement.

We then performed flow cytometry on the exosomes. In this experiment, loss of label corresponds to exosome lysis. By measuring peak fluorescence, we found that compared to control IgG, antibodies against CFH, CD59, and CFH+CD59 increased lysis by 17%, 25%, and 42%, respectively, the combination of antibodies having an additive effect (Figure 5). These results demonstrate that exosomes are vulnerable to complement lysis when protective complement regulatory proteins are neutralized.

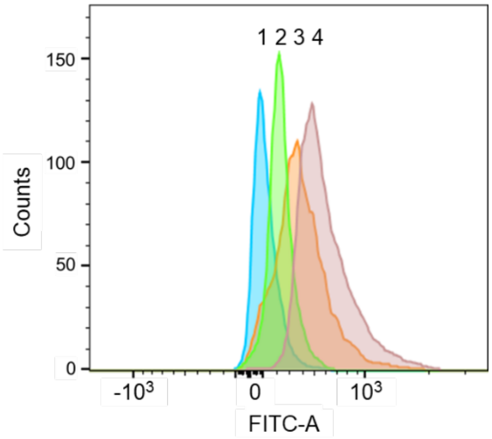

Figure 5: Lysis of Human Exosomes After Neutralizing Complement Protective Proteins. Exosomes were isolated from the serum of an NSCLC patient using the ExoQuick Ultra isolation kit (System Biosciences). Exosomes were conjugated to CD63 beads and labeled with 25 µM calcein AM. Exosomes were treated with 50 µg/ml antibodies: 1, CD59+GT103, 2, CD59, 3, GT103, or 4, IgG followed by treatment with 10% NHS for 1 hr at 37 ºC. Mixtures were analyzed by flow cytometry.

In Vivo Studies

Tumor Growth in Nude Mice

We performed tumor growth experiments in athymic nude mice with an adult patient-derived brain tumor xenograft, D-270MG [21], and intraperitoneal injection of mAbs [12]. We found:

- GT103 slowed brain tumor growth relative to a subtype-matched negative control mAb.
- At the end of the tumor growth period, mice were sacrificed, and tumors were excised. The presence of mAbs (GT103 and negative control mAb in tumor lysates was verified qualitatively.

- At 3 weeks there was evidence for mouse antibodies against human immunoglobulin and GT103 in the tumor (thus inhibiting diffusion of antibody within the tumor).
- The appearance of the kidneys in all mice receiving GT103 was normal in hematoxylin and eosin stained kidney sections examined by a pathologist.

Tumor Growth in Syngeneic Mice

Studies in a syngeneic mouse model, KLN-205 (mouse lung adenocarcinoma) in DBA/2 mice, were performed, using murine GT103; i.e., the human CDR on a mouse IgG2a backbone [12]. In the KLN-205-DBA/2 model, GT103 slowed tumor growth relative to the subclass-matched negative control mAb. An immune infiltrate was observed in the tumors of GT103-treated but not negative control mAb-treated mice. In addition, an adaptive immune response occurred in some tumor-bearing DBA/2 mice as evidenced by the production of anti-tumor cell antibodies only after mice were treated with murine GT103.

Recombinant GT103

We have produced GT103 in the form of a recombinant, fully human IgG3 mAb in CHO cells. GT103 will be injected intravenously. In vitro, GT103 has demonstrated consistent activation of the complement system leading to tumor cell death of NSCLC, small cell lung cancer, breast cancer, and gastric cancer cell lines, but not of normal PBMCs. In vivo, GT103 slowed tumor growth in both xenograft and syngeneic mouse models. Our studies to date indicate that GT103 recognizes an epitope in CFH identical to that recognized by the naturally occurring antibodies in patients. Previous studies have evaluated CFH as a therapeutic target, however, it has not been clear how to inactivate this molecule specifically in tumors without causing complement activation and resultant endothelial cell damage in the kidney, the primary site of damage when CFH activity is impaired [22, 23]. Based on *in vitro* and *in vivo* evidence, we hypothesize that GT103 is a high affinity mAb that can target the appropriate epitope in CFH and have *tumor-specific* cytotoxicity. The absence of side effects in patients with the CFH autoantibody or in mice injected with GT103 suggests that the recombinant mAb will have few, if any, off-target/side effects.

To estimate a starting dose in patients, we determined the concentration of the CFH autoantibody in patient serum by ELISA. Briefly, serum dilutions were incubated in the wells of an ELISA plate containing biotinylated antigen peptide that had been captured by NeutrAvidin. We used an anti-human IgG HRP conjugate as secondary antibody and ABTS/H_2O_2 and absorbance at 405 nm to quantify the amount of bound IgG. The concentration of CFH autoantibody was calculated from a standard curve consisting of serial dilutions of autoantibody-negative human serum spiked with GT103.

This analysis revealed that the concentration of CFH autoantibody in patient blood is in the range of 1 to 23 µg/ml (which is approximately 0.07 to 1.75 mg/kg in an average adult). Since none of these patients exhibited any evidence of ill effects due to the presence of the autoantibody, we will use this concentration range to inform our initial GT103 dosing quantities.

<u>Pre-clinical GLP Toxicology study of GT103 in Mice</u>

The objective of this study was to characterize the potential toxicity and its reversibility of a novel human monoclonal antibody (GT103) administered by intravenous (IV) injection to CD-1 mice. The GT103 monoclonal antibody is intended for use as an anticancer agent.

Animals were evaluated for clinical signs, body weight, and food consumption. Blood samples were collected for evaluation of toxicokinetics (6/sex/group), hematology (5/sex/group), clinical chemistry (5/sex/group), coagulation (10/sex/group) and cytokines (10/sex/group). A complete gross necropsy examination was performed on each animal, and tissues collected from animals evaluated for hematology and clinical chemistry were fixed for histopathological examination

GT103 was administered via intravenous injection on Days 1 and 15 at 0 (vehicle control), 3 and 100 mg/kg (n=56/sex/group). There were no clinical observations in any animal after the first dose. As expected from our previous PK studies using a fully human antibody in immune competent mice, there were deaths within 90 minutes of the second dose on Day 15. A total of 13 male mice per treatment group were administered the second dose of GT103 on Day 15 which resulted in 2 and 3 deaths at 3 and 100 mg/kg, respectively. Second dosing was discontinued in the remaining animals and data collected for evaluation after their single dose.

There were no adverse treatment-related changes in body weight, food consumption, clinical pathology, gross necropsy or microscopic evaluations in any animal receiving one or two doses, thus **we believe the immediate deaths in the animals who received the second dose on Day 15 was due to an immune reaction in the mouse due to a human protein**. In our previous repeat dose pharmacodynamic studies in mouse using a murine version of GT103, there were no unscheduled deaths at doses up to 100 mg/kg in 3 animals.

1.3 Study Rationale

Based upon the above background, we propose a first in human Phase Ib study of GT103 in refractory advanced stage (III/IV) and recurrent NSCLC. GT103 acts by enhancing lysis of tumor cells via CDC. From a safety perspective, experimental evidence shows that this antibody recognizes a conformation of the epitope that is distinct from the native state, and appears to be induced by the tumor microenvironment making the interaction tumor-specific rather than general and systemic. Finally, NSCLC patients with the native antibody do not demonstrate autoimmune or renal disease, or other signs of off-target effects.

GT103 will be dosed beginning at 0.3 mg/kg and given intravenously every 3 weeks until disease progression, unacceptable toxicity, patient decides to withdraw from the study, or general or specific changes in the patient’s condition render the patient unacceptable for further treatment in the judgement of the investigator. The starting dose is in the range of other Phase I studies employing monoclonal antibodies. In addition, this initial dose is below the highest titer seen in patients with the CFH autoantibody.

Using a standard 3+3 design, the initial dose escalation scheme for GT103 will be as follows: 0.3 mg/kg, 1mg/kg, 3 mg/kg, 10 mg/kg given intravenously every 3 weeks until disease progression, unacceptable toxicity, patient decides to withdraw from the study, or general or specific changes in the patient’s condition render the patient unacceptable for further treatment in the judgement of the

investigator. The dose escalation portion will accrue 6-24 subjects. Dose escalation is intended to estimate the MTD and/or the RP2D for further clinical investigation. Two additional dose levels will investigate the MTD (if any) of GT103 by increase dose to 15 mg/kg every 3 weeks or increasing the dose frequency 10 mg/kg every 2 weeks. Patient enrollment will be staggered during the dose escalation phase by at least 48 hours between patients. Data will be collected to determine the preferred dosing regimen for GT103 using PK profile, potential markers of biologic and clinical activity, as well as initial estimates of clinical benefit such as objective response rate, PFS, and OS.

2 OBJECTIVES AND ENDPOINTS

Primary, secondary, and exploratory endpoints are summarized in Table 2.

Table 2: Objectives and Endpoints in Phase Ib Clinical Trial

	**Objective**	**Endpoint**
**Primary**	To determine the MTD (if any) and the RP2D of GT103 for the treatment of adult patients with refractory, advanced stage NSCLC	Proportion of patients with dose-limiting toxicity (DLT) during cycle 1 within each dose level
		RP2D
**Primary**	To characterize the PK profile of GT103 when delivered to patients with advanced stage or recurrent NSCLC	Time for the concentration of GT103 to reach half of the level administered
**Secondary**	To describe the objective response rate to GT103 using RECIST 1.1 and iRECIST	Response rate at the RP2D with GT103
**Secondary**	To describe the PFS and OS of patients treated with GT103	Median PFS
		Median OS
**Exploratory**	To explore serum-based biomarkers that are associated with biologic mechanisms and clinical outcomes	Mean change in complement C3a and C5b-9 in serum between baseline and Day 21 of Cycle 1 (or Day 14 in level 4a), Cycles 2-5
**Exploratory**	To explore tumor-based biomarkers that may correlate with response to GT103	Mean level of CD55, CD59, and CFH on tumor cell membranes
**Exploratory**	To explore imaging features (sites of previous or new disease, rates of tumor growth) that may correlate with response to GT103, disease free survival, overall survival, and toxicity	Correlation between features and outcome and toxicity.

3 STUDY DESIGN

3.1 Study Description

This open-label, non-randomized Phase Ib trial is designed to assess the safety, tolerability and MTD/RP2D of GT103 as monotherapy in adult subjects with refractory, advanced stage NSCLC. The study consists of 3+3 escalation cohorts For GT103 monotherapy, subjects will be accrued (enrolled) at Duke University Medical Center and at select NCI designated cancer centers. Approximately subjects may be accrued to ensure that the trial obtains sufficient evaluable subjects. The clinical trial will include 27 to 33 enrolled patients.

- Enrolled subjects are defined as subjects who give informed consent.
- Screen failures are defined as subjects who give informed consent and do not meet eligibility criteria.
- Accrued subjects are defined as subjects who give informed consent and meet eligibility criteria. Accrued subjects may be one of the following:
  - Withdrawn: Subject accrued but later withdrawn from the study, either before or after receiving a study drug.
  - Evaluable: In the Dose Escalation cohort, subjects who were accrued, received at least 1 dose of study drug during cycle 1, and completed the first cycle of safety assessments or have dose-limiting toxicity (DLT; defined in Section 3.2) will be considered evaluable for DLT. All subjects who are accrued and receive any study treatment will be considered evaluable for toxicity and efficacy.
  - Non-evaluable for determination of DLT: In the Dose Escalation cohort, subjects who were accrued but did not complete the first cycle of safety assessments or who discontinue from the study for reasons other than a DLT (e.g., non-compliance, subject request, or symptomatic disease progression) before completing Cycle 1 of a dose level will be replaced.

The number of enrolled subjects may exceed the number of evaluable subjects for the following reasons:

- Screen failures: subjects with informed consent that do not meet inclusion/exclusion screening criteria.
- Treated subjects: subjects with informed consent that meet the inclusion/exclusion criteria and received study drug treatment but did not complete the first cycle of safety assessments or received 1 or fewer doses of study drug.

In the dose escalation phase, non-evaluable subjects will be replaced in order to complete the determination of DLT.

Monotherapy Dose Escalation: The first portion will be a dose escalation component to determine safety and the maximum tolerated dose and RP2D for GT103. Dose escalation will begin with a cohort of patients treated at dose level 1 with future patients treated at higher or lower dose levels (Table 3) according to rules provided in Section 3.2; dose level -1 is a de-escalation dose to be implemented as necessary.

The study initially focused on dose level 1-4. After completion of patient cohorts treated at dose levels 1-4, the protocol has been amended to treat patients at dose level 4a and 5.

Table 3: Dose Levels

Dose Level	GT103 Intravenously (Q3 weeks)
-1	0.1 mg/kg
**Starting Dose 1**	**0.3 mg/kg**
2	1 mg/kg
3	3mg/kg
4	10 mg/kg
**4a**	**10 mg/kg Q2 weeks**
**5**	**15 mg/kg Q3 weeks**

Subjects will be treated on a 21-day cycle (dose level 4a will have 14 day cycle) after satisfying eligibility and screening criteria. Toxicity will be assessed every visit and as clinically indicated. Response will be assessed every 6 weeks and as clinically indicated. After all subjects in a given dose cohort have completed one cycle of treatment (or 2 cycles for subjects in cohort 4a), safety data will be collected and reviewed by the data safety monitoring board (DSMB) who are otherwise unaffiliated with this study, for determination of the next dose cohort. The DSMB will be composed of 2 thoracic medical oncologists and a biostatistician.

3.2 Dose Escalation with GT103 Monotherapy

Subjects will be enrolled to assess the MTD of GT103 monotherapy. Adverse events (AEs) will be graded using the NCI-CTCAE v. 5.0. The following AEs will be considered dose limiting toxicity (DLT) if occurring during the first cycle of treatment and deemed to be possibly, probably, or definitely related to study treatment as determined by the investigator:

- Hematologic toxicity: Any grade 4 neutropenia, thrombocytopenia or anemia or grade ≥ 3 neutropenia or thrombocytopenia lasting over 7 days
- Any grade 3 thrombocytopenia associated with bleeding
- Febrile neutropenia (grade 3/4)
- Nausea/Vomiting or Diarrhea ≥ grade 3 **and** lasting ≥ 3 days despite adequate supportive measures
- Grade ≥ 3 ALT or AST elevation
- Other non-hematologic toxicity ≥ grade 3 excluding alopecia, anorexia, fatigue, lab abnormalities deemed not clinically significant by the Investigator

- Rare, idiosyncratic reactions to the study drug (grade 3/4).
- Fatigue (lasting longer than 7 days) and hypertension will be considered as DLT only if they ≥3 or are considered unmanageable.
- Treatment delay of > 7 days in cycle 1 related to toxicity. (Note: If there are treatment delays, the study calendar will move).
- Any treatment-related death or treatment-related hospitalization
- Grade 4 anaphylaxis, hypotension, hypoxia or acute respiratory distress

Management of AEs and dose modifications associated with AEs are outlined in Section 7. Dose escalation will proceed in stage 1 within each cohort according to the following scheme:

Table 4: Dose Escalation Decision Rules

Number of Subjects with DLT at a Given Dose Level	Escalation Decision Rule
0 out of 3	Enter 3 subjects at the next dose level. (Cohort 4a/5 enroll 3 subjects at current dose level).
1 out of 3	Enter 3 more subjects at this dose level.
≤1 out of 6	(a) Proceed to the next dose level<br>**OR**<br>(b) This will be the MTD/RP2D if at highest dose level or 6 subjects already treated at the next dose level.
≥2 out of 3-6	Stop dose escalation. Enter 3 additional subjects at the next lowest dose level (if only 3 subjects already treated at that level).

- Subjects will be accrued at the starting dose level. If no DLTs are seen, 3 subjects will be enrolled in the next dose level.
- Patient enrollment will be staggered during the dose escalation phase by at least 48hrs between patients
- All subjects will be monitored for at least three weekly visits (one full cycle) before advancement to the next dose level.
- If one of three subjects has DLT at a given dose level, then an additional three subjects will be enrolled at that dose level.
- If 1/6 subjects experiences DLT at a given dose level, then escalation will continue to the next dose level.
- If ≥ 2 out of 3-6 subjects have DLT at any dose level, then that dose level will be considered to have unacceptable toxicity, and the next lower dose level will be expanded to 6 subjects. In this case, the next lower dose level will be declared as the MTD provided ≤ 1/6 subjects have DLT (otherwise, dose will be further de-escalated).
    - In cases where the only toxicity seen at this lower dose level is Grade ≤ 2, the protocol may be amended to allow investigation of an intermediate dose level.
- If no unacceptable toxicity is seen at the highest dose level, then the highest dosing regimen will be considered the RP2D.
- A minimum of 6 subjects will be enrolled at MTD to better define tolerability and safety before proceeding to the expanded cohort.

As previously noted, after the escalation of doses through dose level 4 was completed, the protocol was amended to add dose level 4a and 5. With this amendment, patients will be assigned to the dose levels in an alternating manner (4a or 5) and based on slot availability. Dose levels 4a and 5 will continue with the "3+3" design enrolling 3 patients initially. If 0 or 1 DLT is observed with the first 3 patients, then an additional 3 patients will be enrolled. If 2 or more DLT is observed in the cohort at any time, the dose level will be deemed to have unacceptable toxicity. The enrollment of dose level 4a and 5 cohorts will be conducted in parallel and independent of each other.

4 SUBJECT SELECTION

4.1 Inclusion Criteria

1. Histologically and/or cytologically confirmed advanced stage III, IV or recurrent NSCLC whose tumors have progressed on prior therapy.
2. Prior Therapy:
    - Patients must have received immunotherapy (anti-PD-1/PD-Ll) and a platinum-based chemotherapy either concomitantly or sequentially. There is no defined window of time since receiving immunotherapy. Immunotherapy does not have to be the immediate treatment prior to this protocol therapy.
    - Patients with EGFR, ALK, or ROS1 alterations must have received at least one prior TKI and prior chemotherapy (at least one platinum doublet regimen).
    - Stage III patients:
        - If previously treated with immunotherapy participants with investigator-assessed radiographic disease progression or recurrence in less than 6 months after the last dose of immunotherapy in Stage III B/C disease post concurrent chemoradiotherapy followed by immunotherapy are eligible. Radiographic progression must be documented via pretreatment scan as compared to the prior therapy baseline scan in order for the participant to be eligible.
3. Disease must be measurable by RECIST 1.1 criteria (see Appendix A). Tumor lesions in a previously irradiated area are considered measurable **IF** progression has been demonstrated in such lesions after radiation.
4. Age ≥ 18 years
5. ECOG Performance Status 0 or 1 (see Appendix B)
6. Adequate bone marrow function as shown by:
    - ANC ≥ 1.5 x 10^9
    - Platelets ≥ 100 x 10^9/L
    - Hemoglobin ≥ 9 g/dL; Erythropoietin and transfusion support is permitted. If patient is receiving supportive care, hemoglobin must be stable above or equal to 9 g/dL for at least 2 weeks prior to day 1 of study drug without blood transfusion to maintain hemoglobin level.
7. Adequate liver function as shown by:
    - serum bilirubin ≤ 1.5x ULN
    - ALT and AST ≤ 2.5x ULN ; ≤ 5 x ULN with liver metastasis
8. Adequate renal function: defined as creatinine clearance (estimated) ≥ 50 cc/min by Cockroft Gault or 24-hour urine (see Appendix E).
9. Women of childbearing potential (WOCBP) must have a negative serum pregnancy test at screening and prior to treatment on Cycle 1 Day 1; both men and women must be willing to use

two medically accepted methods of contraception, one of them being a barrier method during the study and for 6 months after last study drug administration. See Section 6.4.1.1 for a list of acceptable methods of contraception.

10. Signed informed consent
11. Willing and able to comply with clinic visits and study-related procedures and requirements.

4.2 Exclusion Criteria

1. Patients currently receiving anticancer therapies or who have received anticancer therapies within 2 weeks from day 1 of study drug (including investigational agents, chemotherapy, and antibody-based therapy).
2. Patients currently receiving extracranial palliative radiation within 2 weeks from day 1 of study drug.
3. Patients who:
    a. Have had a major surgery or significant traumatic injury within 4 weeks from day 1 of study drug,
    b. Have not recovered from the side effects of any major surgery (defined as requiring general anesthesia), or
    c. Are anticipated to require major surgery during the course of the study.
4. Intolerance to PD-1/PD-L1 axis drug(s), or any other antibody or drug specifically targeting T-cell co-stimulation or immune checkpoint pathways, including prior therapy with anti-tumor vaccines or other immune-stimulatory anti-tumor agents. (Intolerance: Toxicity that warrants no subsequent/further PD-1/PD-L1 therapy).
5. Patients receiving chronic, systemic treatment with corticosteroids or another immunosuppressive agent with the following exceptions:
    a. Intermittent steroids (not to exceed prednisone 10 mg every day or equivalent dosing) may be used on an as-needed basis (e.g., treatment for chemotherapy-related nausea, anorexia and fatigue.)
    b. Patients on physiologic replacement doses of steroids due to adrenal insufficiency for any reason may remain on these medications.
    c. Topical, inhaled or intra-articular corticosteroids
6. Symptomatic brain or leptomeningeal metastases, including patients who continue to require glucocorticoids and/or antiseizure therapy for brain or leptomeningeal metastases.
    a. **Treated**, asymptomatic metastases are permitted provided the patient has completed radiation at least 2 weeks prior to day 1 and has been off steroids for at least 2 weeks prior to day 1 of study drug.
    b. **Untreated** brain metastases are permitted if asymptomatic, do not require immediate treatment, steroids and/or antiseizure medications, and are ≤ 1cm.
7. Presence of poorly controlled atrial fibrillation (ventricular heart rate >100 bpm) by EKG.
8. Previous history of CVA, TIA, angina pectoris, acute MI or history of recent re-perfusion procedures (e.g., PTCA), pulmonary embolus or untreated deep vein thrombosis within 6 months from day 1 of study drug. NOTE: Subjects with recent deep vein thrombosis and/or pulmonary embolus who have been therapeutically anti-coagulated for at least 6 weeks are eligible.
9. Patients who have any severe and/or uncontrolled medical conditions or other conditions that could affect their participation in the study. Examples include but are not limited to:
    a. Active (acute or chronic) infections requiring treatment with antibiotics.

b. Liver disease such as cirrhosis, chronic active or persistent hepatitis B or C or HIV.
c. Age related macular degeneration, atypical hemolytic uremia syndrome, glomerulonephritis, or known autoimmune diseases.
d. A concurrent diagnosis of a separate malignancy is allowed if clinically stable and does not require tumor-directed therapy

10. History of interstitial pneumonitis of autoimmune etiology (including immune checkpoint pneumonitis) which has been symptomatic and/or required treatment. History of radiation pneumonitis is allowed if the patient has recovered and does not currently require steroid therapy.
11. Significant vascular disease (e.g., aortic aneurysm requiring surgical repair or recent peripheral arterial thrombosis) within 6 months prior to day 1 of study drug
12. Known autoimmune conditions requiring systemic immune suppressive therapy other than prednisone less than or equal to 10 mg.
13. Known history of HIV seropositivity or known acquired immunodeficiency syndrome (AIDS).
14. Female patients who are pregnant or breast feeding, or adults of reproductive potential who are not using effective birth control methods. See Section 6.4.1.1 for a complete list of medically acceptable birth control methods.
15. Corrected QTc interval > 480 msec. If QTc interval is > 480 msec, then 2 additional ECGs should be obtained over a brief period (e.g., within 15-20 minutes) to confirm the abnormality. The average QTc interval will be determined from the 3 ECG tracings by manual evaluation and will be used to determine if the subject will be excluded from the study. The same method of QTc determination must be used throughout the subject's participation in the trial.
16. Patients unwilling to or unable to comply with the protocol.

4.3 Inclusion of Women and Minorities

Men and women of all races and ethnic groups are eligible for this trial.

4.4 Protocol Eligibility Waivers

No waivers of inclusion or exclusion criteria will be granted. All prospective patients must meet all entry criteria. If there are any questions regarding the interpretation of a criterion for a potential patient, contact the principal investigator to discuss.

4.5 Registration Procedure at Duke

Patient registration for all patients signing informed consent will be completed through the Duke Cancer Institute (DCI) Clinical Research Unit (CRU) into EPIC and ONCORE systems within 1 business day of obtaining consent. Patients will be enrolled only after all pre-treatment evaluations are completed and all eligibility criteria are met.

4.6 Registration Procedure for Outside Sites

All patients signing informed consent at outside sites must be registered with Duke (including screen failures - indicate screen failure status on last page of eligibility checklist). The following documents are to be completed by the investigator and/or designee and scanned/emailed to the Duke MultiSite Trials Service Center (MiSTiC) (DCI-MultiSiteStudies@duke.edu):

- TOP 1902 Eligibility Checklist with supporting source documents
- Signed Informed Consent

5 STUDY ASSESSMENTS

Table 5 depicts the study calendar for the GT103 clinical trial.

For all study assessments, every effort to should be made to comply with the study schedule. However, it is known that scheduling issues may occur. For all scheduling issues other than management of treatment toxicities, inter-current illness, etc., a 3-day window for treatment dates is allowed for study assessments in cycle 1, and a 7-day window is allowed in cycle ≥ 2. Infusions may be delayed up to 3 days in cycle 1, and up to 7 days in cycle 2 and beyond (but may not be given early). For scheduling changes that occur outside of these windows, particularly those due to toxicity, the Principal Investigator should be notified and provide prior approval in writing and protocol deviations filed with local IRB. Protocol waivers or exemptions are not allowed with the exception of immediate safety concerns, these should be discussed immediately with the PI upon occurrence or awareness to determine if the subject should continue or discontinue study treatment.

Table 5: Study Calendar for GT103 Therapy (Cohorts 1-4, 5)

Study Procedures	Screening Period[1]		Treatment Period[15](cycle =3 weeks)						Follow-up Period	
	Days -28 to -1[1]	Cycle 1 Day 1	Cycle 1 Day 2	Cycle 1 Day 8	Cycle 1 Day 15	Cycle 2 Day 1	Restaging (Every 6 weeks)	Subsequent Cycles Day 1	30 Day Follow-up[11]	Long Term
Informed Consent	X									
Demographics	X									
Medical and Cancer History	X									
Concomitant Medications	X[2]	-------------------------------------throughout study------------------------------------								
Physical Examination	X[2]	X		X	X	X		X	X	
Height	X									
Vital Signs and Weight	X[2]	X[16]		X	X	X[16]		X[16]	X	
ECOG Performance Status	X[2]	X		X	X	X		X	X	
CBC with Differential	X[2]	X[3]		X	X	X		X	X	
Chemistries[5]	X[2]	X[3]		X	X	X		X	X	
Thyroid Function[6]	X[2]						X			
Pregnancy Test[4]	X[2]	X				X		X		
Urinalysis	X[2]	X[3]							X	
ECG (12 lead)	X[2,13]	X[2,13]				X[13]		X[13]		
GT103 Administration[7]		X				X		X		
Adverse Event Assessment	X[2]	-------------------------------------throughout study------------------------------------								
GT103 PKs and complement[12]		X[12]	X[12]	X[12]	X[12]	X[12]		X[12]		
Tumor Assessment[8]	X						X		X[8]	X[8]
Archived Tumor Tissue[9]	X									
Future Research Blood*[14]		X[14]		X[14]		X[14]		X[14]	X[14]	X[14]
Survival										X[10]
Anti-drug antibodies (ADA)		X[17]				X[17]		X[17]		
HIV 1 & 2	X[2]									
HBsAG	X[2]									
HCV Antibody	X[2]									

1. Screening procedures must be performed within 28 days prior to day 1 of study drug (unless otherwise specified in footnotes). Informed consent must be obtained before initiation of any screening procedure that is performed. Evaluations performed as part of routine care before informed consent may be considered as screening evaluations. Baseline and Cycle 1 Day 1 procedures may be completed on the same day, however, screening assessment(s) to confirm eligibility MUST have already been determined.
2. The following screening procedures are to be performed within 14 days prior to day 1 of study drug: Physical exam, vital signs, weight, ECOG performance, labs (including serum pregnancy testing if applicable and urinalysis), 12 lead ECG, AE assessment (review of baseline symptoms) and concomitant medication review. **12 lead ECG must be repeated cycle 1 day 1.**
3. Screening labs done within 7 days prior to treatment initiation do not have to be repeated on Cycle 1 Day 1. If any screening labs are repeated on Cycle 1 Day 1, eligibility parameters must be re-confirmed.
4. Serum B-HCG for WOCBP potential only, per institutional policy.
5. Serum chemistry will include albumin, alkaline phosphatase, ALT, AST, total bilirubin, bicarbonate, BUN, calcium, chloride, creatinine, glucose, phosphorus, potassium, total protein, sodium
6. Includes TSH, T3 (total) and Free T4. To be drawn every 6 weeks regardless of treatment cycle.
7. GT103 administration q 21 days until disease progression, unacceptable toxicity, patient decides to withdraw from the study, or general or specific changes in the patient's condition render the patient unacceptable for further treatment in the judgement of the investigator.
8. Tumor Imaging: Baseline imaging will be performed with the relevant images to include CT scan (or MRI) of Chest/abdomen/pelvis with or without contrast and all known or suspected sites of disease. Brain MRI is required for all subjects at baseline. Brain CT with contrast is allowable if there are contraindications to MRI. For patients with treated brain metastases, if brain MRI falls outside of the screening window, a repeat brain MRI is not needed unless greater than 4 weeks from the completion date of radiation therapy.
Restaging scans CT of chest and known sites of disease (e.g., abdomen with or without pelvis) required for subjects with metastasis in those areas identified at baseline or if clinically indicated, will be performed every 6 weeks after the start of study treatment for the first 48 weeks, then every 12 weeks thereafter. The same method for tumor assessment is to be employed at every assessment. Subjects with a history of brain metastasis to have surveillance MRI/CT approximately every 12 weeks of sooner if clinically indicated. Subjects who discontinue study treatment for any reason (e.g., toxicity) other than disease progression will have disease status followed every 12 weeks +/- 2 weeks until disease progression, or start of new anti-cancer regimen.
9. Archived tumor specimen will be collected if available
10. Subjects will be followed for survival every 12 weeks (+/- 14 days) via personal interview or review of medical or public records for up to 3 years or study closure, whichever comes first.
11. Follow up visit should occur 30 days (+/-14 days) after the last dose of study drug.
12. Collect 2 purple top tubes pre-dose on Cycle 1 Day 1, and at 2, 4, 8, and 24 hours after the first dose of GT103, and on Cycle 1 Day 8 and Day 15, and pre-dose on Day 1 of Cycles 2-5.
13. Corrected QTc interval must be <480 msec. If QTc interval is >480 msec, then two additional 12 lead ECGs should be obtained over a brief period of time (e.g., within 15-20 minutes) to confirm the abnormality. The average QTc interval will be determined from the triplicate ECGs by manual evaluation and will be used to determine if the subject will be excluded from the study or would require dose hold for grade 2 QTc>480 in CXD1s.
14. Collect 1 purple top tube pre-dose on Cycle 1 Day 1, on Cycle 1 Day 8, and pre-dose on Day 1 of subsequent cycles, the 30 day follow-up and at time of progression. *Future Research Blood: For those subjects that consent to future research blood.
15. A 3-day window for treatment dates is allowed for study assessments in cycle 1, and a 7-day window is allowed in cycle ≥ 2. Infusions may be delayed up to 3 day in cycle 1, and up to 7 days in cycle 2 and beyond (but may not be given early).
16. For the first two infusions at each dose level, the patient's vital signs (heart rate, respiratory rate, blood pressure and temperature) will be monitored at the following time points: within 60 minutes before, during (every 15 [±5) minutes), and 30 (±5) minutes, and 2 hour (± 15 minutes) 4 hours (±15 minutes) , and 6 hours (±15 minutes) after the infusion. For subsequent infusions (if first two infusions delivered without infusion-associated AEs), vital signs to be collected within 60 minutes of infusion, during the infusion if clinically indicated and 1 hour (±10 minutes) after end of infusion.
17. Collect one purple top tube for ADA analysis. Collect pre-dose day 1 cycles 1-5.

Table 6: Study Calendar for GT-103 Therapy (Cohort 4a)

**Study Procedures**	**Screening Period[1]**		**Treatment Period[15](Cycle = 2 weeks)**						**Follow-up Period**	
	Days -28 to -1[1]	Cycle 1 Day 1	Cycle 1 Day 2	Cycle 1 Day 8		Cycle 2 Day 1	Restaging (Every 6 weeks)	Subsequent Cycles Day 1	30 Day Follow-up[11]	Long Term
Informed Consent	X									
Demographics	X									
Medical and Cancer History	X									
Concomitant Medications	X[2]	-------------------------------------throughout study-------------------------------------								
Physical Examination	X[2]	X		X		X		X	X	
Height	X									
Vital Signs and Weight	X[2]	X[16]		X		X[16]		X[16]	X	
ECOG Performance Status	X[2]	X		X		X		X	X	
CBC with Differential	X[2]	X[3]		X		X		X	X	
Chemistries[5]	X[2]	X[3]		X		X		X	X	
Thyroid Function[6]	X[2]						X			
Pregnancy Test[4]	X[2]	X				X		X		
Urinalysis	X[2]	X[3]							X	
ECG (12 lead)	X[2,13]	X[2,13]				X[13]		X[13]		
GT103 Administration[7]		X				X		X		
Adverse Event Assessment	X[2]	-------------------------------------throughout study-------------------------------------								
GT103 PKs and complement[12]		X[12]	X[12]	X[12]		X[12]		X[12]		
Tumor Assessment[8]	X						X		X[8]	X[8]
Archived Tumor Tissue[9]	X									
Future Research Blood*[14]		X[14]		X[14]		X[14]		X[14]	X[14]	X[14]
Survival										X[10]
Anti-drug antibodies (ADA)		X[17]				X[17]		X[17]		
HIV 1 & 2	X[2]									
HBsAG	X[2]									
HCV Antibody	X[2]									

1. Screening procedures must be performed within 28 days prior to day 1 of study drug (unless otherwise specified in footnotes). Informed consent must be obtained before initiation of any screening procedure that is performed. Evaluations performed as part of routine care before informed consent may be considered as screening evaluations. Baseline and Cycle 1 Day 1 procedures may be completed on the same day, however, screening assessment(s) to confirm eligibility MUST have already been determined.
2. The following screening procedures are to be performed within 14 days prior to day 1 of study drug: Physical exam, vital signs, weight, ECOG performance, labs (including serum pregnancy testing if applicable and urinalysis), 12 lead ECG, AE assessment (review of baseline symptoms) and concomitant medication review. **12 lead ECG must be repeated cycle 1 day 1.**
3. Screening labs done within 7 days prior to treatment initiation do not have to be repeated on Cycle 1 Day 1. If any screening labs are repeated on Cycle 1 Day 1, eligibility parameters must be re-confirmed.
4. Serum B-HCG for WOCBP potential only, per institutional policy.
5. Serum chemistry will include albumin, alkaline phosphatase, ALT, AST, total bilirubin, bicarbonate, BUN, calcium, chloride, creatinine, glucose, phosphorus, potassium, total protein, sodium
6. Includes TSH, T3 (total) and Free T4. To be drawn every 6 weeks regardless of treatment cycle.
7. GT103 administration q 14 days until disease progression, unacceptable toxicity, patient decides to withdraw from the study, or general or specific changes in the patient's condition render the patient unacceptable for further treatment in the judgement of the investigator.
8. Tumor Imaging: Baseline imaging will be performed with the relevant images to include CT scan (or MRI) of Chest/abdomen/pelvis with or without contrast and all known or suspected sites of disease. Brain MRI is required for all subjects at baseline. Brain CT with contrast is allowable if there are contraindications to MRI. For patients with treated brain metastases, if brain MRI falls outside of the screening window, a repeat brain MRI is not needed unless greater than 4 weeks from the completion date of radiation therapy.
Restaging scans CT of chest and known sites of disease (e.g., abdomen with or without pelvis) required for subjects with metastasis in those areas identified at baseline or if clinically indicated, will be performed every 6 weeks after the start of study treatment for the first 48 weeks, then every 12 weeks thereafter. The same method for tumor assessment is to be employed at every assessment. Subjects with a history of brain metastasis to have surveillance MRI/CT approximately every 12 weeks of sooner if clinically indicated. Subjects who discontinue study treatment for any reason (e.g., toxicity) other than disease progression will have disease status followed every 12 weeks +/- 2 weeks until disease progression, or start of new anti-cancer regimen.
9. Archived tumor specimen will be collected if available
10. Subjects will be followed for survival every 12 weeks (+/- 14 days) via personal interview or review of medical or public records for up to 3 years or study closure, whichever comes first.
11. Follow up visit should occur 30 days (+/-14 days) after the last dose of study drug.
12. Collect 2 purple top tubes pre-dose on Cycle 1 Day 1, and at 2, 4, 8, and 24 hours after the first dose of GT103, and on Cycle 1 Day 8, and pre-dose on Day 1 of Cycles 2-5.
13. Corrected QTc interval must be <480 msec. If QTc interval is >480 msec, then two additional 12 lead ECGs should be obtained over a brief period of time (e.g., within 15-20 minutes) to confirm the abnormality. The average QTc interval will be determined from the triplicate ECGs by manual evaluation and will be used to determine if the subject will be excluded from the study or would require dose hold for grade 2 QTc>480 in CXD1s.
14. Collect 1 purple top tube pre-dose on Cycle 1 Day 1, on Cycle 1 Day 8, and pre-dose on Day 1 of subsequent cycles, the 30 day follow-up and at time of progression. *Future Research Blood: For those subjects that consent to future research blood.
15. A 3-day window for treatment dates is allowed for study assessments in cycle 1, and a 7-day window is allowed in cycle ≥ 2. Infusions may be delayed up to 3 day in cycle 1, and up to 7 days in cycle 2 and beyond (but may not be given early).
16. For the first two infusions at each dose level, the patient's vital signs (heart rate, respiratory rate, blood pressure and temperature) will be monitored at the following time points: within 60 minutes before, during (every 15 [±5) minutes), and 30 (±5) minutes, and 2 hour (± 15 minutes) 4 hours (±15 minutes) , and 6 hours (±15 minutes) after the infusion. For subsequent infusions (if first two infusions delivered without infusion-associated AEs), vital signs to be collected within 60 minutes of infusion, during the infusion if clinically indicated and 1 hour (±10 minutes) after end of infusion.
17. Collect one purple top tube for ADA analysis. Collect pre-dose day 1 cycles 1-5.

5.1 Screening Period

The study calendar summarizes the trial procedures to be performed at each visit. Individual trial procedures are described in detail below.

For all study assessments, every effort is to be made to comply with the study schedule. However, it is known that scheduling issues may occur. For all scheduling issues other than management of treatment toxicities, intercurrent illness, etc., a 3-day window for treatment dates is allowed for study assessments in cycle 1, and a 7-day window is allowed in cycle ≥ 2. Infusions may be delayed up to 3 days in cycle 1, and up to 7 days in cycle 2 and beyond (**but may not be given early**). For scheduling changes that occur outside of these windows, particularly those due to toxicity, the Principal Investigator should be notified and provide prior approval in writing.

Furthermore, additional evaluations/testing may be deemed necessary by the sponsor and/or GRID for reasons related to subject safety.

During the Screening Period, subjects are consented and screened for the study. Informed consent must be obtained before initiation of any screening procedure that is performed solely for the purpose of determining eligibility for this study. Evaluations performed as part of routine care before informed consent can be considered as screening evaluations if done within the defined screening period, and if permitted by the local Institutional Review Board (IRB)/Independent Ethics Committee (IEC) policies. Study eligibility is based on meeting all of the inclusion criteria and none of the exclusion criteria (refer to Section 4) before the first dose of study drug on Cycle 1 Day 1.

The following study procedures must be done within 28 days prior to Cycle 1 Day 1 (unless otherwise specified). Baseline and Cycle 1 Day 1 procedures may be completed on the same day, however, screening assessment(s) to confirm eligibility MUST have already been determined.

- Demographics
- Medical and cancer history
- Height
- Tumor assessment (CT and/or MRI scans) of Chest, Abdomen and Pelvis and all known or suspected sites of disease. MRI of brain (preferred) or CT Brain with contrast required for all subjects
    - For patients with treated brain metastases, if brain MRI falls outside of the screening window, a repeat brain MRI is not needed unless greater than 4 weeks from the completion date of radiation therapy
- Procurement of archived tumor tissue collected as part of standard of care may be collected outside of baseline.

The following study procedures must be done within 14 days prior to Cycle 1 Day 1:

- Physical examination which includes vital signs and weight
- ECOG performance status
- CBC with differential
- Chemistries including liver function tests (LFTs)
- Thyroid function tests
- Serum pregnancy test (only for women of childbearing potential) per institutional policy.
- Urinalysis

- ECG
- AE assessment (review of baseline symptoms)
- Concomitant medications
- HIV 1 &2, HBsAG and HCV Antibody

Subject eligibility is determined using lab results obtained prior to dosing on Cycle 1 Day 1. Any laboratory assessments repeated on Cycle 1 Day 1 must meet eligibility requirements. The Screening Period ends upon receipt of the first dose of study drug or final determination that the subject is ineligible for the study.

5.2 Treatment Period

During the Treatment Period, subjects will receive GT103 on day 1 of each cycle. Patient enrollment will be staggered during the dose escalation phase by at least 48 hours between patients. Subjects will continue to receive GT103 until disease progression, unacceptable toxicity, patient decides to withdraw from the study, or general or specific changes in the patient's condition render the patient unacceptable for further treatment in the judgement of the investigator as described in Section 5.7. Toxicity-related dose modifications of GT103 may occur during the Treatment Period for cycles 2 and beyond in stage 1, and for any cycle in stage 2. Dose modification guidelines are described in Section 7.1. If clinically indicated, additional visits and/or safety assessments may be warranted.

The following study procedures must be completed on Day 1 of each cycle (unless otherwise specified). A 3-day window for treatment dates is allowed for study assessments in cycle 1, and a 7-day window is allowed in cycle ≥ 2. Infusions may be delayed up to 3 days in cycle 1, and up to 7 days in cycle 2 and beyond (but may not be given early).

- Physical examination*
- Vital signs and weight*
- Concomitant medications*
- ECOG performance status*
- AE assessment*
- CBC with differential* (does not need to be repeated on Cycle 1 Day 1 if performed within 7 days prior)
- Chemistries including LFTs* (does not need to be repeated on Cycle 1 Day 1 if performed within 7 days prior)
- Urinalysis (does not need to be repeated on Cycle 1 Day 1 if performed within 7 days of study drug initiation), only required cycle 1
- Serum pregnancy test (only for women of childbearing potential)
- 12 lead EKG
- GT103 administration
- GT103 PK, complement blood: 2 purple top tubes immediately pre-dose on Cycle 1 Day 1, and at 2, 4, 8, and 24 hours after the first dose of GT103; on Cycle 1 Day 8 and Day 15 (Day 15 excluded for subjects in cohort 4a); and pre-dose on Day 1 of Cycles 2-5.
- ADA analysis: 1 purple top tube pre-dose day 1 cycles 1-5.
- Future research bloods (for those subjects that sign consent): 1 purple top tube collected pre-dose Cycle 1 Day 1; on Cycle 1 Day 8; pre-dose on Day 1 of subsequent cycles; at the 30 day follow-up; and at the time of progression.

The following study procedure is to be completed every 6 weeks regardless of treatment cycle:

- Thyroid function test

Restaging scans will be repeated every 6 weeks regardless of treatment cycle. Tumor response will be assessed using RECIST version 1.1 and iRECIST. Baseline radiographic imaging will be performed with the relevant imaging to include CT scan of chest/abdomen/pelvis with or without contrast and/or MRI scan of abdomen/pelvis and all known or suspected sites of disease. MRI of brain (preferred) or Brain CT with contrast required for all subjects at baseline. Restaging scans will be performed every 6 weeks after the start of study treatment for the first 48 weeks and then every 12 weeks thereafter. CT of Chest and known sites of disease (e.g., abdomen with or without pelvis) is required for subjects with metastasis in those areas identified at baseline or if clinically indicated. The same method for tumor assessment should be employed at every assessment. Subjects with a history of brain metastasis to have surveillance MRI/CT approximately every 12 weeks or sooner if clinically indicated.

The Treatment Period ends when a subject receives his or her last dose of study treatment; the subject then enters the Follow-up Period.

5.3 Follow-up Period

Subjects should return 30 (±14) days after their last dose of study drug for an off-treatment visit to complete the following study procedures:

- Physical examination
- Vital signs and weight
- Concomitant medications
- ECOG performance status
- AE assessment
- CBC with differential
- Chemistries including LFTs
- Urinalysis
- Tumor assessment (CT and/or MRI scan). See Section 5.6 below.
- Future research blood for those subjects that sign consent

Additional follow-up may occur for subjects with AEs related to study drug that are ongoing at the time of this off-treatment visit unless the AE is deemed unresolvable or subject has started a new anti-cancer treatment regimen.

If a subject comes off treatment with no documented disease progression and no subsequent anti-cancer treatment received, they will have disease status (restaging scans) followed every 12 weeks (+/-2 weeks) until disease progression or start of new anti-cancer treatment regimen. Follow up for progression after the off-treatment visit can be conducted via phone interview or medical record review.

When subjects permanently discontinue study drug regimen, regardless of whether they are terminating the study early or have completed the study, subjects will undergo end of study regimen evaluation 30 days after the last dose of study drug or prior to starting any subsequent anticancer

therapy, they will return for a Safety Follow-up Visit, and information related to adverse events, the medications that subject might be taking, and ongoing cancer regimens will be collected.

After the Safety Follow up Visit, subjects will move into the Follow-up Period and will be contacted by telephone, email, or site visit approximately every 12 weeks (+/- 14 days), for up to 3 years, if subjects have come off the study for disease progression to check for any possible new serious adverse events, we will record the first subsequent anticancer therapy, and to check on subjects long-term health status.

5.4 Laboratory Assessments

Local laboratories will perform all clinical laboratory tests using standard procedures, and results will be provided to the Investigator. All abnormalities in clinical laboratory tests will be recorded on the case report form (CRF). If laboratory values constitute part of an event that meets criteria defining it as serious, the event (and associated laboratory values) must be reported as a serious adverse event (SAE).

Refer to Appendix C for details of laboratory tests for this study.

5.5 Adverse Event Assessment

AE definition is described in Section 10.1. AEs will be documented throughout the study. AE seriousness, grade, and relationship to study drug will be assessed by the Investigator using NCI-CTCAE version 5.0 at: https://ctep.cancer.gov/protocolDevelopment/electronic_applications/docs/CTCAE_v5_Quick_Reference_5x7.pdf.

SAE definitions and reporting requirements are described in Section 10.2.

Select non-serious and serious AEs also known as Events of Clinical Interest (ECI) must be recorded and reported as described in Section 10.3.

5.6 Tumor Assessments

Tumor response will be assessed using RECIST version 1.1 and iRECIST. Baseline radiographic imaging will be performed with the relevant imaging to include CT scan of chest/abdomen/pelvis with or without contrast and/or MRI scan of abdomen/pelvis and all known or suspected sites of disease. MRI of brain (preferred) or Brain CT with contrast required for all subjects at baseline. Restaging scans will be performed every 6 weeks after the start of study treatment for the first 48 weeks and then every 12 weeks thereafter. CT of Chest and known sites of disease (e.g., abdomen with or without pelvis) is required for subjects with metastasis in those areas identified at baseline or if clinically indicated. The same method for tumor assessment should be employed at every assessment. Subjects with a history of brain metastasis to have surveillance MRI approximately every 12 weeks or sooner if clinically indicated.

5.6.1 RECIST version 1.1

RECIST is a set of published rules that define when cancer subjects improve ("respond"), stay the same ("stabilize"), or worsen ("progression") during treatments. The original criteria were published in February 2000 by an international collaboration including the European Organization for Research and Treatment of Cancer (EORTC), National Cancer Institute (NCI) of the United States and the National Cancer Institute of Canada Clinical Trials Group. RECIST 1.1, published in January 2009, is an update to the original criteria and will be used for this study.

Refer to Appendix A for definition of target lesions, methods of measurement and all other related criteria for RECIST version 1.1. The following summarizes the definitions of the criteria used to determine objective tumor response for target lesions:

- **Complete Response (CR)**: Disappearance of all target lesions. Any pathological lymph nodes (whether target or non-target) must have reduction in short axis to <10 mm.
- **Partial Response (PR)**: At least a 30% decrease in the sum of diameters of target lesions, taking as reference the baseline sum diameters.
- **Progressive Disease (PD)**: At least a 20% increase in the sum of diameters of target lesions, taking as reference the smallest sum on study (this includes the baseline sum if that is the smallest on study). In addition to the relative increase of 20%, the sum must also demonstrate an absolute increase of at least 5 mm. (Note: the appearance of one or more new lesions is also considered progression).
- **Stable Disease (SD)**: Neither sufficient shrinkage to qualify for PR nor sufficient increase to qualify for PD, taking as reference the smallest sum diameters while on study.

5.6.2 iRECIST

Novel immunotherapeutics have been seen to trigger different response patterns in tumors than classic chemotherapy drugs, including the so-called 'pseudoprogressions', leading to concerns about assessing changes in tumors using existing tools as an objective evaluation of response to the treatment and disease progression. The iRECIST approach allows responses not typically observed in traditional systemic treatment to be identified and better documented. Table 6 summarizes the key principles of, and differences between, RECIST 1.1 and iRECIST:

Table 6: RECIST and iRECIST for Assessment of Tumor Response to TreatmentTable

	**RECIST 1.1**	**iRECIST**
Definitions of measurable and non-measurable disease; numbers and site of target disease	Measurable lesions are 10 mm or more in long diameter (15 mm for nodal lesions); maximum of 5 lesions (2 per organ); all other disease considered not target (must be 10 mm of longer in short axis for nodal disease)	No change; however, NEW lesions are evaluated as per RECIST 1.1 but are recorded separately on the CRF (but not included in the sum of lesions for target lesions identified at baseline)
CR, PR or SD	Cannot have met criteria for PD prior to CR, PR or SD	May have had iUPD (1 or more instances), but not iCPD, prior to iCR, iPR or iSD
Confirmation of CR, PR	Only required for non-randomised trials	As per RECIST 1.1
Confirmation of SD	Not required	As per RECIST 1.1

New lesions	Results in PD. Recorded but not measured	Results in iUPD but iCPD is only assigned based on this category if at next assessment<br>Additional NL appear or<br>Increase in size of NLs (≥5 mm for sum of NLT or any increase in NLNT)<br>NLs, where none have previously been recorded can also confirm iCPD
Independent blinded review and central collection of scans	Recommended in some circumstances	Collection of scans (but not independent review) recommended for all trials
Confirmation of PD	Not required (unless equivocal)	Required
Consideration of clinical status	Not included in assessment	Clinical stability (see definition) is considered in whether treatment is continued after iUPD

NT = non-target, T = target; NL = new lesions; NLT = new lesion target; NLNT = new lesion non target; CR = complete response; PD = progressive disease; SD = stable disease; iUPD = unconfirmed immune PD; iCPD = confirmed immune PD; SOM = sum of measures; iCR = immune complete response; iPR = immune partial response; iSD = immune stable disease

5.6.3 Imaging Central Review:

Baseline and restaging images should be acquired as outlined in Table 5, Study Calendar.
De-identified imaging will be requested for future submission.

5.7 Subject Withdrawal/Discontinuation

Subjects will receive study drug until treatment is discontinued for one of the reasons listed below. Subjects may discontinue study treatment or withdraw their consent to participate in the study at any time without prejudice. All reasons for discontinuation or withdrawal from trial will be recorded. The duration of treatment for GT103 will be until intolerable drug-related toxicity or disease progression. Reasons for subject discontinuation may include, but are not limited to, the following:

- Death
- Disease progression/treatment failure. Subjects felt to be clinically benefiting from treatment (e.g., mixed response, symptom improvement, demonstrable slowing of progression, e.g., progression rate of less than 20% over 6 months) may be continued on treatment with the agreement of the treating physician and study PI.
- Significant noncompliance by subject or treating physician
- Persistent (>6 weeks) NCI-CTCAE v5.0 grade 3 or 4 AE related to GT103 or any significant AE that compromises the subject's ability to safely continue to participate in the study. AE resulting in GT103 dosing delay for >6 weeks.
- If a subject requires dose reductions to more than two dose levels for non-hematologic toxicity, GT103 should be discontinued.

Any subject who misses more than 3 weeks (6 weeks from last dose) for treatment-related toxicity, GT103 should be discontinued.

- Investigator or Lead PI determination that it is no longer safe and/or no longer in the subject's best interest to continue participation
- Withdrawal of consent
- Lost to follow-up
- Necessity for treatment with other anticancer treatment prohibited by protocol
- Sexually active subjects who refuse to use medically accepted methods of contraception during the course of the study and for 6 months following the last dose of study drug
- Women who become pregnant or are breast feeding
- Request by regulatory agencies for termination of treatment of an individual subject or all subjects under the protocol
- Concurrent illness that prevents further administration of treatment
- The investigator for any reason, stops the study
- Termination of the study by GRID
- The compulsory detention for the treatment of either a psychiatric or physical (e.g., infectious disease) illness

6 STUDY DRUGS

6.1 Initial and Re-Supply Orders

Following submission and approval of the required regulatory documents, a supply of GT103 may be ordered from GRID Therapeutics. Please contact Scott Lyman at scott.lyman@gridthr.com to order GT103 study drug supply.

6.2 Treatment Compliance and Study Drug Accountability

The Investigator/and or delegate will maintain accurate records of receipt of all study drugs, including dates of receipt. In addition, accurate records will be kept regarding when and how much study drug is dispensed and used by each subject in the study. Reasons for deviation from the expected dispensing regimen must also be recorded. At completion of the study, to satisfy regulatory requirements regarding drug accountability, all unused study drug will be reconciled and destroyed in accordance with applicable state and federal regulations.

6.3 GT103

GT103 will be provided free of charge by Grid Therapeutics, LLC. GT103 will be supplied in a 10 mL glass vial with a rubber stopper and aluminum crimped seal. Each vial contains 200 mg GT103 (20 mg/mL). Each vial of study medication will be labeled clearly, disclosing the lot number, route of administration, Sponsor's name, and appropriate, required precautionary labeling.
GT103 injections must be stored in a secure area with limited access under controlled conditions at 2-8°C, protected from light. Partially used or empty vials will be destroyed per the Institutional Investigational Chemotherapy Pharmacy standard operating procedures.

6.3.1 Administration of GT103

GT103 will be administered intravenously over 60 minutes (+/- 5 minutes) on Day 1 of all cycles**. No premedication will be allowed for the first dose.** For the first two infusions at each dose level, every patient's vital signs (heart rate, respiratory rate, blood pressure and temperature) will be monitored at the following timepoints: within 60 minutes before, during (every 15 [±5] minutes), and 30 (±5) minutes, and 2 hour (± 15 minutes) and 4 hours (±15 minutes) and 6 hours (± 15 minutes) after the infusion. For subsequent infusions (if first two infusions were delivered without infusion-associated AEs), vital signs are to be collected within 60 minutes of infusion, during the infusion if clinically indicated and 1 hour (±10 minutes) after the end of infusion. Subjects will be informed about the possibility of delayed post-infusion symptoms and instructed to contact their study physician if they develop such symptoms.

The dosage of GT103 will be calculated on the weight obtained at C1d1. If a subject's body weight changes by >10%, dose should be recalculated.

Each dose will be prepared in 100 mL of D5W or Normal Saline for Injection in non-DEHP containing infusion bags and administered intravenously via an infusion pump through a non-DEHP containing intravenous administration set. The full recommended infusion volume should be used and if there are concerns for possible phlebitis, GT103 should be administered through a central line. For doses less than 1000 mg, GT103 will be administered as a 100 mL infusion volume that should be infused over 60 minutes. For doses of 1000 mg or larger, GT103 will be administered as a 250 mL infusion volume over 60 minutes.

All dosages prescribed and dispensed to the patient and all dose changes during the study will be recorded on the Dosage Administration Record CRF.

6.4 Concomitant Medications/Vaccinations

Concomitant medications will be documented in the subject record throughout the study. Subjects will be instructed not to take any additional medications (including over-the-counter products) during the course of the study without prior consultation with the investigator. At each visit, the investigator and/or research team member will ask the subject about any new medications he/she is taking or has taken after the start of the study drug. Medications or vaccinations specifically prohibited in the exclusion criteria are not allowed during the ongoing trial. If there is a clinical indication for one of these or other medications or vaccinations specifically prohibited during the trial, discontinuation from trial therapy if vaccination required. The Investigator should discuss any questions regarding this with the Lead PI.

6.4.1 Acceptable Concomitant Medications

All treatments that the Investigator considers necessary for a subject's welfare may be administered at the discretion of the Investigator in keeping with the local standards of medical care. All concomitant medication received from the date of signed informed consent through 30 days after the last dose of study drug should be recorded on the CRF including all prescription, over-the-counter (OTC), herbal supplements, and intravenous medications.

6.4.1.1 Contraception

Subjects should be informed that taking the study medication may involve unknown risks to the fetus (unborn baby) if pregnancy were to occur during the study. Non-pregnant, non-breast feeding women may be enrolled if they are willing to use 2 methods of birth control or are considered highly unlikely to conceive. Highly unlikely to conceive is defined as 1) surgically sterilized, or 2) postmenopausal women (a woman who is ≥45 years of age and has not had menses for greater than 1 year will be considered postmenopausal). The two birth control methods can be either two barrier methods or a barrier method plus a hormonal method to prevent pregnancy. Non-pregnant, non-breast feeding women must agree to use two birth control methods after informed consent is signed through 3 months after the last dose of study drug.

The following are considered adequate barrier methods of contraception: diaphragm, condom (by the partner), copper intrauterine device, sponge or spermicide. Appropriate hormonal contraceptives will include any registered and marketed contraceptive agent that contains an estrogen and/or a progestational agent (including oral, subcutaneous, intrauterine or intramuscular agents).

Males who are sexually active with WOCBP must agree to follow instructions for method(s) of contraception for the duration of treatment plus 90 days (duration of sperm turnover) for a total of 7 months post-treatment completion. In addition, male participants must be willing to refrain from sperm donation during this time.

Azoospermic males are exempt from contraceptive requirements. WOCBP who are continuously not heterosexually active are also exempt from contraceptive requirements, and still must undergo pregnancy testing as described in this protocol.

Investigators shall counsel WOCBP, and male participants who are sexually active with WOCBP, on the importance of pregnancy prevention and the implications of an unexpected pregnancy. Investigators shall advise on the use of highly effective methods of contraception which have a failure rate of < 1 % when used consistently and correctly.

6.4.2 Prohibited and/or Restricted Concomitant Medications

Subjects are prohibited from receiving the following therapies during the Screening and Treatment Period of this study:

- Other investigational therapy
- Anticancer agents other than the study medication should not be given to subjects, including chemotherapy, biological therapy, and immunotherapy. If such agents are required, then the subject must first be withdrawn from the study.
- Chronic treatment with systemic steroids or other immunosuppressive agents.
    - Intermittent steroids (not to exceed prednisone 10 mg every day or equivalent dosing) may be used on an as-needed basis (e.g., treatment for chemotherapy-related nausea, anorexia or fatigue).
    - Patients on physiologic replacement doses of steroids due to adrenal insufficiency for any reason may remain on those medications.
    - Topical, inhaled, or intra-articular corticosteroids are allowed.
- Subjects who, in the assessment by the Investigator, require the use of any of the aforementioned treatments for clinical management should be removed from the trial. Subjects may receive other medications that the Investigator deems to be medically necessary.

6.4.3 *Palliative Radiotherapy*

On study palliative radiotherapy in not allowed to target lesions used to assess disease response. Palliative radiotherapy to a single metastatic non-target lesions may be allowed with the approval of the PI in subjects who do not require immediate initiation of other systemic anti-cancer therapy.

There are no prohibited therapies during the Follow-up Period.

7 DOSE MODIFICATION AND TOXICITY MANAGEMENT

Subjects will be monitored continuously for AEs from the date of first study drug through 30 days after the last dose of treatment. Subjects will be instructed to notify their treating physician of any and all AEs. Toxicity will be graded according to NCI-CTCAE version 5.0. Study drug interrupted for > 3 weeks (6 weeks from last dose) in cycle ≥2 related to ANY hematological or non-hematological toxicity will be discontinued unless specified otherwise and/or approved by Principal Investigator. All interruptions or changes to study drug administration must be recorded.

All AEs should also be managed with supportive care at the earliest signs of toxicity considered related to study drug(s).

7.1 General Guidelines for Dose Modifications

Subjects experiencing one or more AEs due to the study drug may require dose modification(s) as described in Sections 7.3.1, 7.3.2, and 7.3.3.

Study drug interrupted for > 3 weeks (6 weeks from last dose) in cycle ≥ 2 related to ANY hematological or non-hematological toxicity will be discontinued unless specified otherwise and/or approved by Principal Investigator. Subjects may need to be followed at least weekly when any study drug is held for toxicity until the toxicity returns to Grade ≤ 1 or is determined to be chronic or irreversible.

Dosing interruptions are permitted in Cycle 2 and up in the case of medical/surgical events or logistical reasons not related to study therapy (e.g., elective surgery, unrelated medical events, patient vacation, and/or holidays). Subjects should be placed back on study therapy within 3 weeks of the scheduled interruption for cycles 2 and up, unless otherwise discussed with the Lead PI. The reason for interruption should be documented in the patient's study record.

Given the potential immunomodulatory effects of GT103, standard institutional treatment of immune mediated toxicity should be initiated. Clinical guidance is provided below and additionally available per reference [24].

All dose modifications and reasons for modification must be recorded in the CRF.

7.2 Treatment Parameters for Day 1 of Each Cycle (Stage 1a/b)

Treatment on Day 1 of any cycle should be delayed until:

- Hemoglobin ≥ 8 g/dL

- Absolute neutrophil count (ANC) is ≥ 1,000/mm^3 **and** platelet count is ≥ 75,000/mm^3
- Recovery to baseline or ≤ grade 1 from any **clinically significant** treatment-related non-hematological toxicity as outlined below. For other toxicities not listed in the following tables, subject may continue drug with grade 2 toxicity if not considered clinically significant by treating physician.
- Laboratory only abnormalities without symptoms do not require treatment delays, except as specified below. However, caution is needed to ensure re-treatment is considered safe and in the interest of the subject.
- Corrected QTc interval must be <480 msec. Per study calendar, if QTc interval is >480 msec, then two additional 12 lead ECGs should be obtained over a brief period of time (e.g., within 15-20 minutes) to confirm the abnormality. The average QTc interval will be determined from the triplicate ECGs by manual evaluation would require dose hold for grade 2 QTc>480 in CXD1s.per .

7.3 Dose Modifications for GT103

7.3.1 General Guidelines for GT103 Dose Modifications

The following are guidelines for dose modifications of GT103:

- If there are treatment delays occur the study calendar will move, and held doses of treatment will be made up.
- If no "lower" dose modification exists, discontinue drug.
- If more than one dose reduction applies, then use the most stringent (i.e., the greatest dose reduction).
- Grade 1 hematology toxicities will not result in dose modifications.
- If a subject experiences a grade 4 event more than once, retreatment (even with dose modifications) is permitted only with permission of the Principal Investigator.
- If any treatment agent is held longer than 14 days due to delayed recovery from toxicity or intolerance, and a dose modification is not already specified in the following tables, the treating physician may elect to resume that agent at one dose level lower.
- Additional dose holding and/or reduction is also permitted when these are judged in the patient's interest by the treating physician (e.g., due to multiple toxicities, persistent toxicities, intercurrent illness, or short-term compliance or monitoring issues).
- The PI should be notified of any dose modifications or dose holding for toxicity.
- For immune related AE of grade 4 (other than endocrinopathy), patients will not receive retreatment.

Clinically non-significant laboratory only abnormalities without symptoms, which are not treatment-related, do not require treatment delays. This applies to intercycle and intracycle assessments.

If a subject requires dose reductions to more than two dose levels for non-hematologic toxicity, GT103 should be discontinued.

Any subject who misses more than 3 weeks (6 weeks from last dose) for treatment-related toxicity, GT103 should be discontinued.

Dose holding and/or reduction is also permitted when these are judged to be in the patient's interest by the treating physician.

7.3.2 Hematologic Toxicity Criteria (Related to GT103)

- Dose modifications for hematologic toxicity should be independently assessed at each study drug administration visit as described in Table 7.
- Prophylactic G-CSF may be implemented in cycles 2 and above following ASCO guidelines.
- G-CSF may be implemented in the management of neutropenia to avoid dose reductions or holding a dose if neutropenia recovers within 48 hours.

Table 7: Hematologic CTCAE 5.0 Criteria for Dose Delay and Dose Modification

Grade	Delay	Dose
1	No	100% of current dose
2	No	75% of current dose
3*	Until grade ≤ 1[a]	75% of current dose
4*	Until grade ≤ 1[a]	50% of current dose
[a]If no recovery to grade ≤ 2 after a 3 week delay (6 weeks from last dose), study drug should be discontinued		

*For febrile neutropenia, defined as ANC <500/µl and temperature ≥38.2°C (100.8°F), GT103 should be reduced 25% in all subsequent cycles.

7.3.3 Non-Hematologic Toxicity Criteria (Related to GT103)

Table 8: Non-Hematologic Toxicity Criteria

Grade	Details	Hold Dose	% of Dose after Recovery to Grade 0-1
2	except for nausea, vomiting, diarrhea, alopecia and fatigue	Hold dose until resolution to Grade 0 or 1	100% of current dose
	Intolerable skin toxicity	Hold dose until resolution to Grade 0 or 1	75% of current dose
3	Except for nausea and vomiting	Hold dose until resolution to Grade 0 or 1	75% of current dose
4	Life-threatening conditions (study drug-related)	Treatment should be discontinued (unless endocrinopathy ~~and~~ or amylase/lipase Sec 7.6	NA

7.4 General Supportive Care Guidance

Treatments should be guided by institutional standard of care or local guidelines.

7.4.1 Hematopoietic Growth Factors

Growth factors (e.g., G-CSF, GM-CSF, erythropoietin, platelet growth factors, etc.) may be prescribed in cycles 2 and all subsequent cycles by the investigator for rescue from severe hematologic events if clinically appropriate. Therapeutic growth factor usage is permitted per ASCO and treating physician guidelines.

7.4.2 Pulmonary Adverse Events

Rule out non-inflammatory causes. If a non-inflammatory cause, treat accordingly and follow modifications in Section 7.3.3. Evaluate with imaging and pulmonary consultation.

- For **Grade 1 events**, consider delay of therapy. Monitor for symptoms every 2-3 days, consider pulmonary and ID consults. Re-image at least every 3 weeks.
- For recurrent **Grade 2 events**, delay therapy per protocol. Consider bronchoscopy, lung biopsy, and pulmonary and ID consults. Monitor symptoms daily, consider hospitalization. 1.0 mg/kg/day methylprednisolone IV or oral equivalent. Re-image every 1-3 days. If symptoms return to near baseline, taper steroids over at least 1 month and then resume therapy per protocol and consider prophylactic antibiotics.
- For **Grade 3-4 events**, discontinue therapy per protocol. Hospitalize, get pulmonary and ID consults. Give 2-4 mg/kg/day methylprednisolone IV or IV equivalent. Add prophylactic antibiotics for opportunistic infections. Consider bronchoscopy, lung biopsy. If symptoms return to baseline, taper steroids over at least 6 weeks. If not improving after 48 hours or worsening, add additional immunosuppression.

Patients on IV steroids may be switched to an equivalent dose of oral corticosteroids (e.g., prednisone) at start of tapering or earlier, once sustained clinical improvement is observed. Lower bioavailability of oral corticosteroids should be taken into account when switching to the equivalent dose of oral corticosteroids.

7.4.3 GI Adverse Events

Rule out non-inflammatory causes. If non-inflammatory cause is identified, treat accordingly and follow modifications in Section 7.3.3. Opiates/narcotics may mask symptoms of perforation. Infliximab should not be used in cases of perforation or sepsis.

- For **Grade 1** diarrhea/colitis, continue therapy per protocol and administer symptomatic treatment. Monitor closely for worsening symptoms and educate patient to report worsening immediately.
- For **Grade 2** diarrhea/colitis, delay therapy per protocol and administer symptomatic treatment. If it improves to grade 1, resume therapy per protocol. If it persists > 5-7 days or recurs, give 0.5-1.0 mg/kg/day methylprednisolone or oral equivalent. When symptoms improve to grade 1,

taper steroids over at least 1 month, consider prophylactic antibiotics for opportunistic infections, and resume therapy per protocol.

- For **Grade 3 or 4** diarrhea/colitis, discontinue therapy per protocol. Administer 1.0 to 2.0 mg/kg/day methylprednisolone IV or IV equivalent. Add prophylactic antibiotics for opportunistic infections, consider lower endoscopy. If it improves, continue steroids until grade 1, then taper over at least 1 month. If it persists > 3-5 days or recurs after improvement, add infliximab 5 mg/kg (if no contraindication).

Patients on IV steroids may be switched to an equivalent dose of oral corticosteroids (e.g., prednisone) at start of tapering or earlier, once sustained clinical improvement is observed. Lower bioavailability of oral corticosteroids should be taken into account when switching to the equivalent dose of oral corticosteroids.

7.4.4 Renal Adverse Events

Rule out non-inflammatory causes. If non-inflammatory cause, treat accordingly and follow modifications in Section 7.3.3.

- For **Grade 1** events, continue therapy per protocol, monitor creatinine weekly. If it returns to baseline, resume routine creatinine monitoring per protocol.
- For **Grade 2-3** events, delay therapy per protocol, and monitor creatinine every 2-3 days. Administer 0.5 to 1.0 mg/kg/day methylprednisolone IV or oral equivalent. Consider a renal biopsy with nephrology consult. If it returns to grade 1, taper steroids over at least 1 month, consider prophylactic antibiotics for opportunistic infections, and resume therapy and routine creatinine monitoring per protocol.
- For **Grade 4** events, discontinue therapy per protocol, and monitor creatinine daily. Administer 1.0 to 2.0 mg/kg/day methylprednisolone IV or IV equivalent. Consult a nephrologist and renal biopsy. If it returns to grade 1, taper steroids over at least 1 month and add prophylactic antibiotics for opportunistic infections.

Patients on IV steroids may be switched to an equivalent dose of oral corticosteroids (e.g., prednisone) at start of tapering earlier, once sustained clinical improvement is observed. Lower bioavailability of oral corticosteroids should be taken into account when switching to the equivalent dose of oral corticosteroids.

7.4.5 Hepatic Adverse Events

Rule out non-inflammatory causes. If non-inflammatory cause, treat accordingly and follow modifications in Section 7.3.3. Consider imaging for obstruction.

- For **Grade 1** events, continue therapy per protocol, and continue LFT monitoring per protocol.
- For **Grade 2** events, delay therapy per protocol and increase frequency of monitoring to every 3 days. If symptoms return to baseline, resume routine monitoring and resume therapy per protocol. If elevations persist > 5-7 days or worsen, administer 0.5-1.0 mg/kg/day methylprednisolone or oral equivalent and when LFT returns to grade 1 or baseline, taper steroids over at least 1 month, consider prophylactic antibiotics for opportunistic infections, and resume therapy per protocol.

- For **Grade 3-4** events, discontinue therapy (may be delayed rather than discontinued if AST/ALT ≤ 8 x ULN or T.bili ≤ 5 x ULN), and increase frequency of monitoring to every 1-2 days. Administer 1.0 to 2.0 mg/kg/day methylprednisolone IV or IV equivalent (recommended starting dose for grade 4 hepatitis is 2 mg/kg/day IV). Add prophylactic antibiotics for opportunistic infections and consult a gastroenterologist. If symptoms return to grade 2, resume routine monitoring and resume therapy per protocol. If they do not improve in >3-5 days, worsen or rebound, add mycophenolate mofetil 1 g BID. If no response within an additional 3-5 days, consider other immunosuppressants per local guidelines.

Patients on IV steroids may be switched to an equivalent dose of oral corticosteroids (e.g., prednisone) at start of tapering or earlier, once sustained clinical improvement is observed. Lower bioavailability of oral corticosteroids should be taken into account when switching to the equivalent dose of oral corticosteroids.

7.4.6 Endocrinopathy Adverse Events

Rule out non-inflammatory causes. If non-inflammatory cause, treat accordingly and follow modifications in Section 7.3.3. Consider visual field testing, endocrinology consultation, and imaging.

- For **Asymptomatic TSH Abnormality,** continue therapy per protocol. If TSH < 0.5 x LLN, or TSH > 2 x ULN, or consistently out of range in 2 subsequent measurements, include free T4 at subsequent cycles as clinically indicated; consider endocrinology consult.
- For **Symptomatic Endocrinopathy,** evaluate endocrine function and consider a pituitary scan. For subjects who are symptomatic with abnormal lab/pituitary scan, administer 1-2 mg/kg/day methylprednisolone IV or PO equivalent, and initiate appropriate hormone therapy. If there are no abnormal labs or pituitary MRI scan, but symptoms persist, repeat labs in 1-3 weeks and repeat MRI in 1 month. If the symptoms improve with or without hormone replacement, taper steroids over at least 1 month and consider prophylactic antibiotics for opportunistic infections. Patients with adrenal insufficiency may need to continue steroids with mineralocorticoid component.
- **Suspicion of Adrenal Crisis** – delay or discontinue therapy per protocol and rule out sepsis. Administer stress dose of IV steroids with mineralocorticoid activity and IV fluids. Consult endocrinologist. If adrenal crisis is ruled out, then treat as above for symptomatic endocrinopathy.

Patients on IV steroids may be switched to an equivalent dose of oral corticosteroids (e.g., prednisone) at start of tapering or earlier, once sustained clinical improvement is observed. Lower bioavailability of oral corticosteroids should be taken into account when switching to the equivalent dose of oral corticosteroids.

7.4.7 Skin Adverse Events

Rule out non-inflammatory causes. If non-inflammatory cause, treat accordingly and continue follow modifications in Section 7.3.3.

- For **Grade 1-2** events, continue GT103 therapy per protocol and administer symptomatic therapy. If symptoms persist > 1-2 weeks or recur, consider a skin biopsy and delay therapy per

protocol. Consider 0.5-1.0 mg/kg/day methylprednisolone IV or oral equivalent. Once improving, taper the steroids over at least 1 month, consider prophylactic antibiotics for opportunistic infections, and resume therapy per protocol.

- For **Grade 3-4** events, delay or discontinue therapy per protocol. Get a dermatology consult and consider a skin biopsy. Administer 1.0-2.0 mg/kg/day IV methylprednisolone IV or IV equivalent. If signs improve to grade 1, taper steroids over at least 1 month and add prophylactic antibiotics for opportunistic infections, and resume therapy per protocol.

Patients on IV steroids may be switched to an equivalent dose of oral corticosteroids (e.g., prednisone) at start of tapering or earlier, once sustained clinical improvement is observed. Lower bioavailability of oral corticosteroids should be taken into account when switching to the equivalent dose of oral corticosteroids. If SJS/TEN is suspected, withhold therapy and refer patient for specialized care for assessment and treatment. If SJS or TEN is diagnosed, permanently discontinue therapy.

7.4.8 Neurologic Adverse Events

Rule out non-inflammatory causes. If non-inflammatory cause, treat accordingly and follow modifications in Section 7.3.3.

- For **Grade 1** events, continue therapy per protocol. Continue to monitor the patient.
- For **Grade 2** events, delay therapy per protocol. Treat symptoms per local guidelines. Consider 0.5 to 1.0 mg/kg/day methylprednisolone IV or PO equivalent. If signs improve to baseline, resume therapy per protocol.
- For **Grade 3-4** events, discontinue therapy per protocol, and obtain neurology consult. Treat symptoms per local guidelines. Administer 1.0-2.0 mg/kg/day IV methylprednisolone IV or IV equivalent and add prophylactic antibiotics for opportunistic infections. If signs improve to grade 2, taper steroids over at least 1 month. If they worsen, or atypical presentation, consider IVIG or other immunosuppressive therapies per local guidelines.

Patients on IV steroids may be switched to an equivalent dose of oral corticosteroids (e.g., prednisone) at start of tapering or earlier, once sustained clinical improvement is observed. Lower bioavailability of oral corticosteroids should be taken into account when switching to the equivalent dose of oral corticosteroids.

7.4.9 Infusion Reactions

An infusion reaction for GT103 may manifest with fever, chills, rigors, headache, rash, pruritus, arthralgias, hypo- or hypertension, bronchospasm, or other symptoms. Infusion reactions should be graded according to NCI CTCAE (Version 5.0) guidelines.
Treatment recommendations are provided below and may be modified based on local treatment standards and guidelines as appropriate:

For <u>Grade 1</u> symptoms (mild reaction; infusion interruption not indicated; intervention not indicated):

- Remain at bedside and monitor subject until recovery from symptoms. The following prophylactic premedications are recommended for future infusions: diphenhydramine 50

mg (or equivalent) and/or acetaminophen/paracetamol 325 to 1000 mg at least 30 minutes before additional study drug administrations.

For Grade 2 symptoms (moderate reaction that requires therapy or infusion interruption but responds promptly to symptomatic treatment [e.g., antihistamines, non-steroidal anti-inflammatory drugs, narcotics, corticosteroids, bronchodilators, IV fluids]; prophylactic medications indicated for ≤ 24 hours):

- Stop the study drug infusion, begin an IV infusion of normal saline, and treat the subject with diphenhydramine 50 mg IV (or equivalent) and/or acetaminophen/paracetamol 325 to 1000 mg; remain at bedside and monitor subject until resolution of symptoms. Corticosteroid and/or bronchodilator therapy may also be administered as appropriate. If the infusion is interrupted, then restart the infusion at 50% of the original infusion rate when symptoms resolve; if no further complications ensue after 30 minutes, the rate may be increased to 100% of the original infusion rate. Monitor subject closely. If symptoms recur, then no further study drug will be administered at that visit. Administer diphenhydramine 50 mg IV, and remain at bedside and monitor the subject until resolution of symptoms. The amount of study drug infused must be recorded on the electronic case report form (eCRF).
- For future infusions, the following prophylactic premedications are recommended: diphenhydramine 50 mg (or equivalent) and/or acetaminophen/paracetamol 325 to 1000mg should be administered at least 30 minutes before study drug infusions. If necessary, corticosteroids (up to 25 mg of Solucortef or equivalent) may be used.

For Grade 3 or 4 symptoms: (severe reaction, Grade 3: prolonged [i.e., not rapidly responsive to symptomatic medication and/or brief interruption of infusion]; recurrence of symptoms following initial improvement; hospitalization indicated for other clinical sequelae [e.g., renal impairment, pulmonary infiltrates]. Grade 4: Life threatening; pressor or ventilator support indicated):

- Immediately discontinue infusion of study drug. Begin an IV infusion of normal saline and treat the subject as follows: Recommend bronchodilators, epinephrine 0.2 to 1 mg of a 1:1000 solution for subcutaneous administration or 0.1 to 0.25 mg of a 1:10,000 solution injected slowly for IV administration, and/or diphenhydramine 50 mg IV with methylprednisolone 100 mg IV (or equivalent), as needed. Subject should be monitored until the investigator is comfortable that the symptoms will not recur. Study drugs will be permanently discontinued. Investigators should follow their institutional guidelines for the treatment of anaphylaxis. Remain at bedside and monitor subject until recovery of the symptoms.

In case of late-occurring hypersensitivity symptoms (e.g., appearance of a localized or generalized pruritus within 1 week after treatment), symptomatic treatment may be given (e.g., oral antihistamine or corticosteroids).

7.5 Treatment Beyond Progression

Accumulating evidence indicates a minority of subjects treated with immunotherapy may derive clinical benefit despite initial evidence of PD. Subjects with new lesions that are ambiguous or uncertain, may continue treatment until progression is confirmed on future scans. Subjects will be

permitted to continue on GT103 for treatment beyond initial RECIST 1.1 defined PD as long as they meet the following criteria:

- Investigator-assessed clinical benefit and no rapid disease progression
- Subject is tolerating study treatment
- Stable performance status
- Treatment beyond progression will not delay an imminent intervention to prevent serious complications of disease progression (e.g., CNS metastases)
- **Subject provides written informed consent prior to receiving additional GT103 treatment using an ICF describing any reasonably foreseeable risks or discomforts, or other alternative treatment options.**

The decision to continue treatment beyond initial investigator-assessed progression should be discussed and approved by the PI and documented in the study records.

For subjects who continue study therapy beyond progression, further progression is defined as an additional 10% increase in tumor burden from the time of initial PD. This includes an increase in the sum of diameters of all target lesions and/ or the diameters of new measurable lesions compared to the time of initial PD. GT103 treatment should be discontinued permanently upon documentation of further progression. Continue RECIST 1.1.

New lesions are considered measurable at the time of initial progression if the longest diameter is at least 10 mm (except for pathological lymph nodes which must have a short axis of at least 15 mm). Any new lesion considered non-measurable at the time of initial progression may become measurable and therefore included in the tumor burden if the longest diameter increases to at least 10 mm (except for pathological lymph nodes which must have a short axis of at least 15 mm). In situations where the relative increase in total tumor burden by 10% is solely due to inclusion of new lesions which become measurable, these new lesions must demonstrate an absolute increase of at least 5 mm.

7.6 Criteria to Resume GT103

Participants may resume treatment when the drug-related AE(s) resolve(s) to Grade ≤ 1 or baseline, with the following exceptions:

- Participants may resume treatment in the presence of Grade 2 fatigue.
- Participants with Grade 3-4 AST/ALT and/or total bilirubin values meeting discontinuation parameters should have treatment permanently discontinued.
- Drug-related pulmonary toxicity, diarrhea, or colitis must have resolved to baseline or </= grade 1 before treatment is resumed. Participants with persistent Grade 1 pneumonitis after completion of a steroid taper over at least 1 month may be eligible for retreatment if discussed with and approved by the PI.
- Participants who received systemic corticosteroids for management of any drug-related toxicity must be off corticosteroids or have tapered down to an equivalent dose of prednisone ≤ 10 mg/day.
- Drug-related endocrinopathies adequately controlled with only physiologic hormone replacement may resume treatment. Steroid doses equivalent to prednisone ≥ 10 mg/day are permissible if needed for physiologic hormone replacement.
- Participants who delay study treatment due to any Grade ≥ 3 amylase or lipase abnormality that is not associated with symptoms or clinical manifestations of pancreatitis, and that is

assessed by the investigator, may resume GT103 when the amylase or lipase abnormality has resolved to Grade 1 or baseline at 75% of current dose.

8 CORRELATIVES

Blood will be collected for PK analysis and for the analysis of complement factors—efficacy biomarkers that are evidence of complement lysis—before treatment and at time points given below. Blood will separately be collected for future measurement of potential efficacy biomarkers such as tumor cell-free DNA, a measure of tumor cell lysis, cytokines induced by the treatment, or antitumor antibodies, a measure of an adaptive immune response. Additional markers related to efficacy, toxicity, and/or cancer biology (e.g., angiogenesis, inflammation, immunity, and tumor growth) may be analyzed in the future using these blood samples.

Exploratory tissue-based biomarker studies will be performed to identify biomarkers that correlate with response to GT103 therapy. Such biomarkers may be tested in the future for their predictive value, allowing selection of patients who may best respond to GT103 therapy. Initial correlative analyses will focus on complement regulatory proteins CD55, CD59, and CFH that are measured on tumor cell membranes in archived subject tumor tissue. Additional predictive biomarkers (e.g., immune cell content of tumors, or mutations in the tumor or subject germline) may be explored.

Available resources and evolving scientific knowledge will drive what additional biomarker analyses will be considered. External vendors and collaborators may conduct or participate in current or future analyses only for Duke IRB approved projects, provided samples are deidentified. All collaborations will be subject to Institutional policies on sample and data sharing.

8.1 Blood Collection

For blood collection procedures, refer to Appendix D, *Study Manual*.

8.2 Pharmacokinetics (PK)

Blood for PK will be collected pre-dose on Day 1, and at 2 hours +/- 10 minutes, 4 hours +/- 10 minutes, 8 hours +/- 15 minutes, and 24 hours +/- 30 minutes after the start of infusion of the first dose of GT103. Subjects will be required to remain in the study clinic on Day 1 up through the PK blood sample taken 8 hours after the first dose of GT103. PK samples will also be collected on Cycle 1 Days 8 and 15 and pre-dose Cycles 2-5. Samples will be stored and analyzed in the Phase I Biomarker Laboratory under the direction of Dr. Andrew Nixon.

<u>Assay for the measurement of blood concentration of GT103.</u> We will assay GT103 in blood using an ELISA developed in the Patz laboratory. Biotinylated peptide containing the epitope for GT103 is immobilized on NeutrAvidin in wells of an ELISA plate. Blood samples are diluted in PBS. Antibody in each diluted sample is captured on the peptide and is detected with a goat anti-human IgG gamma-chain-horseradish peroxidase (HRP) conjugate. The ELISA is developed with the HRP substrate ABTS, the plate is read at 405 nm, and antibody concentration is determined. See Appendix D, *Study Manual* for detailed protocol.

<u>Validation of the GT103 assay.</u> To be used for PK determination, proper analytical validation of the GT103 assay is paramount. The assay performance characteristics will be investigated using a variety

of measures, including various metrics of assay specificity and sensitivity. Key metrics will include: standard curve and dynamic range optimization, sensitivity, coefficients of variation, matrix effects, sample stability and assay specificity. For the sensitivity of the assay, we will establish the Limits of Detection (LOD), Lower Limits of Quantitation (LLOQ) and Upper Limits of Quantitation (ULOQ). LOD is the mean of the zero calibration standards + 2 standard deviations, LLOQ is the lowest calibration standard with back-calculated concentrations having CVs <20% and relative error <25%, and ULOQ is the highest calibration standard with back-calculated concentrations having CVs <20% and relative error <20%.

8.3 Complement Factors (C3a and sC5b-9)

Subjects will have blood collected for analysis of complement factors pre-dose on Cycle 1 Day 1 and at 2 hours +/- 10 minutes, 4 hours +/- 10 minutes, 8 hours +/- 15 minutes, and 24 hours +/- 30 minutes after the **start** of infusion first dose of GT103, as well as on Cycle 1 Days 8 and 15 (level 4a will not have a day 15 collection) and pre-dose Cycles 2-5. Samples will be stored and analyzed in the Phase I Biomarker Laboratory under the direction of Dr. Andrew Nixon.

> Assay for C5b-9. We will assay these products of the complement cascade in patient plasma using the commercial ELISA kits described in Appendix D, *Study Manual*.
>
> Validation of the commercial C5b-9 ELISA assays. We will characterize the standard curves and optimize the dynamic ranges for each assay, as well as determining the sensitivity, coefficients of variation, matrix effects, sample stability and assay specificity of the C5b-9 assays.

8.4 Blood Biomarkers for Future Research

Subjects that have signed informed consent will have blood collected for future analysis of biomarkers to include but be not limited to cfDNA, cytokines, and antitumor antibodies. Time points for collection are pre-dose on Cycle 1 Day 1, on Cycle 1 Day 8, pre-dose on Day 1 of all subsequent cycles; and at the 30 day follow-up. An additional blood sample will be collected upon progression.

8.5 Anti-Drug Antibodies

All subjects will be evaluated for the development of anti-GT103 antibodies over time. Plasma to be assayed will be isolated from blood collected in purple top tubes pre-dose on Day 1 of Cycles 1-5. Plasma will be stored at -80 ºC in bar-coded vials and batch run in a fully validated assay (see Appendix D).

8.6 Tumor Tissue Biomarkers

Archived FFPE tumor tissue will be collected for all subjects (if available). Tumor cell expression of CD55, CD59, and CFH will be detected by immunohistochemistry (IHC) using primary mAbs CD55 [EPR6689], CD59 [EPR6425(2)] OX-24 [ab118820] (Abcam, Cambridge, MA), respectively, with the pathologist scoring reactivity on an integer scale. See Appendix D. Samples to be cut at the end of the study enrollment.

8.7 Future Use of Patient Samples

Any remaining biological materials at the end of the study will be deidentified, and deidentified samples may be retained for possible use in biomarker research with the consent of the patient.

9 STATISTICAL METHODS AND DATA ANALYSIS

All statistical analysis will be performed under the direction of the statistician designated in key personnel. Any data analysis carried out independently by the investigator must be approved by the statistician before publication or presentation.

9.1 Analysis Sets

Analysis Set for Determination of MTD: Subjects who experience a DLT at any time during the DLT evaluation window (defined as the first 21-day cycle for dose levels 1-5 or two 2 week cycles for dose level 4a) or who completed cycle 1 without incident DLT will be considered evaluable for the determination of MTD. Subjects withdrawn from the study without receipt of any GT103 treatment and subjects who are withdrawn from the study without a DLT but prior to completion of the DLT evaluation window will be replaced for the determination of MTD.

Safety Analysis Set: All subjects who receive at least one dose of GT103 will be evaluated for safety in this trial. However, not all subjects who receive GT103 will be evaluable for the determination of MTD.

Efficacy Analysis Set: All subjects who receive any GT103 will be evaluated for efficacy in this trial.

9.2 Patient Demographics and Other Baseline Characteristics

The following baseline patient characteristics will be summarized: Baseline medical history, ECOG performance status, symptoms, gender, age, race, histology, stage, and type of prior therapy. Frequency distributions will be generated for categorical descriptors; whereas, means, standard deviations, and quantiles will be generated to describe continuous variables.

9.3 Treatments

Frequency distributions will be generated to describe the number of subjects treated at each dose level, as well as the number of cycles of treatment that the patient may receive.

9.4 Primary Objectives

This study has two primary objectives: to define the MTD (if any) and the RP2D of GT103 for the treatment of adult subjects with refractory, advanced stage NSCLC, and to characterize the PK profile of GT103 when delivered to subjects with refractory, advanced stage NSCLC.

9.4.1 Primary Objective #1: Maximum Tolerated Dose (MTD) of GT103 Monotherapy

9.4.1.1 Variables

Two outcome measures will be determined as part of this primary objective, including:

- The number and proportion of subjects at each dose level who experience a DLT, as defined in Section 3.2.
- The RP2D as defined in Section 3.2.

9.4.1.2 Dose Escalation

Details concerning dose escalation are found in Section 3.2, along with information about what constitutes a RP2D.

9.4.1.3 Additional Safety Analyses

In addition to the analyses described above for the determination of the RP2D, AEs will be summarized in several other forms to satisfy scientific and monitoring needs, including various regulatory reporting needs (e.g., FDA, Safety Oversight Committee, and ClinicalTrials.gov). For the primary outcome of safety, the proportion of subjects who experience a DLT at each dose level will be reported as well as the RP2D. These particular reports focus on AEs occurring during the DLT observation period.

Separate from the reporting of the primary outcome, additional summaries of AEs within ClinicalTrials.gov will focus on all AEs occurring during or after the DLT observation period. The proportion of subjects who experience serious AEs regardless of grade or attribution will be reported in ClinicalTrials.gov for each type of AE. In addition, the proportion of subjects who experience each type of AE regardless of grade or attribution will also be reported.

For the FDA, two other summaries of AEs may be generated: (1) A frequency distribution of AEs tabulated by the maximum grade of each type of AE regardless of attribution, and (2) A frequency distribution of AEs tabulated by the maximum grade of each type of AE that is possibly, probably, or definitely related to the protocol treatment prescribed in this study. These tabulations will be stratified by GT103 dose.

9.4.2 Primary Objective #2: Pharmacokinetic (PK) Study

The second primary objective is to describe the PK profile of GT-103 when delivered to subjects with refractory advanced NSCLC. A population PK analysis will be performed to characterize the PK of GT103 in patients with refractory NSCLC tumors using the software Nonlinear Mixed Effects Modeling (NONMEM, version 7.2). Different structural PK models (e.g., 1 and 2 compartment) with linear or non-linear (e.g., Michaelis-Menten) kinetics will be fitted to the plasma concentration-time data of GT103. Inter-individual variability on model parameters and different residual error models will be tested. Covariate evaluation will be performed to examine the relationship between model parameters and clinical factors (e.g., age, weight, serum albumin). Appropriate covariates will be incorporated into the model using a standard forward inclusion-backward elimination approach.

Model development will be guided by goodness of fit plots, plausibility of parameter estimates, and reduction in inter-individual variability for structural and residual error parameters, as well as objective function and shrinkage values.

Model validation will be performed using visual predictive check and bootstrapping. Using the final PK model and parameter estimates, plasma concentration-time profiles of GT103 will be simulated for each participant at varied dosing regimens. The optimal GT103 dosing regimen will be selected according to pre-specified drug exposure target criteria.

9.5 Secondary Objectives

Briefly, our secondary objectives are to describe the response rate associated with GT103 treatment as monotherapy, and to describe the progression-free survival (PFS) and overall survival (OS) of subjects treated with GT103.

9.5.1 Secondary Objective #1: Response Rate

The initial secondary objective is to describe the response rate associated with GT103 as monotherapy treatment overall, with 95% confidence intervals. Response rate is defined as the proportion of treated subjects with a complete or partial response. The response rate associated with the dose chosen to be the RP2D will also be estimated. Logistic regression may be used to explore the association between dose and response.

9.5.2 Secondary Objective #2: Progression-Free Survival and Overall Survival

Another secondary objective is to describe PFS and OS. PFS is defined as the time between initiation of treatment and initial failure (disease progression or death). If the patient remains alive without disease progression at the time of analysis, PFS will be censored at the time of last follow-up. If the patient starts alternative anti-cancer therapy before progression, PFS will be censored at the time that the alternative treatment is initiated. OS is defined as the time between initiation of treatment and death. OS will be censored at the time of last follow-up if the patient remains alive at the time of analysis. (Note: Within the correlative analyses, PFS and OS will be calculated from the day 21 landmark.)

A Kaplan-Meier curve will graphically describe PFS and OS overall and among subjects treated at the RP2D. Estimates of median survival, as well as 6 and 12-month survival will be estimated with confidence intervals. The impact of dose on PFS or OS may be explored in the context of a Cox model.

9.6 Exploratory Objectives

Exploratory objectives include an examination of serum-based and tumor-based biomarkers, as well as imaging features. Specifically, we will examine assay biomarkers to inform us that GT103 is having an anti-tumor effect. We will also examine tumor biomarkers to assess whether tumor-based characteristics correlate with response to therapy and are potentially predictive. We will also examine imaging features that may be predictive of a response, survival, or toxicity.

Many of the analyses proposed below assume methods for normally distributed data are appropriate. If underlying assumptions for planned analyses are violated, plans will be modified. Alternative analytic methods may also be considered to assess the robustness of inferences.

Additional exploratory statistical analyses may be conducted besides those described below in this protocol. These additional analyses may be motivated by evolving research outside of this protocol, or they may be suggested by statistical analyses pre-specified in this protocol.

9.6.1 Exploratory Objective #1: Serum-Based Biomarkers

We intend to explore biomarkers that are associated with mechanisms and outcomes. Specifically, we will explore levels of C3a and sC5b-9 in serum.

Biomarkers of CDC: We hypothesize that GT103 will facilitate cell killing which in turn improves outcome. Biomarkers of CDC include C3a and sC5b-9. Subjects will have blood collected for complement factors at baseline and at 2, 4, 8, and 24 hours after the first dose of GT103, as well as Cycle 1 Day 8 and Day 15, and predose Day 1 of all subsequent cycles. The mean levels of analytes will be calculated at baseline and each follow-up assessment. A repeated measures analysis that accounts for within-subject correlation will assess the impact of dose, time, and their interaction on analyte levels. The interaction included in this model will assess whether changes over time are consistent across doses.

The impact of these changes on response, OS, and PFS will also be explored. Change may be incorporated into the model as the change between baseline and Day 21, or it might be a variable that describes the pattern of change observed over the 21-day period. This predictor will be incorporated into a logistic model to assess its effect on response, and incorporated into a Cox model to assess its effect on OS or PFS after Day 21. These analyses will be conducted for GT103 monotherapy.

Assuming a normally distributed measure for each biomarker, a mean change from baseline will be computed. Regression methods will be used to assess the effect of drug dose on the change. The impact of these changes on response will be explored using logistic regression. The effect of dose, and an interaction between dose and change, may also be incorporated into the model.

The impact of these changes on OS and PFS will be explored using a Cox proportional hazards model. Assuming an adequate number of events, dose and an interaction between dose and change may be incorporated into this model. For these analyses, OS and PFS will be defined from the landmark of Day 21.

9.6.2 Exploratory Objective #2: Tumor-Based Biomarkers

We intend to explore factors that may identify which patients will respond to GT103 therapy based on tumor susceptibility to CDC. Specifically, we will examine the impact of the following tumor-based markers on radiographic response, survival, and progression-free survival: levels of the complement regulatory proteins CD55, CD59 and CFH.

We hypothesize that relatively low tumor cell levels of CD55 and CD59 will correlate with responsiveness to GT103 therapy. We further hypothesize that there is an optimal level of CFH for GT103 responsiveness: the CFH target must be present for the drug to bind, leading to CDC, but as the target concentration increases, its protective effect against CDC may be difficult to overcome.

Logistic regression will assess the effect of each of these biomarkers on radiographic response (response / no response). Depending upon the actual response rate, a multivariable logistic regression may be conducted to assess the impact of the biomarker with adjustment for other factors such as dose and performance status. The Kaplan-Meier estimator will describe OS and PFS within patient subgroups defined by each biomarker. If a sufficient number of “events” are observed in the data, the joint effect of the biomarker, dose, and performance status may be explored in a multivariable Cox model. The joint effect of biomarkers may also be explored.

9.6.3 Exploratory Objective #3: Imaging Features

The imaging features that will be quantified or described in this exploratory objective will be determined as images are reviewed. The association between these images and response, and between images and occurrence of toxicity will be explored using logistic regression. The Cox model and Kaplan-Meier estimator will be used to explore associations between these image measures and survival.

9.7 Interim Analysis

No interim efficacy analyses will be conducted.

9.8 Sample Size Calculation

A maximum of 33 subjects will be treated with GT103 during the study.

10 SAFETY

All adverse events (AEs) that are identified from the first dose of study drug through 30 days post study drug stop should be recorded in the CRFs. Treatment emergent AEs will be identified from the first dose of study drug.

10.1 Adverse Events

An AE is defined as any untoward medical occurrence in a patient or clinical investigation subject administered a pharmaceutical product and which does not necessarily have to have a causal relationship with this treatment. An AE can therefore be any unfavorable and unintended sign (including an abnormal laboratory finding, for example), symptom, or disease temporally associated with the use of a medicinal product or protocol-specified procedure, whether or not considered related to the medicinal product or protocol-specified procedure. Any worsening (i.e., any clinically significant adverse change in frequency and/or intensity) of a preexisting condition that is temporally associated with the use of the supporting company product(s), is also an AE.
Changes resulting from normal growth and development that do not vary significantly in frequency or severity from expected levels are not to be considered AEs. Examples of this may include, but are not limited to, onset of menses or menopause occurring at a physiologically appropriate time.
Supporting company product includes any pharmaceutical product, biological product, device, diagnostic agent or protocol-specified procedure, whether investigational (including placebo or active

comparator medication) or marketed, manufactured by, licensed by, provided by or distributed by the supporting company for human use.

AEs may occur during the course of the use of supporting company product(s) in clinical trials or within the follow-up period specified by the protocol, or prescribed in clinical practice, from overdose (whether accidental or intentional), from abuse and from withdrawal.

Progression of the cancer under study is not considered an AE unless it is considered to be drug related by the Investigator.

AEs will be documented from the date of first dose of study drug through 30 days after the last dose of study drug. All Grade 1-5 AEs as well as special reporting circumstances, such as exposure via a parent during pregnancy or breast-feeding, overdose, medication error, misuse, abuse, off-label use or occupational exposure must be recorded on the CRF.

Attribution of AEs will be indicated as follows:

- Definite: The AE is clearly related to the study drug
- Probably: The AE is likely related to the study drug
- Possible: The AE may be related to the study drug
- Unlikely: The AE is doubtfully related to the study drug
- Unrelated: The AE is clearly NOT related to the study drug

10.2 Serious Adverse Events

An SAE is defined as any untoward medical occurrence that at any dose:

1. Results in death
2. Is immediately life-threatening (i.e., in the opinion of the Investigator, the AE places the subject at immediate risk of death; it does not include a reaction that, had it occurred in a more severe form, might have caused death)
3. Requires inpatient hospitalization or results in prolongation of an existing hospitalization. Hospitalizations do not include:
    a. Preplanned (prior to the study) hospital admissions unless the hospitalization is prolonged
    b. Planned admissions (as part of a study; e.g., routine biopsies, unrelated surgeries/ hospitalization)
    c. 23-hour re-hospitalizations
    d. Hospitalization for elective procedures
    e. Emergency room visits
4. Results in persistent or significant disability or incapacity (Note: The term "disability" refers to events that result in a substantial disruption of a subject's ability to conduct normal life function.)
5. Causes a birth defect
6. Is an important medical event (Note: The term "important medical event" refers to an event that, based upon appropriate medical judgment, may not be immediately life-threatening or result in death or hospitalization, but may jeopardize the subject or require intervention to prevent one of the other serious outcomes listed under the definition of SAE. Examples of

important medical events include intensive treatment in an emergency room or at home for allergic bronchospasm; blood dyscrasias, or convulsions that do not result in hospitalization; or development of product dependency or product abuse.)

Unexpected Adverse Event (UAE): Any adverse drug experience or adverse reaction, the specificity or severity of which is not consistent with the applicable product information (e.g., the current Investigator's Brochure for an unapproved investigational product or package insert/summary of product characteristics for an approved product); or if the drug label is not required or available, the specificity or severity of which is not consistent with the risk information described in the general investigational plan or elsewhere in the current application, as amended.

Related/Associated: There is a reasonable possibility (more likely than not) that the event may have been caused by the drug, device, or research. Determining the possible cause of an event includes assessing temporal relationships, dechallenge/rechallenge information, associated (or lack of association) with underlying diseases, and the presence (or absence) of a more likely cause.

SAE Reporting Procedure for Investigators:

SAEs and/or follow up to SAEs including death due to any cause other than progression of the cancer under study, that occurs from the date of the first dose of study drug through 30 days following the last dose of study drug, whether or not related to study drug(s), must be recorded on the CRF and must be reported within 24 hours of learning of the event to MiSTic multisite coordinator and Thoracic regulatory coordinator and within 2 working days to supporting companies.

For Duke, complete the DCI SAE Report form for Investigator Initiated Trials which can be found on the Duke Cancer Institute Intranet. Submit form to DCI-MultiSiteStudies@duke.edu and thoracic-regulatory-multisite@dm.duke.edu within 24 hours of learning of event.

Participating sites will complete the DCI SAE Report Form for Investigator-Initiated Trials and submit to DCI-MultiSiteStudies@duke.edu and thoracic-regulatory-multisite@dm.duke.edu within 24 hours of learning of event. De-identified source documentation (i.e. admission notes, discharge summary, applicable laboratory results, radiology/diagnostic testing results, etc.) must be sent with the SAE Report Form. All SAEs must be followed until resolution, return to baseline condition, or deemed unresolvable. Any SAEs that are ongoing at the time the clinical database is locked will be reported to supporting companies as unresolved.

The initial report for each SAE or death should include at minimum the following information:

- protocol number and title
- patient initials, study identification number, sex, age
- date the event occurred
- description of the event
- seriousness criteria
- event causality or causal relationship
- study drug name(s)
- dose level and cycle number at the time the event occurred
- description of the patient's condition
- study status of patient at time of report
- responsible investigator name and contact details

The Investigator should report a diagnosis or a syndrome rather than individual signs or symptoms. The Investigator should separate a primary AE considered as the foremost untoward medical occurrence from secondary AEs which occurred as complications. Whenever possible, the Investigator should also provide the batch or lot number of the study drug(s).

Follow-up information should be communicated to Duke as soon as possible.
In accordance with applicable regulations, Investigators must report SAEs to their local IRB according to their institutional guidelines.

SAE Reporting Procedure for Principal Investigator and MiSTiC multisite team:

The Principal Investigator will review the report form with accompanying source documents and the Principal/Medical Monitor Review Assessment.
If the event meets the Duke University Health System (DUHS) IRB reporting requirements, the Thoracic regulatory coordinator will submit information about the SAE including the Lead PI's assessment as a safety event to the DUHS IRB.

Within two business days of receipt, the MiSTiC Multisite-coordinator or Thoracic regulatory coordinator will also submit the SAE report form and other relevant safety information to the following supporting companies:

Grid Therapeutics, LLC
Phone: 919-987-7190
Fax: TBD
For medical emergencies contact: Scott Lyman

Expedited Reporting Procedure for Duke Cancer Institute (Coordinating Center):

Duke Cancer Institute as the coordinating center for this study is responsible for reporting SAEs to the FDA in accordance with 21CFR 312.32. Any SAE that is possibly related and unexpected must be submitted to the FDA attached to the IND. If the SAE meets criteria for reporting to the FDA, the MiSTiC multisite coordinator will complete the Form FDA 3500A (MedWatch) and send to the Lead PI and supporting companies. The MiSTiC multisite team will forward FDA 3500A MedWatch form to FDA and the supporting companies that are noted above. This submission of the Form FDA 3500A to the FDA attached to the IND will be completed by the Duke Thoracic Oncology Clinical Trials Regulatory Coordinator.

1. All unexpected, drug related SAEs that are fatal or life-threatening will be reported to the FDA by phone or fax within 7 calendar days of initial receipt of the information and will provide a complete report within 8 days of the initial report submission (by calendar day 15).
2. All unexpected, treatment-related SAEs that are not fatal or life-threatening will be reported in a written report to the FDA within 15 days of initial receipt of the information.

Our Thoracic regulatory team will forward all expedited reports to all participating investigators in the form of an Investigator Alert.

10.3 Events of Clinical Interest

The following select (non-serious and serious) AEs that occur from the date of the first dose of study drug through 30 days following the last dose of study drug will be considered to be Events of Clinical Interest (ECI): pregnancy, overdose, potential drug-induced liver injury, or additional cancer diagnoses.

See Sections 10.4.1, 10.4.2, and 10.4.3 for ECI definitions.

ECI must be recorded on the CRF and must be reported within 24 hours to the MiSTiC-multisite coordinator and Thoracic regulatory coordinator within 2 working days to Grid Therapeutics.

ECI Reporting Procedure for Investigators:
Immediately upon awareness of an ECI, the Investigator (or designee)
For Duke, complete the DCI SAE Report form for Investigator Initiated Trials which can be found on the Duke Cancer Institute Intranet. Submit form to at DCI-multisitestudies@duke.edu and thoracic-regulatory-multisite@dm.duke.edu within 24 hours of learning of event.

Participating sites will complete the DCI SAE Report Form for Investigator-Initiated Trials (pages 1-4) and submit to DCI-multisitestudies@duke.edu and thoracic-regulatory-multisite@dm.duke.edu within 24 hours of learning of event. De-identified source documentation (i.e. admission notes, discharge summary, applicable laboratory results, radiology/diagnostic testing results, etc.) must be sent with the SAE Report Form.

Note: It is imperative that initial ECI reports are submitted as soon as possible (within 24 hours of knowledge of the event) with available information to the MiSTiC-multisite coordinator and Thoracic regulatory coordinator. Missing and/or clarified event information may be provided in a follow-up report.

Follow-up information including severity, action taken, concomitant medications, and outcome should be communicated to MiSTiC-multisite coordinator and Thoracic regulatory coordinator as soon as possible using the DCI SAE Report form.

ECI Reporting Procedure for Principal Investigator and MiSTiC multisite team:

The Principal Investigator will review the DCI SAE report form with accompanying source documents and complete the Principal/Medical Monitor Review Assessment.

Within two business days of receipt, the MiSTiC-multisite coordinator or Thoracic regulatory coordinator will also submit the SAE report form and other relevant safety information to the following supporting company:

Grid Therapeutics, LLC
Phone: 919-987-7190
Fax: TBD
For medical emergencies contact: Scott Lyman

10.4 Other Safety Considerations

The Investigator must also report in the same timelines as SAEs any incidence of medication error, occupational exposure, abuse or misuse that is associated with or result in an AE. All related fatal outcomes must also be reported in the same timeline as an SAE.

Refer to SAE reporting procedures in Section 10.2.

10.4.1 Pregnancy and Lactation

During the course of the trial, all female subjects of childbearing potential should be instructed to contact the treating physician immediately if they suspect that they might have conceived a child. In addition, a missed or late menstrual period should be reported to the treating physician. If a female subject, or an investigator, suspects a pregnancy prior to the administration of study drugs, the study drugs must be withheld until the results of a pregnancy test are available. If pregnancy is confirmed the patient must not receive study medications and must be withdrawn from the study. Grid Therapeutics will be informed if a pregnancy occurs.

Throughout the entire pregnancy, additional contact should be made with the subject, and in some cases with the healthcare provider, to identify spontaneous abortions and elective terminations, as well as any medical reasons for elective termination. In addition, the study investigator should include perinatal and neonatal outcome. Infants should be followed for a minimum of 8 weeks.

If a male subject is suspected of having fathered a child while on study drugs, the pregnant female partner must be notified and counseled regarding the possible risk to the fetus. In addition, the treating physician must follow the course of the pregnancy, including prenatal and neonatal outcome. Infants should be followed for a minimum of 8 weeks.

All serious AE reports relating to the pregnancy, including spontaneous abortion, elective abortion and congenital anomalies should be forwarded to the MiSTiC team and from there to Grid Therapeutics.

Such events must be reported in the same procedure as an SAE. Refer to SAE reporting procedures in Section 10.2.

10.4.2 Overdose

An overdose is defined as the accidental or intentional administration of any dose of a product that is considered both excessive and medically important. All occurrences of overdose must be reported as an SAE.

In the event of an overdose, the investigator/treating physician should:

1. Contact PI and GRID Therapeutics
2. Closely monitor the participant for AEs/SAEs and laboratory abnormalities
3. Document the quantity of the excess dose as well as the duration of the overdosing in the CRF.

10.4.3 Potential Drug Induced Liver Injury (DILI)

Wherever possible, timely confirmation of initial liver-related laboratory abnormalities should occur prior to the reporting of a potential DILI event. All occurrences of potential DILIs meeting the defined criteria must be reported as an SAE.

Potential drug induced liver injury is defined as:

1. ALT or AST elevation > 3x ULN, **AND**
2. Total bilirubin > 2x ULN, without initial findings of cholestasis (elevated serum alkaline phosphatase), **AND**
3. No other immediately apparent possible causes ALT/AST elevation and hyperbilirubinemia, including but not limited to viral hepatitis, pre-existing chronic or acute liver disease, or the administration of other drug(s) known to be hepatotoxic.

11 ADMINISTRATIVE RESPONSIBILITIES

11.1 Institutional Review Board/Independent Ethics Committee

Before study initiation, the Investigator must have written and dated approval/favorable opinion from the IRB/IEC for the protocol, consent form, subject recruitment materials/process (e.g., advertisements), and any other written information to be provided to subjects.

The Investigator should provide the IRB/IEC with reports, updates, and other information (e.g., Safety Updates, Amendment IRB/IECs, and Administrative Letters) according to regulatory requirements and institution procedures.

Copies of all IRB/IEC approvals, as well as annual re-approvals and approved/stamped informed consent forms must be submitted to Duke Thoracic Oncology Clinical Trials Office.

11.2 Protocol and Protocol Revisions

All revisions to the protocol will be provided to the supporting companies by the PI or designee(s) at the Duke Thoracic Oncology Clinical Trials Office.

Investigators must obtain written and dated and approval/favorable opinion from the IRB/IEC before conducting any updated protocol version. Study must be conducted as described in the approved protocol. The Investigator must not implement changes of the approved protocol without prior written agreement by the Lead PI and prior review and documented approval/favorable agreement by the IRB/IEC of an amendment, except where necessary to eliminate an immediate hazard(s) to study subjects, or when the changes involve only logistical or administrative aspects of the study (e.g., changes in research personnel or change in phone numbers).

Documentation of approval(s) from the IRB/IEC must be sent to Duke Thoracic Oncology Clinical Trials Office.

11.3 Protocol Deviations and Violations

A protocol deviation is non-adherence to protocol specific study procedures or schedules that does not involve inclusion/exclusion criteria, primary objective evaluation criteria, and/or Good Clinical Practice (GCP) guidelines.

A protocol violation is any significant divergence from the protocol such as non-adherence on the part of the subject, the Investigator, or the sponsor to protocol specific inclusion/exclusion criteria, primary objective evaluation criteria, and/or GCP guidelines.

As a matter of policy, the Lead PI (i.e., sponsor) will not grant exceptions to protocol specific entry criteria to allow subjects to enter a study. If it is found that a subject who did not meet protocol eligibility criteria was entered in a study (a protocol violation), the Lead PI and/or designee(s) at the Duke Thoracic Oncology Clinical Trials Office must be informed immediately. Such subjects will be discontinued from the study, except in an exceptional instance following review and written approval by the Lead PI and the responsible IRB/IEC, and Grid Therapeutics.

Protocol deviations and violations must be documented and reported to the PI and/or designee(s) at the Thoracic Oncology Clinical Trials Office.

In accordance with applicable regulations, Investigators must report protocol deviations and violations to their local IRB/IEC according to their institutional guidelines.

11.4 Informed Consent

The Investigator must ensure that subjects or their legally acceptable representatives are clearly and fully informed about the purpose, potential risks and other critical issues regarding clinical trials in which they volunteer to participate. Preparation of the consent form is the responsibility of the Investigator and must include all elements required by CFR 21 Part 50.25 and their IRB. A copy of the proposed informed consent document must be submitted to the Lead PI or designee(s) at the Duke Thoracic Oncology Clinical Trials Office for review and comment prior to submission to the local IRB/IEC.

Informed consent must be obtained prior to performing any study-related procedures that are not part of normal subject care, including screening and changes in medications. A copy of the signed informed consent form must be given to the study subject.

11.5 Source and Study Documentation

Source documents include all original recordings of observations or notations of clinical activities and all reports and records necessary for the evaluation and reconstruction of the clinical study. Accordingly, source documents include, but are not limited to, laboratory reports (including normal and abnormal results), radiology reports, subject diaries, biopsy reports, ultrasound photographs, subject progress notes, hospital charts or pharmacy records and any other similar reports or records of any procedure performed in accordance with the protocol.

Whenever possible, the original recording of an observation should be retained as the source document; however, a photocopy is acceptable provided that it is a clear, legible, and exact duplication of the original certified document.

When clinical observations are entered directly into an electronic medical record system (i.e., in lieu of original hardcopy records), the electronic record can serve as the source document if the system has

must be validated to meet the FDA requirements for electronic records and signatures (i.e., meets 21 CFR Part 11 (https://www.accessdata.fda.gov/scripts/cdrh/cfdocs/cfcfr/cfrsearch.cfm?cfrpart=11).

Regulations require that Investigators maintain information in the study subject's medical records which corroborate data recorded on the CRF. In order to comply with these regulatory requirements, the following information will be maintained and made available as required by the Lead PI or designee(s), monitors, and/or regulatory inspectors:

- Medical history/physical condition of the study subject prior to involvement in the study sufficient to verify protocol entry criteria
- Dated note that informed consent was obtained for the subject's participation in the study
- Dated and signed notes for each subject visit including results of examinations
- Notations on abnormal lab results and their resolution
- Dated reports of special assessments (e.g., ECG reports)
- Dated and signed notes regarding AEs (including event description, severity, onset date, duration, relation to study treatment, outcome and treatment for AE)
- Dated notes regarding concomitant medications taken during the study (including start and stop dates)
- Subject condition upon completion of or withdrawal from the study

Study documentation includes all CRFs, data correction forms, source documents, monitoring logs and appointment schedules, sponsor-investigator correspondence and regulatory documents (e.g., protocol and amendments, IRB/IEC correspondence and approvals, approved and signed subject consent forms, Statement of Investigator form, and clinical study supplies receipts and distribution records).

The Investigator will prepare and maintain complete and accurate study documentation in compliance with GCP guidelines and applicable federal, state, and local laws, rules and regulations; and, for each subject participating in the study, promptly complete all CRFs and such other reports as required by this protocol following completion or termination of the clinical study or as otherwise required pursuant to any agreement with the Lead PI and the DCI.

The Investigator acknowledges that, within legal and regulatory restrictions and institutional and ethical considerations, study documentation will be promptly and fully disclosed to Lead PI or designee(s) by the Investigator upon request and also shall be made available at the Investigator's site upon request for inspection, copying, review and audit at reasonable times by representatives of the Lead PI and DCI or responsible government agencies as required by law.

The Investigator agrees to promptly take any reasonable steps that are requested by the Lead PI or designee(s) as a result of an audit to cure deficiencies in the study documentation and case report forms.

11.6 Case Report Forms

Subject data (i.e., completed CRFs) will be entered into an electronic data capture (EDC) system called Advarra EDC. This database is maintained on a secure Duke University server and is accessible via internet with login and password.

CRFs should be completed by trained study personnel according to guidelines provided by the Lead PI or designee(s) at the Duke MiSTiC Oncology Clinical Trials Office. The Investigator or qualified designee is responsible for recording and verifying the accuracy of subject data. The Investigator acknowledges that his/her electronic signature is the legally binding equivalent of a written signature.

By entering his/her electronic signature, the Investigator confirms that all recorded data have been verified as accurate.

In the event of discrepant data, the study monitor or study designee will request data clarification from the Investigator or designee for which may be resolved electronically in the EDC system.

Accurate and reliable data collection will be ensured through verification and crosscheck of the CRFs against the Investigator's study records (source document verification) by the study monitor or study designee.

11.7 Monitoring and Audits/Inspections

This clinical research study will be monitored both internally by the PI, and institutionally by the Duke Cancer Institute (DCI). In terms of internal review the PI will continuously monitor and tabulate AEs. Appropriate reporting to the Duke University Medical Center IRB will be made. If an unexpected frequency of Grade III or IV events occur, depending on their nature, action appropriate to the nature and frequency of these AEs will be taken. This may require a protocol amendment, dose de-escalation, or potentially closure of the study. The PI of this study will also continuously monitor the conduct, data, and safety of this study to ensure that:

- Interim analyses occur as scheduled;
- Stopping rules for toxicity and/or response are met;
- Risk/benefit ratio is not altered to the detriment of the subjects;
- Appropriate internal monitoring of AEs and outcomes is done;
- Over-accrual does not occur;
- Under-accrual is addressed with appropriate amendments or actions;
- Data are being appropriately collected in a reasonably timely manner.

DCI Protocol Review and Monitoring systems (PRMS) review of this protocol begins with an initial review by the Cancer Protocol Committee (CPC). CPC new protocol review focuses on scientific relevance, study design, adequacy of biostatistical input, protocol prioritization, feasibility of completing the study within a reasonable time frame and risk assessment of the trial. The PI will abide by CPC assessment of the level of risk, which will determine the intensity of subsequent DCI monitoring. CPC also conducts annual scientific progress reviews on protocols that are open to enrollment and focus on protocol prioritization, accrual and scientific progress. These reviews are conducted at the time of IRB annual renewals and documentation of all CPC reviews will be maintained in eIRB/iRIS systems.

A determination for the degree of monitoring conducted by the DCI monitoring team is made at the time of initial CPC approval commensurate with the type and level of intervention, phase, endpoints, degree of risk, size and complexity of the protocol. A formal, independent monitoring will be conducted by the DCI monitoring team according to the risk level and monitoring plan assigned by the CPC until the study is closed to enrollment or subjects are no longer receiving study drug or other

interventions that are more than minimal risk. Additional monitoring may be prompted by findings from monitoring visits, unexpected frequency of serious and/or unexpected toxicities, or other concerns. Monitoring visits may also be initiated upon request by DUHS and DCI Leadership, CPC, SOC, a sponsor, an investigator, or the IRB.

The DCI monitoring team reviews the adequacy of informed consent, enrollment of eligible patients, implementation of protocol-specified procedures and treatment, adequacy of data collection, and appropriateness of AE monitoring and reporting. The DCI monitoring team presents final monitoring reports to the DCI Safety Oversight Committee (SOC) highlighting safety concerns and unresolved issues. The SOC, at a convened meeting, assigns an overall rating of satisfactory, marginal, or unsatisfactory to reflect the overall quality of data, regulatory, consent, eligibility, study conduct and AE reporting. Corrective action plans (CAPs) are developed, implemented, and evaluated as indicated. The SOC will notify the sponsor-investigator and DUHS IRB when significant safety concerns are identified.

The SOC in concert with DCI monitoring team conducts data and safety monitoring for DUHS sponsor-investigator phase I and II, therapeutic interventional oncology studies that do not have an independent DSMB. These reviews occur at a minimum annually and more frequently for the high risk studies. The SOC safety reviews include review of safety data, enrollment status, stopping rules if applicable, accrual, toxicities, reference literature, and interim analyses as provided by the sponsor-investigator. The SOC, at a convened meeting, assigns a rating of satisfactory when adequate accrual with lack of excessive toxicity is present.

DCI Quality Assurance personnel, or designee, may conduct audits. Audits will include, but not be limited to: audit trail of data handling and processes, SOPs, drug supply, presence of required documents, the informed consent process, and comparison of CRFs/database with source documents. The Investigator agrees to accommodate and participate in audits conducted at a reasonable time in a reasonable manner, as needed.

Regulatory authorities may also audit an Investigator during or after the study. The Investigator should contact the Lead PI and designee(s) at the Duke Thoracic Oncology Clinical Trials Office as well as their local IRB, immediately if this occurs, and must fully cooperate with governmental (e.g., FDA) audits conducted at a reasonable time in a reasonable manner.

The Duke University Compliance Program - Human Subject Research Compliance (HSRC) section may conduct confidential audits to evaluate compliance with the protocol and the principles of GCP. The Lead PI agrees to allow the HSRC auditor(s) direct access to all relevant documents and to allocate his/her time and the time of the study team at the Duke MiSTiC Oncology Clinical Trials Office to the CTQA auditor(s) in order to discuss findings and any relevant issues.

11.8 Data Safety Monitoring Board

The safety of this clinical study will be closely monitored on an ongoing basis by an external DSMB. The DSMB will comprise of a minimum of 2 physicians and a statistician. Safety reviews will be carried out in an unblinded manner. The DSMB may recommend that subjects should/should not continue to be exposed to a treatment that may not be providing benefit at any time during the study.

The DSMB will report back to the sponsor with any recommendations regarding modifications to the study. Further details will be provided in a separate DSMB Charter.

11.9 Study Closeout

Upon completion of the study (defined as all subjects have completed all follow-up visits, all CRFs are complete, and all queries have been resolved) the Lead PI or designee(s) at the Duke MiSTiCOncology Clinical Trials Office will notify the Investigator of closeout and a study closeout visit will be performed.

The study monitor or study designee will ensure that the Investigator's regulatory files are up to date and complete, and that any outstanding issues from previous visits have been resolved. Other issues to be reviewed at the closeout visit include: retention of study files, possibility of site audits, publication policy, and study closure with local IRB.

11.9.1 Records Retention

The Investigator will maintain the records of study drug disposition, worksheets and all other study-specific documentation (e.g., study files, source documentation) until notified by the Lead PI or designee(s) at the Duke MiSTiC Oncology Clinical Trials Office that records may be destroyed. If the application is not filed or is withdrawn, the Investigator will maintain the records for at least two (2) years after the formal discontinuation of the clinical development program for this product(s).

To avoid error, the Investigator will contact the Lead PI or designee(s) at the Duke MiSTiC Oncology Clinical Trials Office before the destruction of any records pertaining to the study to ensure they no longer need to be retained. In addition, the Lead PI or designee(s) will be contacted if the Investigator plans to leave the institution so that arrangements can be made for the transfer of records.

12 REFERENCES

1. Amornsiripanitch, N., et al., *Complement factor H autoantibodies are associated with early stage NSCLC.* Clin Cancer Res, 2010. **16**(12): p. 3226-31.
2. Ferreira, V.P., M.K. Pangburn, and C. Cortes, *Complement control protein factor H: the good, the bad, and the inadequate.* Mol Immunol, 2010. **47**(13): p. 2187-97.
3. Makou, E., A.P. Herbert, and P.N. Barlow, *Functional anatomy of complement factor H.* Biochemistry, 2013. **52**(23): p. 3949-62.
4. Ajona, D., et al., *Down-regulation of human complement factor H sensitizes non-small cell lung cancer cells to complement attack and reduces in vivo tumor growth.* J Immunol, 2007. **178**(9): p. 5991-8.
5. Junnikkala, S., et al., *Exceptional resistance of human H2 glioblastoma cells to complement-mediated killing by expression and utilization of factor H and factor H-like protein 1.* J Immunol, 2000. **164**(11): p. 6075-81.
6. Wilczek, E., et al., *The possible role of factor H in colon cancer resistance to complement attack.* Int J Cancer, 2008. **122**(9): p. 2030-7.
7. Varsano, S., et al., *Human lung cancer cell lines express cell membrane complement inhibitory proteins and are extremely resistant to complement-mediated lysis; a comparison with normal human respiratory epithelium in vitro, and an insight into mechanism(s) of resistance.* Clin Exp Immunol, 1998. **113**(2): p. 173-82.
8. Klos, A., et al., *The role of the anaphylatoxins in health and disease.* Mol Immunol, 2009. **46**(14): p. 2753-66.
9. Campa, M.J., et al., *Complement Factor H Antibodies from Lung Cancer Patients Induce Complement-Dependent Lysis of Tumor Cells, Suggesting a Novel Immunotherapeutic Strategy.* Cancer Immunol Res, 2015. **3**(12): p. 1325-32.
10. Morgan, H.P., et al., *Structural basis for engagement by complement factor H of C3b on a self surface.* Nat Struct Mol Biol, 2011. **18**(4): p. 463-70.
11. Hofer, J., T. Giner, and M. Jozsi, *Complement factor H-antibody-associated hemolytic uremic syndrome: pathogenesis, clinical presentation, and treatment.* Semin Thromb Hemost, 2014. **40**(4): p. 431-43.
12. Bushey, R.T., et al., *A Therapeutic Antibody for Cancer, Derived from Single Human B Cells.* Cell Rep, 2016. **15**(7): p. 1505-13.
13. Herbert, A.P., et al., *Structural and functional characterization of the product of disease-related factor H gene conversion.* Biochemistry, 2012. **51**(9): p. 1874-84.
14. Kajander, T., et al., *Dual interaction of factor H with C3d and glycosaminoglycans in host-nonhost discrimination by complement.* Proc Natl Acad Sci U S A, 2011. **108**(7): p. 2897-902.
15. Morgan, H.P., et al., *Structural analysis of the C-terminal region (modules 18-20) of complement regulator factor H (FH).* PLoS One, 2012. **7**(2): p. e32187.
16. Ruivo, C.F., et al., *The Biology of Cancer Exosomes: Insights and New Perspectives.* Cancer Res, 2017. **77**(23): p. 6480-6488.
17. Zhou, L., et al., *The biology, function and clinical implications of exosomes in lung cancer.* Cancer Lett, 2017. **407**: p. 84-92.
18. Liu, S., et al., *Roles of exosomes in the carcinogenesis and clinical therapy of non-small cell lung cancer.* Biomed Pharmacother, 2019. **111**: p. 338-346.
19. Chen, G., et al., *Exosomal PD-L1 contributes to immunosuppression and is associated with anti-PD-1 response.* Nature, 2018. **560**(7718): p. 382-386.

20. Poggio, M., et al., *Suppression of Exosomal PD-L1 Induces Systemic Anti-tumor Immunity and Memory.* Cell, 2019. **177**(2): p. 414-427 e13.
21. Bigner, S.H., et al., *Characterization of the epidermal growth factor receptor in human glioma cell lines and xenografts.* Cancer Res, 1990. **50**(24): p. 8017-22.
22. Pickering, M.C., et al., *Uncontrolled C3 activation causes membranoproliferative glomerulonephritis in mice deficient in complement factor H.* Nat Genet, 2002. **31**(4): p. 424-8.
23. Pickering, M.C., et al., *Spontaneous hemolytic uremic syndrome triggered by complement factor H lacking surface recognition domains.* J Exp Med, 2007. **204**(6): p. 1249-56.
24. Brahmer, J.R., et al., *Management of Immune-Related Adverse Events in Patients Treated With Immune Checkpoint Inhibitor Therapy: American Society of Clinical Oncology Clinical Practice Guideline.* J Clin Oncol, 2018. **36**(17): p. 1714-1768.
25. Eisenhauer, E.A., et al., *New response evaluation criteria in solid tumours: revised RECIST guideline (version 1.1).* Eur J Cancer, 2009. **45**(2): p. 228-47.
26. Oken, M.M., et al., *Toxicity and response criteria of the Eastern Cooperative Oncology Group.* Am J Clin Oncol, 1982. **5**(6): p. 649-55.

Appendix A. RECIST 1.1

RECIST version 1.1 [25] will be used in this study for assessment of tumor response. While either CT or MRI may be utilized, as per RECIST 1.1, CT is the preferred imaging technique in this study.

Definitions

Response and progression will be evaluated in this study using the international criteria proposed by the RECIST Committee (version 1.1). Changes in only the largest diameter (unidimensional measurement) of the tumor lesions are used in the RECIST criteria. Note: Lesions are either measurable or non-measurable using the criteria provided below. The term "evaluable" in reference to measurability will not be used because it does not provide additional meaning or accuracy.

Measurable Disease

Tumor lesions: Must be accurately measured in at least one dimension (longest diameter in the plane of measurement is to be recorded) with a minimum size of:

- 10mm by CT scan (irrespective of scanner type) and MRI (no less than double the slice thickness and a minimum of 10mm)
- 10mm caliper measurement by clinical exam (when superficial)
- 20mm by chest X-ray (if clearly defined and surrounded by aerated lung)

Malignant lymph nodes: To be considered pathologically enlarged and measurable, a lymph node must be ≥15mm in short axis when assessed by CT scan (CT scan slice thickness recommended to be no greater than 5 mm). At baseline and in follow-up, only the short axis will be measured and followed.

Non-measurable Disease

All other lesions (or sites of disease), including small lesions (longest diameter ≥10 to <15 mm with conventional techniques or <10 mm using spiral CT scan), are considered non-measurable disease. Leptomeningeal disease, ascites, pleural or pericardial effusion, inflammatory breast disease, lymphangitic involvement of skin or lung, abdominal masses/abdominal organomegaly identified by physical exam that is not measurable by reproducible imaging techniques are all non-measurable.

Bone lesions:

- Bone scan, PET scan or plain films are not considered adequate imaging techniques to measure bone lesions. However, these techniques can be used to confirm the presence or disappearance of bone lesions.
- Lytic bone lesions or mixed lytic-blastic lesions, with identifiable soft tissue components, that can be evaluated by cross sectional imaging techniques such as CT or MRI can be considered as measurable lesions if the soft tissue component meets the definition of measurability described above.
- Blastic bone lesions are non-measurable.

Cystic lesions:

- Lesions that meet the criteria for radiographically defined simple cysts should not be considered as malignant lesions (neither measurable nor non-measurable) since they are, by definition, simple cysts.

- ‘Cystic lesions’ thought to represent cystic metastases can be considered as measurable lesions, if they meet the definition of measurability described above. However, if noncystic lesions are present in the same patient, these are preferred for selection as target lesions.

Lesions with prior local treatment:

- Tumor lesions situated in a previously irradiated area, or in an area subjected to other loco-regional therapy, are usually not considered measurable unless there has been demonstrated progression in the lesion. Study protocols should detail the conditions under which such lesions would be considered measurable.

Target Lesions

All measurable lesions up to a maximum of 2 lesions per organ and 5 lesions in total, should be identified as target lesions and recorded and measured at baseline. Target lesions should be selected on the basis of their size (lesions with the longest diameter), be representative of all involved organ, but in addition should be those that lend themselves to reproducible repeated measurements.

Lymph nodes merit special mention since they are normal anatomical structures which may be visible by imaging even if not involved by tumor. Pathological nodes which are defined as measurable and may be identified as target lesions must meet the criterion of a short axis of ≥15mm by CT scan. Only the short axis of these nodes will contribute to the baseline sum. The short axis of the node is the diameter normally used by radiologists to judge if a node is involved by solid tumor. Nodal size is normally reported as two dimensions in the plane in which the image is obtained (for CT scan this is almost always the axial plane; for MRI the plane of acquisition may be axial, sagittal or coronal). The smaller of these measures is the short axis. For example, an abdominal node which is reported as being 20mm x 30mm has a short axis of 20mm and qualifies as a malignant, measurable node. In this example, 20mm should be recorded as the node measurement. All other pathological nodes (those with short axis ≥10mm but <15 mm) should be considered non-target lesions. Nodes that have a short axis <10mm are considered non-pathological and should not be recorded or followed.

A sum of the diameters (longest for non-nodal lesions, short axis for nodal lesions) for all target lesions will be calculated and reported as the baseline sum diameters. If lymph nodes are to be included in the sum, then as noted above, only the short axis is added into the sum. The baseline sum diameters will be used as reference to further characterize any objective tumor regression in the measurable dimension of the disease.

Non-target Lesions

All other lesions (or sites of disease) including pathological lymph nodes should be identified as non-target lesions and should also be recorded at baseline. Measurements are not required and these lesions should be followed as ‘present’, ‘absent’, or in rare cases ‘unequivocal progression’ (more details to follow). In addition, it is possible to record multiple non-target lesions involving the same organ as a single item on the case record form (e.g., ‘multiple enlarged pelvic lymph nodes’ or ‘multiple liver metastases’).

Guidelines for Evaluation of Measurable Disease
All measurements should be recorded in metric notation, using calipers if clinically assessed. All baseline evaluations should be performed as close as possible to the treatment start and never more than 4 weeks before the beginning of the treatment.

The same method of assessment and the same technique should be used to characterize each identified and reported lesion at baseline and during follow-up. Imaging based evaluation should always be done rather than clinical examination unless the lesion(s) being followed cannot be imaged but are assessable by clinical exam.

Clinical lesions: Clinical lesions will only be considered measurable when they are superficial and >10mm diameter as assessed using calipers (e.g., skin nodules). For the case of skin lesions, documentation by color photography including a ruler to estimate the size of the lesion is suggested. As noted above, when lesions can be evaluated by both clinical exam and imaging, imaging evaluation should be undertaken since it is more objective and may also be reviewed at the end of the study.

Chest X-ray: Chest CT is preferred over chest X-ray, particularly when progression is an important endpoint, since CT is more sensitive than X-ray, particularly in identifying new lesions. However, lesions on chest X-ray may be considered measurable if they are clearly defined and surrounded by aerated lung.

CT, MRI: CT is the best currently available and reproducible method to measure lesions selected for response assessment. This guideline has defined measurability of lesions on CT scan based on the assumption that CT slice thickness is 5mm or less. When CT scans have slice thickness greater than 5 mm, the minimum size for a measurable lesion should be twice the slice thickness. MRI is also acceptable in certain situations (e.g., for body scans).

Ultrasound: Ultrasound is not useful in assessment of lesion size and should not be used as a method of measurement. Ultrasound examinations cannot be reproduced in their entirety for independent review at a later date and, because they are operator dependent, it cannot be guaranteed that the same technique and measurements will be taken from one assessment to the next. If new lesions are identified by ultrasound in the course of the study, confirmation by CT or MRI is advised. If there is concern about radiation exposure from CT, MRI may be used instead of CT in selected instances.

Endoscopy, laparoscopy: The utilization of these techniques for objective tumor evaluation is not advised. However, they can be useful to confirm complete pathological response when biopsies are obtained or to determine relapse in trials where recurrence following complete response or surgical resection is an endpoint.

Tumor markers: Tumor markers alone cannot be used to assess objective tumor response. If markers are initially above the upper normal limit, however, they must normalize for a patient to be considered in complete response. Because tumor markers are disease specific, instructions for their measurement should be incorporated into protocols on a disease specific basis. Specific guidelines for both CA-125 response (in recurrent ovarian cancer) and PSA response (in recurrent prostate cancer), have been published. In addition, the Gynecologic Cancer Intergroup has developed CA125

progression criteria which are to be integrated with objective tumor assessment for use in first-line trials in ovarian cancer.

Cytology, histology: These techniques can be used to differentiate between PR and CR in rare cases if required by protocol (for example, residual lesions in tumor types such as germ cell tumors, where known residual benign tumors can remain). When effusions are known to be a potential adverse effect of treatment (e.g., with certain taxane compounds or angiogenesis inhibitors), the cytological confirmation of the neoplastic origin of any effusion that appears or worsens during treatment can be considered if the measurable tumor has met criteria for response or stable disease in order to differentiate between response (or stable disease) and progressive disease.

Response Criteria

Evaluation of Target Lesions

- Complete Response (CR): Disappearance of all target lesions. Any pathological lymph nodes (whether target or non-target) must have reduction in short axis to <10 mm.
- Partial Response (PR): At least a 30% decrease in the sum of diameters of target lesions, taking as reference the baseline sum diameters.
- Progressive Disease (PD): At least a 20% increase in the sum of diameters of target lesions, taking as reference the smallest sum on study (this includes the baseline sum if that is the smallest on study). In addition to the relative increase of 20%, the sum must also demonstrate an absolute increase of at least 5 mm. (Note: the appearance of one or more new lesions is also considered progression).
- Stable Disease (SD): Neither sufficient shrinkage to qualify for PR nor sufficient increase to qualify for PD, taking as reference the smallest sum diameters while on study.

Lymph nodes. Lymph nodes identified as target lesions should always have the actual short axis measurement recorded (measured in the same anatomical plane as the baseline examination), even if the nodes regress to below 10 mm on study. This means that when lymph nodes are included as target lesions, the 'sum' of lesions may not be zero even if complete response criteria are met, since a normal lymph node is defined as having a short axis of <10 mm. CRFs or other data collection methods may therefore be designed to have target nodal lesions recorded in a separate section where, in order to qualify for CR, each node must achieve a short axis <10 mm. For PR, SD and PD, the actual short axis measurement of the nodes is to be included in the sum of target lesions.

Target lesions that become 'too small to measure'. While on study, all lesions (nodal and non-nodal) recorded at baseline should have their actual measurements recorded at each subsequent evaluation, even when very small (e.g., 2 mm). However, sometimes lesions or lymph nodes which are recorded as target lesions at baseline become so faint on CT scan that the radiologist may not feel comfortable assigning an exact measure and may report them as being 'too small to measure'. When this occurs it is important that a value be recorded on the CRF. If it is the opinion of the radiologist that the lesion has likely disappeared, the measurement should be recorded as 0 mm. If the lesion is believed to be present and is faintly seen but too small to measure, a default value of 5 mm should be assigned (Note: It is less likely that this rule will be used for lymph nodes since they usually have a definable size when normal and are frequently surrounded by fat such as in the retroperitoneum; however, if a lymph node is believed to be present and is faintly seen but too small to measure, a default value of 5 mm should be assigned in this circumstance as well). This default value is derived from the 5 mm CT slice thickness (but should not be changed with varying CT slice thickness). The

measurement of these lesions is potentially non-reproducible, therefore providing this default value will prevent false responses or progressions based upon measurement error. To reiterate, however, if the radiologist is able to provide an actual measure, that should be recorded, even if it is below 5 mm.

Lesions that split or coalesce on treatment. When non-nodal lesions 'fragment', the longest diameters of the fragmented portions should be added together to calculate the target lesion sum. Similarly, as lesions coalesce, a plane between them may be maintained that would aid in obtaining maximal diameter measurements of each individual lesion. If the lesions have truly coalesced such that they are no longer separable, the vector of the longest diameter in this instance should be the maximal longest diameter for the 'coalesced lesion'.

Evaluation of Non-target Lesions
While some non-target lesions may actually be measurable, they need not be measured and instead should be assessed only qualitatively at the time points specified in the protocol.

- Complete Response (CR): Disappearance of all non-target lesions and normalization of tumor marker level. All lymph nodes must be non-pathological in size (<10 mm short axis).
- Non-CR/Non-PD: Persistence of one or more non-target lesion(s) and/or maintenance of tumor marker level above the normal limits.
- Progressive Disease (PD): Unequivocal progression (see comments below) of existing non-target lesions. (Note: the appearance of one or more new lesions is also considered progression).

When the patient also has measurable disease. In this setting, to achieve 'unequivocal progression' on the basis of the non-target disease, there must be an overall level of substantial worsening in non-target disease such that, even in presence of SD or PR in target disease, the overall tumor burden has increased sufficiently to merit discontinuation of therapy. A modest 'increase' in the size of one or more non-target lesions is usually not sufficient to quality for unequivocal progression status. The designation of overall progression solely on the basis of change in non-target disease in the face of SD or PR of target disease will therefore be extremely rare.

When the patient has only non-measurable disease. This circumstance arises in some phase III trials when it is not a criterion of study entry to have measurable disease. The same general concept apply here as noted above, however, in this instance there is no measurable disease assessment to factor into the interpretation of an increase in non-measurable disease burden. Because worsening in non-target disease cannot be easily quantified (by definition: if all lesions are truly non-measurable), a useful test that can be applied when assessing patients for unequivocal progression is to consider if the increase in overall disease burden based on the change in non-measurable disease is comparable in magnitude to the increase that would be required to declare PD for measurable disease; i.e., an increase in tumor burden representing an additional 73% increase in 'volume' (which is equivalent to a 20% increase diameter in a measurable lesion). Examples include an increase in a pleural effusion from 'trace' to 'large', an increase in lymphangitic disease from localized to widespread, or may be described in protocols as 'sufficient to require a change in therapy'. If 'unequivocal progression' is seen, the patient should be considered to have had overall PD at that point. While it would be ideal to have objective criteria to apply to non-measurable disease, the very nature of that disease makes it impossible to do so, therefore the increase must be substantial.

New Lesions

The appearance of new malignant lesions denotes disease progression; therefore, some comments on detection of new lesions are important. There are no specific criteria for the identification of new radiographic lesions; however, the finding of a new lesion should be unequivocal: i.e., not attributable to differences in scanning technique, change in imaging modality or findings thought to represent something other than tumor (for example, some 'new' bone lesions may be simply healing or flare of pre-existing lesions). This is particularly important when the patient's baseline lesions show partial or complete response. For example, necrosis of a liver lesion may be reported on a CT scan report as a 'new' cystic lesion, which it is not.

A lesion identified on a follow-up study in an anatomical location that was not scanned at baseline is considered a new lesion and will indicate disease progression. An example of this is the patient who has visceral disease at baseline and while on study has a CT or MRI brain ordered which reveals metastases. The patient's brain metastases are considered to be evidence of PD even if he/she did not have brain imaging at baseline.

If a new lesion is equivocal, for example because of its small size, continued therapy and follow-up evaluation will clarify if it represents truly new disease. If repeat scans confirm there is definitely a new lesion, then progression should be declared using the date of the initial scan.

While FDG-PET response assessments need additional study, it is sometimes reasonable to incorporate the use of FDG-PET scanning to complement CT scanning in assessment of progression (particularly possible 'new' disease). New lesions on the basis of FDG-PET imaging can be identified according to the following algorithm:

- Negative FDG-PET at baseline, with a positive FDG-PET at follow-up is a sign of PD based on a new lesion.
- No FDG-PET at baseline and a positive FDG-PET at follow- up: If the positive FDG-PET at follow-up corresponds to a new site of disease confirmed by CT, this is PD. If the positive FDG-PET at follow-up is not confirmed as a new site of disease on CT, additional follow-up CT scans are needed to determine if there is truly progression occurring at that site (if so, the date of PD will be the date of the initial abnormal FDG-PET scan). If the positive FDG-PET at follow-up corresponds to a pre-existing site of disease on CT that is not progressing on the basis of the anatomic images, this is not PD.

Evaluation of Best Overall Response

The best overall response is the best response recorded from the start of the study treatment until the end of treatment taking into account any requirement for confirmation. On occasion a response may not be documented until after the end of therapy, so protocols should be clear if post-treatment assessments are to be considered in determination of best overall response. Protocols must specify how any new therapy introduced before progression will affect best response designation. The patient's best overall response assignment will depend on the findings of both target and non-target disease and will also take into consideration the appearance of new lesions. Furthermore, depending on the nature of the study and the protocol requirements, it may also require confirmatory measurement. Specifically, in non-randomised trials where response is the primary endpoint, confirmation of PR or CR is needed to deem either one the "best overall response."
The best overall response is determined once all the data for the patient is known. Best response determination in trials where confirmation of complete or partial response IS NOT required: Best

response in these trials is defined as the best response across all time points (for example, a patient who has SD at first assessment, PR at second assessment, and PD on last assessment has a best overall response of PR). When SD is believed to be best response, it must also meet the protocol specified minimum time from baseline. If the minimum time is not met when SD is otherwise the best time point response, the patient's best response depends on the subsequent assessments. For example, a patient who has SD at first assessment, PD at second and does not meet minimum duration for SD, will have a best response of PD. The same patient lost to follow-up after the first SD assessment would be considered inevaluable.

Appendix B. ECOG Performance Status

The ECOG Scale of Performance Status [26], developed by the Eastern Cooperative Oncology Group, Robert L. Comis, MD, Group Chair, describes a patient's level of functioning in terms of their ability to care for themself, daily activity, and physical ability (walking, working, etc.).

Grade	Description
**0**	Normal activity. Fully active, able to carry on all pre-disease performance without restriction.
**1**	Symptoms, but ambulatory. Restricted in physically strenuous activity, but ambulatory and able to carry out work of a light or sedentary nature (e.g., light housework, office work).
**2**	In bed <50% of the time. Ambulatory and capable of all self-care, but unable to carry out any work activities. Up and about more than 50% of waking hours.
**3**	In bed >50% of the time. Capable of only limited self-care, confined to bed or chair more than 50% of waking hours.
**4**	100% bedridden. Completely disabled. Cannot carry on any self-care. Totally confined to bed or chair.
**5**	Dead

Appendix C. Laboratory Tests

CBC with Differential		
• hematocrit<br>• hemoglobin<br>• platelet count	• WBC (total and differential)<br>• red blood cell (RBC) count	• absolute neutrophil count<br>• absolute lymphocyte count
**Chemistries with Liver Function Tests (LFTs)**		
• albumin<br>• alkaline phosphatase (ALP)<br>• ALT<br>• AST<br>• bicarbonate	• blood urea nitrogen (BUN)<br>• chloride<br>• creatinine<br>• glucose<br>• calcium<br>• phosphorus	• potassium<br>• sodium<br>• total bilirubin<br>• total protein
**Thyroid Function Tests**		
• thyroid stimulating hormone (TSH)	• total triiodothyronine (T3)	• free thyroxine (T4)
**Pregnancy Test**		
• serum β-HCG pregnancy test		
**Viral Serology**		
• HIV 1 & 2<br>• HBsAG<br>• HCV Antibody		

Appendix D. Study Manual

Duke Cancer Institute –Thoracic Oncology Clinical Trials

Study Title: Phase Ib First in Human Dose Escalation of GT103 in Refractory, Advanced Stage Non-Small Cell Lung Cancer (TOP 1902)

Sample Collection Instructions

- Refer to the specific sample collection, processing and pick-up/shipping instructions for each sample type below.
- Using a Sharpie or Cryopen, the following information must be written-in on each sample label:
    - Study Short Name
    - Sample Type (*EDTA Plasma, Tissue*)
    - Subject Study ID Number
    - Subject Initials
    - Sample Collection Date
    - Sample Collection Time
    - Sample Frozen Time
- Complete the Research Sample Flow Sheet.
- For any questions regarding the research samples for this study, contact jchris.brady@duke.edu.

Plasma for Assay of PK, Complement, ADA, and Future Research

1. At designated time points, draw 3 or 4 (if consented to Future Reseach Blood) 10 ml purple top (K_2EDTA) tubes (BD Vacutainer, Catalog no. 366643)
2. Invert tubes 10 times to mix blood.
3. Process blood within 2 hours of blood draw. Document time drawn and time processed.
4. Centrifuge at 4°C at 2500 x g for 15 minutes (or in accordance with centrifuge manufacturer's instructions).
5. Draw off plasma very slowly with transfer pipette approaching no closer than 0.5 cm of the **buffy coat**, taking care NOT to disturb the buffy coat. Transfer plasma equally into **two separate clean 15 mL polypropylene tubes. One buffy coat per EDTA tube.**
    a. After removing the plasma, carefully remove the white, "buffy coat" white blood cell layer, avoiding the red blood cell mass as much as possible.
    b. Transfer the buffy coat layer (approximately 0.2 – 0.5 ml) from EDTA tube into the labeled cryovial. Immediately freeze the cryovial of buffy coat on dry ice or in liquid nitrogen vapor. Once completely frozen, the cryovial containing the buffy coat cells may be stored at -70 to -90 degrees until ready for transfer on dry ice.
6. Repeat centrifuge at 4°C at 2500 x g for 15 minutes (or in accordance with centrifuge manufacturer's instructions).
7. Draw off plasma very slowly with transfer pipette, taking care NOT to disturb the residual cell pellet at the bottom of the tube.
8. Aliquot plasma equally (as possible) into 9 cryovials per timepoint
9. Affix label marked 'EDTA PLASMA' to each cryovial.

10. Store cryovials in -80°C freezer.
11. Batch ship. Schedule pick-up by emailing Phase I Biomarker Laboratory at jchris.brady@duke.edu.

Tumor (Tissue)

If available, archived FFPE tissues will be procured, sectioned, and prepared for batch shipping at the end of the study enrollment. Immunohistochemistry will be performed using the following primary antibodies supplied by the laboratory of Dr. Patz: CD55 [EPR6689], CD59 [EPR6425(2)], OX-24 [ab118820] (Abcam, Cambridge, MA) Tissue will be collected at end of study enrollment and batch sent to Dr. Nixon's lab, Phase 1 Biomarker Laboratory.

Assays

PK Assay

Summary: We will assay GT103 in plasma using the following ELISA protocol: Biotinylated peptide containing the epitope for GT103 will be immobilized on NeutrAvidin in wells of an ELISA plate. Plasma samples will be diluted in PBS. Antibody in each diluted sample will be captured on the peptide and detected with a goat anti-human IgG gamma-chain-horseradish peroxidase (HRP) conjugate. The ELISA will be developed with the HRP substrate ABTS, the plate read at 405 nm in a plate reader, and antibody concentration determined from a standard curve composed of GT103 antibody serially diluted in DPBS.

Supplies and Reagents:
- Pep19-1(15)-biotin peptide: GPPPPIDNGDITSFPGGG{Lys(biotin)}
- Synthesized by Genscript, Piscataway, NJ and supplied as lyophilized powder.
- Dissolve at 2 mg/ml in deionized water.
- Biotin: Sigma-Aldrich catalog #B4639, 10 mg/ml in DMSO.
- NeutrAvidin coated 96-well plates
- Thermo Fisher Scientific catalog #15129
- Secondary antibody conjugate: goat anti-human IgG gamma chain-HRP
- Chemicon catalog #AP504P
- Dulbecco's phosphate-buffered saline without calcium or magnesium (DPBS)
- Thermo Fisher Scientific/Gibco catalog #14190-144
- Wash buffer: DPBS + 0.1% (v/v) Tween-20 (DPBST)
- HRP substrate: 0.22 mg/ml 2,2'-azino-bis(3-ethylbenzothiazoline-6-sulphonic acid) (ABTS) in 50 mM citric acid pH 4; add 1 µl 30% H_2O_2 per ml ABTS immediately before use

Protocol:

1. **Peptide Capture**
   a. Wash the plate in automated plate washer with DPBST, 4 x 300 µl per well. Smack inverted plate on paper towels after last wash to remove traces of buffer.
   b. Dilute peptide and biotin (for control wells) each to 2 µg/ml in DPBST.

c. Load 100 µl peptide and biotin into 3 wells each (6 wells total) for each standard curve dilution and sample being tested.
d. Incubate 30 min at room temperature (RT) on orbital shaker.
e. Wash plate with automated plate washer and remove traces of buffer as above.

2. **Sample Incubation**
   a. Add 100 µl of each sample, diluted in DPBST, in triplicate, to the peptide and biotin wells.
   b. Cover with plate tape and incubate 2 h at RT on orbital shaker.
   c. Wash plate with automated plate washer and remove traces of buffer as above.

3. **Secondary Antibody**
   a. Dilute secondary antibody 1:8000 in DPBST and add 100 µl to all wells.
   b. Cover with plate tape and incubate 1 h at RT on orbital shaker.
   c. Just after adding the secondary antibody, put some ABTS in a tube on the bench so it warms to RT. (Use the same volume used for the secondary antibody.)
   d. Wash plate with automated plate washer and remove traces of buffer as above.

4. **Detection**
   a. Add 1 µl H_2O_2 per ml of ABTS and vortex to mix.
   b. Add 100 µl/well ABTS/H_2O_2.
   c. Incubate 10 min at RT.
   d. Read at 405 nm.
   e. Subtract the biotin absorbance values from the peptide values.

Complement Assays

Summary: Complement C3a and sC5b-9 in plasma isolated from blood collected at time points during treatment will be assayed using MicroVue^{TM} Complement ELISA kits from Quidel (San Diego, CA) according to the manufacturer's instructions. Each kit contains standards for quantitation.

Anti-Drug Antibody (ADA) Assay

Sample analysis will be performed in support of the Phase 1 study. ADA in plasma isolated from blood collected at designated time points during treatment. The vendor will analyze human plasma samples for anti-GT103 antibodies using the validated method. All experiments will be performed in accordance with the FDA BMV Guidelines and current industry standards with appropriate documentation.

GT103 antibody used in the assay will be provided by Grid Therapeutics. GT103 will be provided along with safety data sheets and certificates of analysis indicating identity, concentration, storage conditions, and retest date as appropriate.

Method Development

Method development is based on an initial protocol, described in general terms below, using the established methods of MesoScale Discovery. It is a bridging ELISA that requires GT103 to be independently labeled with both biotin and SULFO-TAG (MesoScale Discovery, Rockville MD). All samples will be assayed in duplicate.

Protocol:

1. GT103 is conjugated to biotin using the Thermo Scientific EZ-Link Sulfo-NHS-Biotinylation Kit and diluted in phosphate buffered saline (PBS).

2. The biotinylated GT103 is conjugated to Sulfo-TAG using the Mesoscale Discovery MSD GOLD SULFO-TAG NHS-Ester Conjugation Pack and diluted in PBS.

3. A master-mix comprised of GT103-biotin, GT103-Sulfo-TAG, and patient plasma is placed into a 96-well polypropylene plate, and the plate is sealed and incubated for 1 h at room temperature.

4. A Streptavidin Gold plate (MesoScale Discovery) is blocked for 1 h at room temperature.

5. The wells are washed, the conjugated GT103/sample master-mix is added to the plate, and the plate is sealed and incubated with shaking for 1.5 h at room temperature.

6. The wells are washed, 2x Read Buffer T (Mesoscale Discovery) is added to each well, and the plate is read using a Model 1300 MESO QuickPlex SQ120.

Method development will entail:

- Titration of critical reagents (antibody, level of positive control, detergent and salt concentration if needed to lower the background)
- Optimization and evaluation of the key steps (incubation time and temperature, number of washes, drift and edge effect), preliminary negative cut-off determination
- Determination of matrix interference/tolerance and Minimum Required Dilution (MRD)
- Evaluation of specificity and recovery
- Evaluation of robustness (running several QCs over at least 3 days to evaluate the robustness and performance of the assay)
- Determination of drug interference/tolerance/target interference and selectivity (low, mid and high ADA QC pre-incubated with serial dilutions of drug)
- Evaluation of sensitivity (using the purified positive control antibody)
- Evaluation of prozone effect / hook effect and dilution linearity

Method Validation

The vendor will perform a full validation for the ADA assay, and a Validation Report will be provided. Method validation will entail:

- Establishment of the screening and confirmatory cut points
- Estimation of the sensitivity and precision of the assay
- Determination of selectivity and interference (especially circulating drug interference)
- Confirmation of the MRD established during development

- Assessment of robustness, and stability (of control samples and reagents, if needed)
- Establishment of the assay acceptance criteria

Appendix E. Standard Cockcroft and Gault Formula for Calculating Creatinine Clearance

Creatinine clearance (CrCl) will be calculated using the Cockcroft-Gault equation as follows:

For serum creatinine concentration in mg/dL:

$$\text{CrCl (mL/min)} = \frac{\text{(140-age in yrs) X (weight in kg)}}{\text{72 x serum creatinine (mg/dL)}}$$

For females, multiply the result by 0.85.

For serum creatinine concentration in mg/dL:

$$\text{CrCl (mL/min)} = \frac{\text{[(140-age in yrs) x (weight in kg)] x 0.85}}{\text{72 x serum creatinine (mg/dL)}}$$

Source: Cockcroft DW, Gault MH. 1976. Prediction of creatinine clearance from serum creatinine. *Nephron* 16:31-4.
